# Targeting ATM Kinase in Cancer—A Comprehensive Review

**DOI:** 10.32604/or.2026.082180

**Published:** 2026-10-28

**Authors:** Mateusz Kciuk, Gabriela Machura, Katarzyna Wanke, Piotr Gromek, Beata Marciniak, Renata Kontek

**Affiliations:** 1 Department of Molecular Biotechnology and Genetics, University of Lodz, Lodz, Poland; 2 Department of Functional Genomics, Faculty of Medicine, Medical University of Lodz, Lodz, Poland; 3 BioMedChem Doctoral School of the University of Lodz and Lodz Institutes of the Polish Academy of Sciences, Lodz, Poland; 4 Department of Inorganic and Analytical Chemistry, University of Lodz, Lodz, Poland; 5 Doctoral School of Exact and Natural Sciences, University of Lodz, Lodz, Poland; 6 Doctoral School of Molecular Medicine, Medical University of Lodz, Lodz, Poland

**Keywords:** Ataxia-telangiectasia mutated (ATM), ATM kinase, cancer, DNA damage response (DDR), inhibitor

## Abstract

Ataxia–telangiectasia mutated (ATM) is a central regulator of the DNA damage response (DDR), coordinating DNA double-strand break signaling, checkpoint activation, and maintenance of genome stability. Although traditionally regarded as a tumor suppressor, accumulating evidence indicates that many established cancers become functionally dependent on residual ATM signaling to tolerate oncogene-driven replication stress, genomic instability, oxidative stress, and defective checkpoint control. This context-dependent reliance reflects a form of non-oncogene addiction in which ATM signaling is selectively retained to sustain tumor cell survival, particularly in *TP53*-deficient and highly replication-stressed malignancies. Beyond canonical DDR functions, ATM also contributes to tumor progression through regulation of NF-κB signaling, epithelial–mesenchymal transition, hypoxia adaptation, metabolic rewiring, mitochondrial homeostasis, and immune modulation. These diverse activities provide a strong rationale for therapeutic ATM inhibition. Pharmacological ATM inhibitors impair checkpoint signaling and DNA repair, thereby sensitizing tumors to radiotherapy and DNA-damaging agents and promoting synthetic lethal interactions in DDR-deficient backgrounds. In this review, we aim to integrate current mechanistic, preclinical, and translational evidence linking ATM biology with the pharmacological and clinical development of small-molecule inhibitors, including KU-55933, KU-59403, KU-60019, CP466722, WSD0628, AZD0156, AZD1390, M3541, SYH2051, and XRD-0394. We further discuss emerging concepts such as context-specific synthetic vulnerabilities, radio-immunotherapy combinations, pharmacokinetic and toxicity considerations, mechanisms of resistance, and the limitations of current preclinical models. Collectively, ATM inhibition represents a promising precision oncology strategy for exploiting DDR dependencies and overcoming therapeutic resistance in genetically unstable cancers.

## 1 Introduction

Ataxia-telangiectasia (A-T) is a rare autosomal recessive disorder characterized by progressive neurodegeneration, immunodeficiency, cancer predisposition, particularly to hematological malignancies, and extreme sensitivity to ionizing radiation (IR), resulting from loss-of-function mutations in the ataxia-telangiectasia mutated (*ATM*) gene [1]. *ATM* encodes a ~350-kDa phosphoinositide-3-kinase–related protein kinase (PIKK) that acts as a master regulator of the DNA damage response (DDR), particularly following DNA double-strand breaks (DSBs). Early work established that ATM is essential for radiation-induced cell-cycle checkpoint activation, coordinating G1/S, intra-S, and G2/M arrest through cellular tumor antigen P53 (TP53)-dependent transcriptional induction of the cyclin-dependent kinase inhibitor 1A (P21^CIP1/WAF1^) [2].

A-T cells fail to inhibit cyclin-dependent kinase (CDK) activity after irradiation due to defective P21 accumulation, underscoring ATM’s central role in checkpoint enforcement. Genetic studies in *ATM*-deficient cells further demonstrated hypersensitivity to γ-irradiation, impaired tumor protein p53 (TP53) stabilization, defective apoptosis in thymocytes, premature senescence in fibroblasts, and intermediate checkpoint defects in heterozygous cells, collectively linking partial or complete *ATM* insufficiency to genomic instability and cancer susceptibility [3]. 

Although ATM shares structural homology with DNA-dependent protein kinase catalytic subunit (DNA-PKcs) and other PIKKs, functional analyses revealed non-redundant roles, as DNA-PKcs is dispensable for TP53 stabilization and radiation-induced checkpoint arrest, whereas ATM is absolutely required for these events [4,5,6].

Mechanistically, ATM directly phosphorylates TP53 on Ser15 in response to DSB leading to activation of downstream transcriptional programs, including P21 induction [7]. Genetic epistasis analyses demonstrated a largely linear ATM–TP53–P21 axis governing G1/S checkpoint control, while also revealing TP53-independent roles of ATM in cell growth regulation and genome maintenance [8]. 

Beyond TP53, ATM orchestrates a broad phosphorylation network involving breast cancer type 1 susceptibility protein (BRCA1) [9,10,11], checkpoint kinase 1 (CHK1) [12,13,14], checkpoint kinase 2 (CHK2) [15], histone H2AX [16,17,18,19,20], TP53-binding protein 1 (53BP1) [21,22], mediator of DNA damage checkpoint protein 1 (MDC1) [23,24,25], and replication protein A (RPA) [26,27], thereby integrating checkpoint activation, homologous recombination (HR), and ensuring replication fork stability. 

ATM also functions within a dynamic, context-dependent kinase network with ATR and DNA-PKcs, exhibiting functional redundancy in phosphorylated histone H2AX (γH2AX) formation yet temporal specialization driven by DNA end resection and replication stress (RS), enabling a coordinated ATM-to-ATR signaling switch during DSB processing [28,29,30]. 

ATM signaling is closely linked to chromatin architecture, relying on histone modifications and chromatin-associated factors to regulate DNA damage response activation [31,32,33,34,35]. Upon DNA damage, chromatin relaxation, driven by histone acetylation [36,37,38,39,40] and remodeling complexes such as SWItch/Sucrose Non-Fermentable (SWI/SNF), enhances accessibility of repair machinery and supports recruitment of key repair proteins [41,42,43]. Following repair, chromatin structure is restored through histone deacetylation and nucleosome reassembly, ensuring genome stability and preservation of epigenetic information [44,45,46].

Cyclin-dependent kinases (CDKs) regulate DNA damage response signaling by coordinating the phosphorylation and recruitment of key repair factors in a cell cycle–dependent manner. Through modulation of proteins such as BRCA1 and 53BP1, CDKs influence DNA repair pathway choice by promoting HR during S/G2 phases while limiting non-homologous end joining (NHEJ) [47,48,49].

CDKs also influence DNA repair indirectly by coordinating ubiquitin-mediated chromatin modifications at damage sites. Ubiquitin ligases such as RNF8, RNF168, and RNF4 promote histone ubiquitination, creating binding platforms for repair factors like BRCA1 and 53BP1 and enabling efficient assembly of DNA damage response complexes [50,51,52] (Fig. 1).

**Figure 1:**
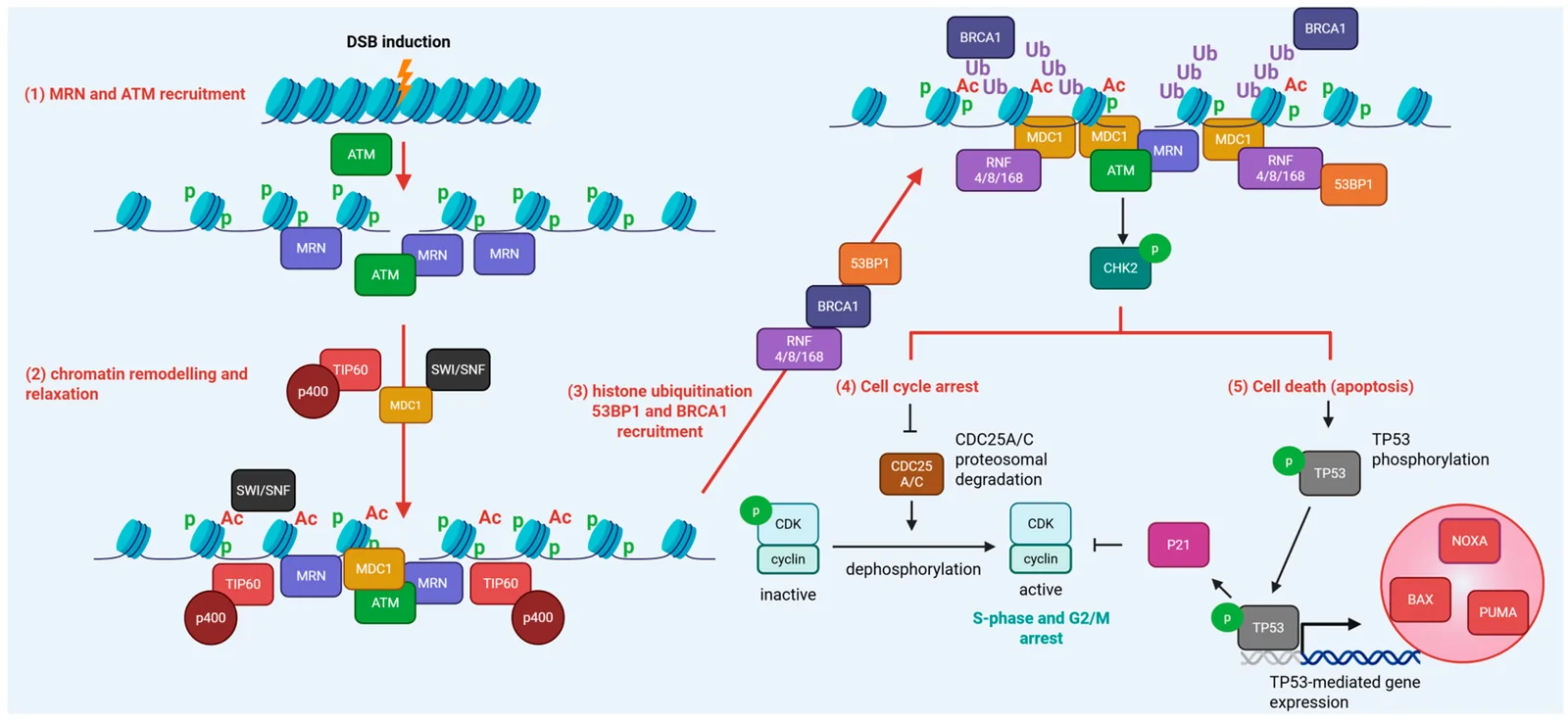
Model illustrating activation of the ATM–CHK2 pathway in response to DSBs and its integration with chromatin remodeling processes in eukaryotic cells. Induction of DSBs triggers rapid and reversible alterations in chromatin architecture that regulate accessibility and propagation of DDR signaling. Upon break formation, MDC1 recognizes and associates with phosphorylated histone H2AX (γH2AX), serving as a platform to potentiate DDR signaling through recruitment of the E3 ubiquitin ligases RNF8, RNF168, and RNF4. These enzymes catalyze ubiquitin conjugation to histone H2A and γH2AX, promoting hierarchical assembly of downstream repair factors, including BRCA1 and 53BP1, thereby influencing repair pathway selection. Because compact nucleosomes initially hinder access to DNA lesions, early DDR events include chromatin decondensation. This is mediated by histone acetyltransferases such as TIP60-p400 and CBP/p300, which acetylate histone tails, together with SWI/SNF remodeling complexes that reposition nucleosomes to enhance factor accessibility. Throughout repair, chromatin remains in an open configuration to permit lesion processing. Subsequent attenuation of ATM–CHK2 signaling is coupled with histone deacetylation and progressive chromatin re-compaction, restoring higher-order structure and safeguarding genomic and epigenetic stability. ATM kinase activates CHK2, which phosphorylates dual-specificity phosphatases CDC25A/C, triggering their degradation, thereby facilitating cell cycle arrest. Alternatively, CHK2 activates TP53 that up-regulates expression of P21, BAX, NOXA, and PUMA. For full names of the proteins see the abbreviations section. Created in BioRender. Kciuk, M. (2026) https://BioRender.com/gfxcllu (accessed on 30 May 2026).

Emerging evidence further extends ATM function to centrosomal regulation, ubiquitin-mediated proteostasis, transcriptional feedback loops, and oncogenic signaling cross-talk, with direct implications for therapeutic targeting. Consistent with this multifaceted role, ATM inhibition displays synthetic lethality in multiple DNA repair-defective tumor contexts and synergizes with poly (ADP-ribose) polymerase (PARP), ataxia telangiectasia and rad3-related (ATR), and cell-cycle checkpoint inhibitors, positioning ATM as both a central genome guardian and a critical vulnerability in precision cancer therapy [53,54,55,56].

Despite substantial progress in understanding ATM signaling and the growing clinical interest in ATM inhibitors, several critical gaps remain. Current knowledge is fragmented across mechanistic studies, preclinical models, and early-phase clinical investigations, with limited integration of how ATM’s context-dependent functions, ranging from tumor suppression to tumor-promoting roles, translate into therapeutic vulnerabilities. In particular, there is a lack of comprehensive synthesis linking molecular mechanisms of ATM activity with the pharmacological properties, clinical development, and therapeutic positioning of distinct ATM inhibitors. Furthermore, emerging challenges such as context-specific synthetic lethality beyond canonical HR deficiency, and mechanisms of resistance to ATM-targeted therapies remain insufficiently consolidated.

In this review, we address these gaps by providing an integrated overview of ATM biology and systematically evaluating the current landscape of small-molecule ATM inhibitors, spanning preclinical and clinical development. We further highlight key determinants of therapeutic response, potential resistance mechanisms, and rational combination strategies. By bridging mechanistic insights with translational and clinical perspectives, this work aims to clarify the therapeutic potential of ATM inhibition and inform future development of precision oncology strategies targeting the DDR.

## 2 Rationale for the Use of ATM Inhibitors in Cancer

### 2.1 Context-Dependent Roles of ATM in Cancer

While ATM functions classically as a canonical tumor suppressor within the DDR, its role in cancer evolution is more accurately interpreted through the framework of cancer evolutionary genetics and non-oncogene addiction. Rather than arising from gain-of-function mutations, the apparent “pro-tumor dependency” on ATM in established malignancies is typically driven by selective retention and functional rewiring of wild-type ATM signaling, rather than mutational activation. In early tumorigenesis, oncogene activation (e.g., MYC, RAS, and cyclin E amplification) induces chronic RS and extensive DNA double-strand breaks, thereby creating a strong selective pressure to maintain residual ATM activity to ensure minimal genomic stability and prevent catastrophic mitotic failure. In this context, ATM becomes co-opted as a buffering kinase required for tumor cell survival under RS, a hallmark of non-oncogene addiction, whereby cancer cells become dependent on stress-response pathways that are dispensable in normal tissues. This dependency is further intensified in genetic backgrounds characterized by *TP53* loss or mutation, in which ATM-mediated checkpoint signaling is uncoupled from apoptosis, allowing damaged cells to persist while still relying on ATM–CHK2 signaling to manage replication-associated DNA lesions. Consequently, ATM activity shifts from a barrier against transformation in premalignant cells to a permissive survival pathway in genetically unstable tumors. Importantly, this does not reflect a gain-of-function alteration of ATM itself, but rather a context-dependent synthetic essentiality emerging from oncogene-driven RS and defective TP53-mediated apoptosis. This concept is consistent with the broader paradigm of DDR rewiring in cancer evolution, where tumor cells exploit residual repair capacity to tolerate genomic instability while continuing proliferation, thereby accelerating clonal diversification and tumor progression. Similar principles have been described in models of replication stress–driven tumorigenesis, where ATM and CHK2 signaling are required to prevent replication catastrophe in MYC- or RAS-driven cancers, reinforcing the idea that DDR components can transition from tumor suppressors to essential survival factors depending on oncogenic context and checkpoint integrity [57,58,59,60].

### 2.2 DDR Hyperactivation and ATM Dependency in Cancer Stem Cells

Emerging evidence suggests that cancer stem cells (CSCs), a subpopulation of tumor cells with stem-like properties, often exhibit constitutively active DDR signaling. This heightened DDR can contribute to resistance against DNA-damaging therapies. Accordingly, targeting key DDR kinases, including ATM, has been proposed as a strategy to overcome such therapy resistance [61,62].

### 2.3 Beyond the DDR: Emerging Tumor-Promoting Roles of ATM

Mechanistically, ATM supports tumor progression not only through canonical DDR signaling but also via non-canonical pathways, including activation of nuclear factor kappa-light-chain-enhancer of activated B cells (NF-κB) to prevent cell death of irradiated cells [63,64,65], induction of epithelial-mesenchymal transition (EMT) to facilitate invasion and metastasis [66], stabilization of HIF1α under hypoxia to promote metabolic adaptation [67], glycolysis enhancement [68] and regulation of the pentose phosphate pathway to maintain redox homeostasis and anabolic growth [69,70]. Furthermore, ATM influences inflammatory and immune signaling within the tumor microenvironment, potentially contributing to immune evasion [67,71,72]. Emerging evidence from radiation-associated basal cell carcinoma (BCC) demonstrates that ATM is not only activated as part of a canonical repair response but is also engaged in broader stress-responsive transcriptional networks that may contribute to long-term tumor development. Notably, ATM expression has been reported to be significantly upregulated in radiation-induced BCC compared with sporadic cases, and to strongly correlate with forkhead box O3a protein (FOXO3a) expression, suggesting coordinated regulation of oxidative stress and DDR pathways. In parallel, activation of the ATM–NF-κB signaling axis, reflected by increased expression of P65 (RELA) and tumor necrosis factor alpha (TNF-α), indicates that persistent inflammatory and senescence-associated secretory phenotype (SASP) signaling may be a key component of radiation-driven tumorigenesis. Such chronic activation of inflammatory pathways may create a tissue microenvironment conducive to genomic instability and malignant transformation. Furthermore, ATM has been implicated in mitochondrial quality control through regulation of PTEN-induced putative kinase 1 (PINK1)-mediated mitophagy, linking nuclear DDR to mitochondrial stress adaptation and reactive oxygen species regulation. Collectively, these findings support a model in which radiation-induced carcinogenesis is driven not by isolated DNA repair defects but by sustained dysregulation of integrated ATM-centered networks involving DDR signaling, NF-κB-mediated inflammation, and mitochondrial homeostasis, thereby contributing to the delayed onset and complex molecular landscape of radiation-associated BCC [73].

### 2.4 Synthetic Lethality and Context-Dependent Vulnerabilities in ATM-Targeted Therapy

These diverse functions provide a strong rationale for targeting ATM in cancer. By competitively joining to ATM’s ATP binding site, ATM inhibitors (ATMi) block its kinase activity, prevent phosphorylation of downstream effectors, and impair DNA repair and checkpoint activation. The therapeutic rationale for targeting ATM also relies in part on the concept of synthetic lethality, which is strictly defined as a genetic interaction in which loss of function of either gene alone is compatible with cell viability, whereas simultaneous loss of both genes results in cell death. In cancer therapy, this definition is often operationalized using pharmacological inhibition; however, it is important to distinguish true genetic synthetic lethality from drug-induced synthetic interactions or pharmacological synergy, as most clinically relevant approaches involve kinase inhibition rather than complete genetic ablation [56,74,75].

In this context, the best-characterized example of bona fide synthetic lethality involving ATM is observed in tumors with HR deficiency, particularly *BRCA1/2*-mutant backgrounds, where ATM inhibition further compromises residual DNA damage signaling and repair capacity, leading to catastrophic genomic instability and cell death [76,77]. In contrast, combinations such as ATM inhibitors with PARP [78,79,80], ATR [76,81,82], and CHK1 [55] inhibitors frequently represent context-dependent synthetic interactions, in which partial pathway inhibition enhances replication stress and DNA damage beyond repair thresholds, but does not always meet the strict genetic definition of lethality across all cellular contexts. Therefore, while ATM–PARP or ATM–ATR combinations are often described as synthetic lethal, they may more accurately be considered synthetic dose-dependent vulnerabilities or pharmacological synergies, depending on the degree of pathway redundancy and residual activity. Importantly, the effectiveness of ATM-targeted strategies must also be interpreted in the context of intra-tumoral genetic heterogeneity. Most solid tumors are composed of genetically diverse subclonal populations, and ATM mutations or functional loss may be restricted to only a subset of cells. Under such conditions, ATM inhibition may induce selective pressure that eliminates ATM-deficient or replication-stressed clones, while ATM-proficient subclones may persist and contribute to therapeutic resistance and disease relapse. Thus, the overall tumor response is determined not only by the presence of ATM deficiency but also by the spatial and temporal distribution of DNA repair defects within the tumor ecosystem, highlighting the need to integrate clonal architecture into the prediction of synthetic lethal responses in ATM-targeted therapies [56,74,75,83].

Beyond canonical HR-deficient contexts, emerging evidence indicates that ATM dependency can also arise from oncogenic and metabolic stress imposed by loss of key tumor suppressors such as phosphatase and tensin homolog (*PTEN*). Mechanistically, *PTEN*-deficient cells exhibit elevated levels of endogenous DNA damage accompanied by constitutive ATM activation, reflecting a heightened reliance on ATM signaling to preserve genome integrity. Although *PTEN* loss has been associated with defective HR in some settings, DNA repair protein RAD51 homolog 1 (RAD51) expression and function remain intact in certain *PTEN*-deficient models, indicating that increased DNA damage arises through alternative mechanisms. In particular, *PTEN* deficiency is tightly linked to enhanced oxidative stress, which promotes DNA base oxidation and accumulation of DSBs, thereby creating a cellular state in which ATM activity becomes essential for survival. Consistent with this model, antioxidant treatment attenuates the cytotoxic effects of ATM inhibition in *PTEN*-deficient cells, underscoring oxidative DNA damage as a key driver of synthetic lethality in this context [84].

### 2.5 ATM Inhibition as a Chemosensitizing and Radiosensitizing Strategy in Cancer Therapy

Even in ATM-proficient tumors, ATMi sensitize cells to DNA-damaging therapies, including IR [85,86], topoisomerase (TOP) inhibitors [85,87], and platinum-based chemotherapies [88,89,90], by preventing DSB repair and forcing damaged cells into mitosis, ultimately triggering cell death.

Importantly, pharmacologic inhibition differs fundamentally from genetic ablation: partial ATM activity is retained, which may mitigate systemic toxicity while preserving anti-tumor efficacy, expanding the therapeutic window [86,91,92]. Clinical and preclinical studies have demonstrated that deletion or inhibition of ATM can potentiate radiotherapy (RT) and chemotherapy across diverse malignancies, including gliomas [93,94], breast [95,96], lung [97], and head and neck cancers [98,99], with particular efficacy in tumors harboring DDR deficiencies. Emerging preclinical evidence further refines key considerations for the clinical implementation of ATM inhibitors, particularly regarding optimal sequencing, duration of exposure, and patient stratification.

Recent studies with AZD1390 demonstrate robust and consistent radiosensitization across diverse breast cancer patient-derived xenograft (PDX) models, irrespective of intrinsic radiosensitivity or receptor status, achieving tumor growth inhibition of up to ~80–85% when combined with radiotherapy. Notably, these effects were accompanied by sustained tumor control in a substantial proportion of cases, supporting the concept that ATM inhibition can broaden the therapeutic benefit of radiotherapy beyond inherently radiosensitive tumors. However, delayed tumor regrowth observed in approximately one-third of models highlights a critical challenge, namely, the emergence of adaptive resistance. Mechanistically, resistance does not appear to arise from restoration of DNA damage repair capacity, but rather from transcriptional reprogramming involving upregulation of oncogenic pathways (e.g., matrix metalloproteinase-9 (MMP-9)) and cytoskeletal and migratory signaling (e.g., p21-activated kinase (PAK) pathway components), alongside modulation of hormone-related pathways. These findings suggest that the long-term efficacy of ATM inhibitor–radiotherapy combinations may depend on rational integration with additional targeted agents aimed at suppressing compensatory survival and metastatic programs. Thus, future strategies should prioritize biomarker-driven combination therapies, potentially incorporating inhibitors of invasion, EMT, or pro-survival signaling [96].

Equally important is the optimization of treatment scheduling. Experimental data indicate that even relatively short durations of ATM inhibition (≥8 h) following irradiation are sufficient to enhance radiosensitivity, with maximal effects observed at ~24 h. This has important translational implications, suggesting that prolonged systemic exposure may not be necessary, thereby potentially reducing toxicity while preserving efficacy. Furthermore, the interaction between ATM inhibition and radiotherapy appears to depend on fractionation schemes, with certain regimens (e.g., hypofractionation versus single high-dose irradiation) demonstrating differential benefit from combination therapy. This underscores the need to tailor ATM inhibitor scheduling to specific radiotherapy protocols rather than adopting a uniform approach. From a personalization perspective, genetic context remains a critical determinant of response. For example, *TP53* deficiency, highly prevalent in several tumor types, has been associated with enhanced sensitivity to ATM inhibition due to impaired cell cycle checkpoint control and increased susceptibility to mitotic catastrophe. Additionally, persistent DNA damage signaling and genomic instability observed in some models suggest that ATM inhibition may induce a state of prolonged vulnerability to subsequent DNA-damaging therapies, opening avenues for adaptive or sequential treatment strategies [96,100].

### 2.6 ATM Inhibition as a Strategy to Enhance Anti-Tumor Immunity

Beyond monotherapy or conventional combination strategies, recent evidence suggests that ATMi can enhance immunogenicity by promoting cyclic GMP-AMP synthase–stimulator of interferon genes (cGAS–STING) pathway activation and programmed death-ligand 1 (PD-L1) upregulation, creating a compelling rationale for novel radio-immunotherapy or chemo-immunotherapy regimens [101,102].

## 3 ATM Inhibitors in Cancer Treatment

### 3.1 Completed Studies

#### 3.1.1 Early ATM Inhibitors

Early pharmacological efforts to modulate the DDR and enhance radiosensitivity relied largely on nonspecific agents, laying the foundation for the development of more selective ATM inhibitors. Among the earliest compounds, wortmannin, a fungal metabolite originally characterized as a phosphoinositide 3-kinase (PI3K) inhibitor, was the first small molecule proposed to target ATM. Wortmannin irreversibly inhibits multiple members of the PIKK family, including ATM, DNA-PKcs, and mechanistic target of rapamycin (mTOR) at nanomolar concentrations, with weaker activity against ATR at micromolar levels. Moreover, the covalent and irreversible binding nature of wortmannin results in significant systemic toxicity and adverse effects *in vivo*, severely limiting its translational utility. Notably, renewed interest in wortmannin emerged with the development of nanoparticle-based delivery systems, which markedly reduced toxicity and reopened the possibility of exploiting its DDR-modulating properties in a therapeutic context [103,104,105].

Caffeine represents another historically important radiosensitizer that has been extensively studied, although its precise molecular mechanism remains incompletely defined. Structurally related to methylxanthines, caffeine acts as a weak and broad-spectrum kinase inhibitor at high concentrations, and was later shown to inhibit ATM and ATR activities at doses comparable to those required for radiosensitization. However, the concentrations necessary to inhibit ATM (IC_50_ ≈ 0.2 mM) approach or exceed toxic thresholds *in vivo*, precluding its clinical application and underscoring the need for more potent and selective agents [106,107,108,109].

These limitations ultimately drove the rational development of specific ATM inhibitors. Building on early screening efforts, Smith and colleagues identified the chromenone derivative LY294002 [110] as a starting scaffold, which was subsequently optimized to improve potency and selectivity. This work culminated in the generation of KU-55933, the first highly selective ATP-competitive inhibitor of ATM. Structural modifications, including replacement of the chromen-4-one core with a pyran-4-one and incorporation of a thianthrene moiety, yielded a compound with nanomolar potency against ATM and strong selectivity over related kinases such as ATR, DNA-PKcs, PI3K, and mTOR [111]. The development of KU-55933 marked a pivotal advance in the field, providing a pharmacological tool capable of selectively dissecting ATM function and establishing proof-of-concept for ATM inhibition as a viable strategy for radiosensitization leading to the development of KU-59403 and KU-60019 (Fig. 2) [112].

**Figure 2:**
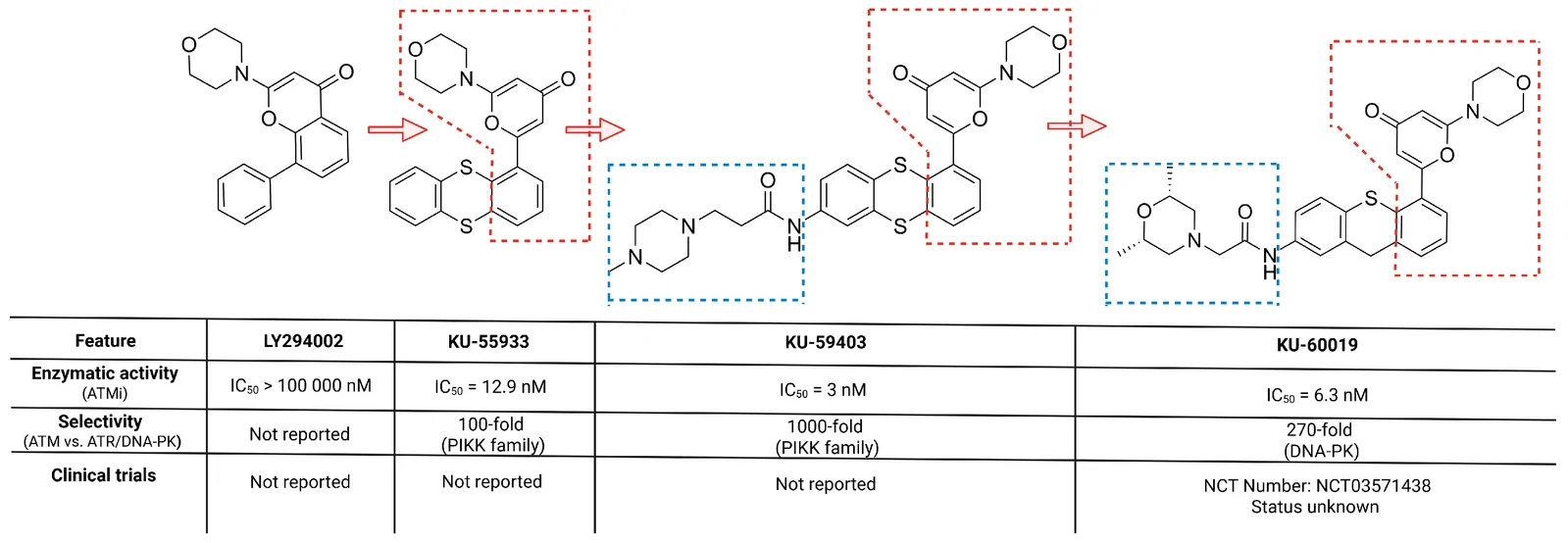
Hit-to-lead optimization from LY294002 to KU-60019 with compounds properties [112]. Created in BioRender. Kciuk M (2026) https://BioRender.com/2jhkkx9 (accessed on 11 May 2026).

Subsequent efforts focused on improving pharmacokinetic properties, cellular permeability, and *in vivo* tolerability, which led to the development of several additional ATM inhibitors with diverse chemical scaffolds. One of the early compounds emerging from these efforts was CP466722. Further medicinal chemistry optimization led to additional small-molecule inhibitors with improved potency and cellular activity, including WSD0628, which displayed enhanced inhibitory activity against ATM and showed promising radiosensitizing effects in preclinical tumor models. Parallel drug discovery programs also produced compounds with improved pharmacological properties suitable for clinical development. Among these, AZD0156 and AZD1390, developed by AstraZeneca, represent highly potent and selective ATP-competitive ATM inhibitors. AZD0156 demonstrated strong activity in combination with DNA-damaging agents and radiation, while AZD1390 was specifically designed to achieve ATM inhibition in central nervous system malignancies [113,114,115] (Fig. 3) as discussed in subsequent sections.

**Figure 3:**
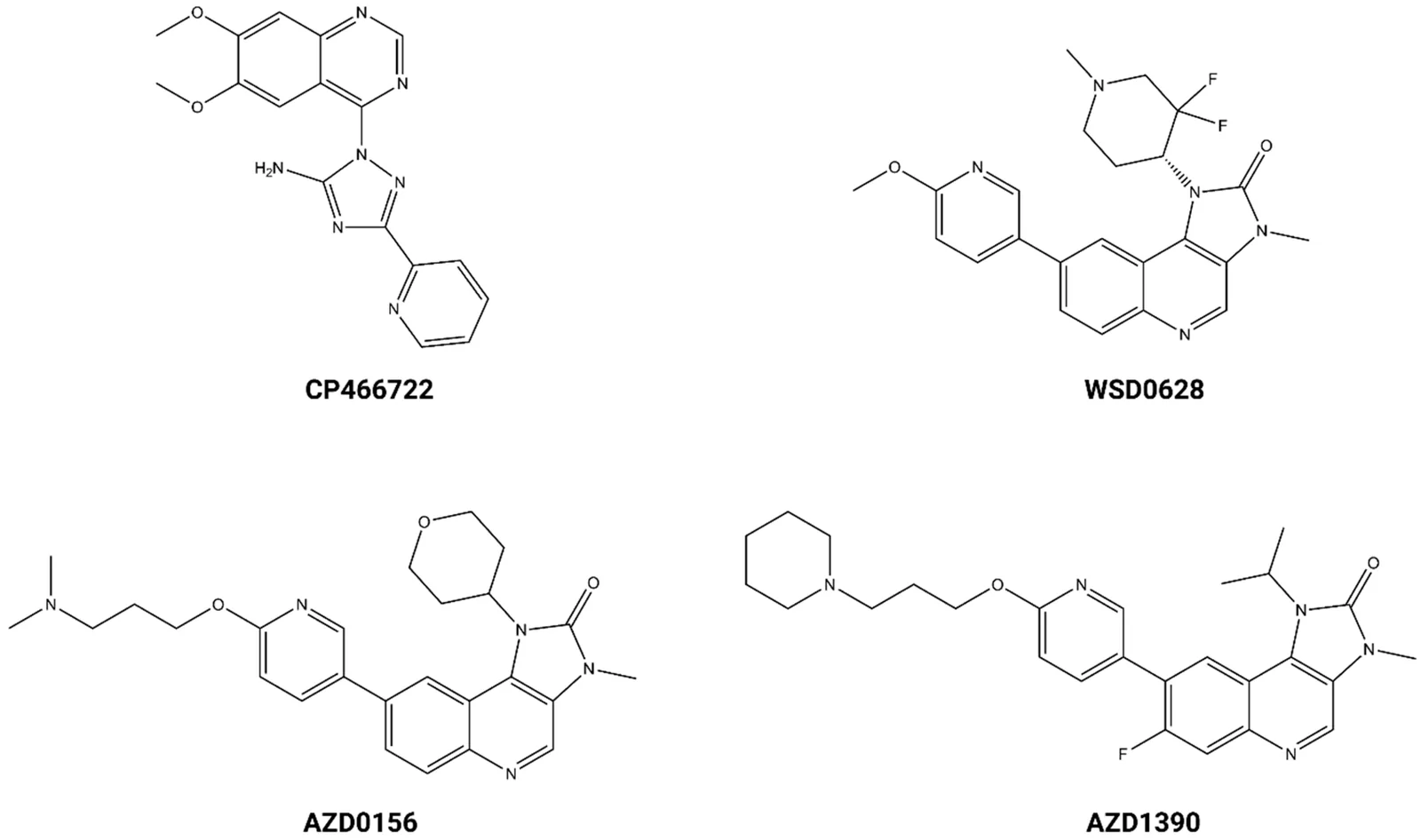
Chemical structures of CP466722, WSD0628, AZD0156, and AZD1390.

#### 3.1.2 KU-55933

KU-55933 is a seminal ATP-competitive inhibitor that has fundamentally shaped the understanding of ATM biology and its therapeutic exploitation in cancer. KU-55933 potently inhibits ATM with nanomolar activity (IC_50_ ≈ 13 nM; K_i_ ≈ 2.2 nM) and high selectivity over related kinases such as ATR and DNA-PKcs. In cellular systems, KU-55933 selectively abrogates IR–induced phosphorylation of canonical ATM substrates, including TP53, γH2AX, Nibrin (NBS1), and structural maintenance of chromosomes protein (SMC1), while sparing UV-induced ATR signaling, thereby phenocopying genetic *ATM* deficiency. Functionally, ATM inhibition by KU-55933 robustly sensitizes cancer cells to IR and DSB–inducing chemotherapeutics such as etoposide, doxorubicin, and camptothecin (CPT), with no additional effects in *ATM*-null AT cells, confirming its on-target specificity [111]. Comparative analyses with DNA-PKcs inhibitors demonstrated complementary DDR roles, wherein ATM governs early γH2AX signaling intensity, and DNA-PKcs controls later repair kinetics. Furthermore, combined inhibition profoundly suppresses H2AX phosphorylation and markedly enhances radiosensitization in breast cancer models [116].

KU-55933 was also pivotal in defining ATM as a critical regulator of HR, reducing its efficiency by up to 90% in growth-arrested cells and revealing crosstalk between ATM and rapidly accelerated fibrosarcoma (RAF)/mitogen-activated protein kinase kinase (MEK)/extracellular signal-regulated kinase (ERK) signaling pathways that influence genome stability and therapy response [117]. Synthetic lethal interactions have further underscored its translational relevance: Fanconi anemia pathway–deficient fibroblasts and pancreatic cancer cells exhibit constitutive ATM activation and exquisite sensitivity to KU-55933, resulting in DNA breakage, checkpoint failure, and apoptosis [118]. KU-55933 has also exposed non-canonical ATM functions, including regulation of ribonucleotide reductase (RNR), mitochondrial homeostasis, and cellular metabolism, where ATM inhibition induced mitochondrial DNA depletion and metabolic stress [119]. Mechanistic dissection of PIKK signaling using KU-55933 clarified pathway specificity during RS and response to platinum chemotherapy, demonstrating that ATM, ATR, and DNA-PKcs distinctly shape RPA2 phosphorylation, CHK1 signaling, and drug sensitivity [120]. Subsequent studies expanded ATM’s role into S-phase checkpoint control and RS responses, showing that KU-55933 phenocopies Werner syndrome protein (*WRN*) deficiency during interstrand crosslink repair [121], modulates tumor immunogenicity by suppressing ATM/ATR-dependent induction of natural killer (NK)-activating ligands in multiple myeloma [122], and blocks ATM-dependent MEK/ERK/NF-κB prosurvival signaling during radioadaptive responses [123].

In melanoma, KU-55933 sensitizes cells to TNF-related apoptosis-inducing ligand (TRAIL)-induced apoptosis by inhibiting ATM-signal transducer and activator of transcription 3 (STAT3) signaling, downregulating (FADD-like IL-1β–converting enzyme)-inhibitory protein (cFLIP), and upregulating death receptor 5 (DR5) independently of TP53 and NF-κB [124]. ATM-dependent control of nucleotide metabolism was elucidated when KU-55933 was shown to block CHK1- and E2F transcription factor 1 (E2F1)-mediated induction of ribonucleotide reductase regulatory subunit M2 (RRM2) following CPT treatment, thereby impairing deoxyribonucleoside triphosphate (dNTP) synthesis and enhancing DNA damage [125]. Cytoplasmic ATM signaling has likewise been implicated in protein kinase B (PKB/AKT) activation and survival. KU-55933 suppresses insulin- and insulin-like growth factor 1 (IGF-1)–induced AKT phosphorylation, induces G1 arrest via cyclin D1 downregulation, promotes apoptosis, and synergizes with rapamycin by preventing compensatory AKT activation [126]. ATM inhibition also alters core cell-cycle and apoptotic regulators across cancer models, affecting cyclins, retinoblastoma protein (pRB) phosphorylation, TP53 activation, and Bcl-2-associated X protein (BAX) or P21 expression [127]. Interactions between ATM and AKT further influence cell-cycle-phase–specific DNA repair capacity and radioresistance [128].

KU-55933 has been extensively employed to delineate ATM-dependent responses under oxidative stress and in various chemotherapeutic contexts. Studies have demonstrated its involvement in selenium-induced DNA damage responses [129], doxorubicin-mediated checkpoint regulation [130], cadmium-induced radioadaptive responses [131], sustained P21-dependent G2 cell cycle arrest [132], ROS-driven apoptosis [133], and the protection of normal ovarian tissue from chemotherapy-induced apoptosis [134].

Importantly, KU-55933 selectively radiosensitizes glioma stem cells while sparing differentiated tumor cells, implicating ATM as a key determinant of CSC radioresistance [135]. ATM kinase activity also feeds back on its own transcriptional regulation through BRCA1, E2F1, and C-terminal binding protein (CtBP) interacting protein (CtIP), as revealed by inhibition studies involving KU-55933 [136].

In glioblastoma multiforme (GBM), ATM inhibition with KU-55933 markedly sensitizes O^6^-methylguanine-DNA methyltransferase (*MGMT*)-negative tumors to temozolomide by impairing DSB repair and sustaining γH2AX signaling, while providing little benefit in *MGMT*-overexpressing resistant models [137].

Oncogenic kinase-driven resistance, such as that mediated by Aurora-A, can be reversed by the selective ATM inhibition with KU-55933 [138]. Similarly, KU-55933 blocks ATM-dependent autophagy mediated via the AMPK-Unc-51-like autophagy activating kinase 1 (ULK1) pathway, thereby enhancing temozolomide cytotoxicity [139]. In lung cancer, ATM-driven cytoprotective autophagy limits cisplatin-induced radiosensitization, an effect that is also reversed by KU-55933 [140].

Across multiple tumor types, including GBM, lung, breast, bladder, gynecologic, and hepatocellular cancers, KU-55933 consistently enhances the efficacy of RT, chemotherapy, and targeted agents with particularly strong effects in therapy-resistant and stem-like populations [141,142,143]. Context-dependent effects have also been noted, as inappropriate dual PIKK inhibition may paradoxically increase survival under specific genotoxic exposures [144], highlighting the need for rational scheduling and patient selection.

Advances in nanoparticle and mitochondria-targeted delivery of KU-55933 have improved *in vivo* radiosensitization while reducing normal tissue toxicity [145,146]. Recent studies have expanded the role of ATM into metabolic reprogramming and tumor invasiveness, demonstrating that ATM promotes glucose transporter 1 (GLUT1) trafficking, glycolysis, EMT, and metastatic potential in AKT-hyperactivated tumors. These processes are disrupted by KU-55933 and are further sensitized by mitochondrial stressors [147,148]. KU-55933 also reverses multidrug resistance by inhibiting ATP-binding cassette subfamily G member 2 (ABCG2) efflux activity [149] and exhibits synergistic effects with PARP inhibitors [150], epidermal growth factor receptor (EGFR) tyrosine kinase inhibitors [151], thioredoxin reductase inhibitors [152], and hypoxia-targeted strategies [153] in genetically defined contexts. However, the clinical utility of KU-55933 is constrained by its poor aqueous solubility and limited *in vivo* bioavailability (Table S1).

#### 3.1.3 KU-59403

In preclinical studies, KU-59403 exhibited sufficient potency, selectivity, and solubility to enable advanced pharmacologic evaluation, yet showed minimal intrinsic cytotoxicity as a single agent across multiple human cancer cell lines, including colorectal and breast cancer models. Instead, its primary antitumor activity emerged in combination settings, where KU-59403 significantly potentiated the cytotoxic effects of TOP I and II poisons, including CPT, etoposide, and doxorubicin, as well as IR, through suppression of ATM-dependent DNA damage signaling and repair. Notably, this chemosensitization and radiosensitization were independent of *TP53* status, indicating broad applicability across genetically heterogeneous tumors. Pharmacokinetic (PK) studies in mice demonstrated that KU-59403 achieved favorable tissue distribution, with tumor concentrations exceeding those required for *in vitro* ATM inhibition, sustaining for several hours following systemic administration. *In vivo* efficacy studies further showed that KU-59403 markedly enhanced the antitumor activity of TOP inhibitors in human colon cancer xenograft models at doses that were non-toxic as monotherapy and well tolerated in combination regimens, with chemosensitization dependent on both dose and treatment schedule. Despite these encouraging findings, published data on KU-59403 remain limited to a small number of foundational preclinical studies, and no subsequent clinical development or extensive mechanistic exploration has been reported. As such, KU-59403 occupies an important but largely historical position in the ATM inhibitor field, serving as one of the first compounds to demonstrate that systemic ATM inhibition can be pharmacologically feasible, well tolerated, and therapeutically synergistic *in vivo*, thereby paving the way for later generations of more potent and clinically advanced ATM inhibitors [154].

#### 3.1.4 KU-60019

KU-60019 is a second-generation ATM kinase inhibitor derived from KU-55933 that has been extensively validated as a potent radiosensitizer and antitumor agent across multiple cancer models [155]. In GBM, KU-60019 produces rapid, reversible, and near-complete suppression of the DDR at nanomolar concentrations, inhibiting phosphorylation of canonical ATM substrates including TP53, H2AX, KRAB-associated protein 1 (KAP1), and AKT, while effectively radiosensitizing both bulk tumor cells and glioma-initiating/stem-like cells [135,156]. Radiosensitization by KU-60019 is highly genotype-dependent, with greatest efficacy observed in tumors harboring mutant or low *TP53* expression and elevated PI3K/AKT signaling [157,158,159]. *In vivo* studies using orthotopic GBM xenografts demonstrated that local delivery of KU-60019 in combination with radiation significantly prolongs survival, particularly in *TP53*-mutant tumors, without detectable neurotoxicity [157]. Beyond glioma, KU-60019 exploits synthetic lethal interactions in *PTEN*-deficient cancers [160,161], where ATM addiction driven by oxidative DNA damage renders tumor cells highly sensitive to ATM inhibition alone or in combination with radiation, cisplatin [88], PARP inhibitors [162], or TOP II poisons [87].

Mechanistically, KU-60019 disrupts DNA repair, abrogates the G2 checkpoint, suppresses pro-survival AKT signaling, interferes with autophagic flux, and enhances apoptotic cell death [155,163]. More recent studies further suggest that KU-60019 may modulate tumor immune evasion by downregulating *PD-L1* and that its therapeutic index can be improved by combination with redox-modulating agents such as pharmacologic ascorbate, which enhances tumor radiosensitization while protecting normal tissues [164,165].

In SKOV3 ovarian cancer cells, treatment with KU-60019 for 24 h resulted in a significant downregulation of hsa-miR-1273g-3p accompanied by an elevation of diacylglycerol O-acyltransferase 1 (*DGAT1*) expression, as determined by RT-qPCR. Functional enrichment analyses revealed that *DGAT1*-associated genes were involved in cytokine receptor interaction, T-cell receptor signaling, and apoptotic pathways, suggesting a broader role of DGAT1 in tumor-related immune and survival processes. Mechanistically, overexpression of hsa-miR-1273g-3p via synthetic mimics abrogated the KU-60019-induced upregulation of DGAT1 and partially restored cell viability, supporting a direct regulatory interaction between miR-1273g-3p and DGAT1 in the context of ATM inhibition. Moreover, pharmacological inhibition of DGAT1 counteracted KU-60019-mediated effects on cancer cells’ migration, further supporting the functional importance of this axis. Clinically, Kaplan–Meier survival analysis revealed that elevated *DGAT1* expression was associated with poorer overall survival in ovarian cancer patients [166] (Table 1).

**Table 1:** Key preclinical insights derived from studies involving use of KU-60019 as ATM inhibitor.

Model/System	Key Findings	PMID
Human malignant glioma cells (U87, U1242, U1242/luc-GFP), normal human fibroblasts (GM02270), and A-T fibroblasts (GM05823)	KU-60019, an improved KU-55933 analogue, showed enhanced ATM inhibition and radiosensitization of glioma cells, with reduced AKT phosphorylation, migration, and invasion via suppression of AKT/MEK-ERK signaling. Additional MEK/AKT inhibition did not further increase radiosensitization.	19808981
Human glioblastoma cell lines (U1242, U1242/luc-GFP, U373, U87) and hESC-derived human astrocytes	KU-60019 induced rapid, reversible ATM/DDR suppression in GBM cells, inhibited phosphorylation of ATM targets and AKT phosphorylation, enhanced radiosensitivity (more than TMZ), and reduced glioma growth with minimal toxicity to normal astrocytes.	22370485
Patient-derived glioma stem cell (GSC) lines: BORRU (strong stem phenotype) and DR177 (weak stem phenotype), derived from grade IV GBM	ATM-driven DDR was constitutively active in GSCs. KU-60019 selectively radiosensitized stem-like glioma cells to DSB-inducing agents via ATM inhibition and G2/M accumulation, while sparing differentiated/nonstem cells.	22257080
Orthotopic GBM xenograft models in mice using human glioma cell lines expressing luciferase/fluorescent reporters: U1242/luc-GFP (p53 R175H), U87/luc-DsRed (p53 WT), and isogenic U87/luc-DsRed-p53 (281G);	KU-60019 inhibited ATM/DDR signaling *in vivo* and markedly enhanced radiosensitivity and survival in glioma-bearing mice, with strongest effects in *TP53*-mutant tumors consistent with synthetic lethality.	23620409
Primary human glioblastoma-initiating cell (GIC) lines derived from Grade IV GBM (2.11, BORRU, COMI, DEMI, DR177, MPM176, PERU, VIPI), all capable of forming orthotopic tumors in NOD/SCID mice	KU-60019 selectively radiosensitized proliferative GICs in a *TP53*-low/*PI3K*-high and ATM-dependent manner, eradicated radioresistant populations without inducing stable resistance, and showed minimal toxicity or mutagenicity *in vitro* and *in vivo*.	24443327
Adult and pediatric glioma-initiating cells (GICs) capable of forming orthotopic tumors in NOD/SCID mice; adult GB GICs with varying TP53/PI3K expression profiles and pediatric high-grade glioma GICs (WHO grade III and IV); KU-60019 delivered intracranially by convection-enhanced delivery (CED) with pharmacokinetic analysis by HPLC-MS; orthotopic GB models and *in vitro* proliferation/radiosensitization assays	KU-60019 required local intracranial delivery due to poor BBB penetration, showed rapid brain clearance with minimal toxicity, and radiosensitized adult and pediatric GICs according to *TP53*/*PI3K* status.	25091220
Orthotopic human glioblastoma xenografts in mice generated from U87 and U1242 glioma cell lines, including isogenic p53 wild-type and mutant (p53-281G) variants	KU-60019 inhibited ATM/DDR signaling *in vivo* and enhanced glioma radiosensitivity and survival, with strongest effects in *TP53*-mutant and encapsulated tumors, while reducing invasion and showing minimal normal-brain toxicity.	23620409
PTEN-deficient and PTEN-proficient human cancer cell lines (including HCT116 PTEN-null cells, PC3 cells with inducible PTEN re-expression, ATM-deficient and ATM-reconstituted human fibroblasts), complemented by tumor xenograft models	*PTEN* loss induced ROS-dependent DNA damage and constitutive ATM activation, creating synthetic lethality with ATM inhibition. KU-60019 selectively eliminated *PTEN*-deficient tumors by triggering catastrophic genomic instability and apoptosis.	25870146
Human breast cancer cell lines including PTEN-deficient MDA-MB-468 (triple-negative breast cancer, TNBC) and PTEN–wild-type MDA-MB-231, MCF-7, and T47D cells	KU-60019 selectively enhanced cisplatin cytotoxicity in *PTEN*-deficient breast cancer cells via synthetic lethality, increasing apoptosis by exacerbating DNA damage and impairing ATM-dependent repair and HR.	29522753
Human cancer cell lines including nasopharyngeal carcinoma (NPC) lines (C666-1, CNE2, HNE1, HONE1), HeLa, H1299, and telomerase-immortalized normal nasopharyngeal epithelial cells	ATM inhibition increased DNA damage and compensatory PARP activity; combined *ATM* and *PARP1* loss caused synergistic DNA damage accumulation and G2 checkpoint activation, indicating bidirectional synthetic lethality driven mainly by impaired DNA repair rather than PARP trapping. Bypass of the G2 checkpoint in *ATM*-deficient cells occurred only after a delay, indicating that checkpoint adaptation rather than immediate failure contributes to cell death.	31597711
Human and murine NSCLC cells (BEAS-2B, HEK293T, CRISPR-engineered PTEN-mutant clones, KP and KPP lines), organotypic *ex vivo* tumor slices	*PTEN* loss drives a pro-resistance transcriptional program and ATM-dependent DNA damage repair reliance. ATM inhibition alone had limited activity but strongly radiosensitized *PTEN*-deficient tumors in combination with IR, an effect reversible by *PTEN* restoration.	35477555
Human lung cancer cells (H1299, A549) and HEK293 normal cells	KU-60019 interfered with ATM–TOP2β interaction, stabilizing TOP2β-DNA cleavage complexes. This impaired repair of VP-16-induced DSBs led to increased DNA damage accumulation and apoptosis. KU-60019 combined with VP-16 synergistically suppressed cell growth and survival. Effects were dose- and time-dependent and observed in multiple lung cancer cell lines.	36650267, 38627539, 37620619
Human tumor cell lines (HT-1080 fibrosarcoma, H-460 lung cancer)	KU-60019, alone or combined with vacquinol-1, showed synergistic *PD-L1* inhibition. System biology analysis highlighted potential kinase pathways regulating *PD-L1* expression.	31626963
Human colorectal tumor cells (HCT116, SW480, HT29) and murine colorectal tumor cells (CT26, MC38); normal cells: HUVEC, FHs74	KU-60019 combined with pharmacologic ascorbate enhanced tumor radiosensitization via H_2_O_2_-mediated oxidative DNA damage and G2 checkpoint abrogation, while reducing normal tissue toxicity and fibrosis and inhibiting ATM activation in tumors.	36228747

#### 3.1.5 CP466722

CP466722 represents one of the earliest pharmacological probes developed to interrogate and therapeutically exploit ATM kinase function in DDR. Identified through targeted compound library screening, CP466722 is a selective, cell-active ATM inhibitor that suppresses ATM-dependent phosphorylation events without inhibiting PI3K or other PI3K-like kinases. A distinctive feature of CP466722 is the rapid and fully reversible inhibition of ATM activity upon drug withdrawal, enabling temporal dissection of ATM signaling that is not achievable with genetic ablation approaches. Using this compound, transient ATM inhibition was shown to be sufficient to abrogate cell cycle checkpoints and robustly sensitize tumor cells to IR, demonstrating that short-lived ATM suppression can confer durable radiosensitization [86]. These properties positioned CP466722, alongside KU-55933, as a foundational preclinical tool supporting ATM as a rational radiosensitization target [167]. Beyond RT, CP466722 has been instrumental in defining synthetic lethal interactions between ATM and other DNA repair pathways. Concurrent inhibition of ATM and PARP using CP466722 and AZD2461 enhanced apoptosis and growth suppression in breast cancer models, underscoring the dependence of PARP-inhibited cells on intact ATM-mediated damage signaling [168]. However, subsequent chemical proteomic profiling revealed that CP466722 also binds strongly to activin receptor-like kinase 2 (ALK2), highlighting unanticipated off-target activity and emphasizing the importance of orthogonal validation when interpreting biological effects attributed to ATM inhibition [169]. Despite these limitations, CP466722 continues to be leveraged in translational and computational studies, including drug sensitivity modeling in diffuse large B-cell lymphoma and ovarian cancer, where predicted responsiveness to CP466722 correlates with specific epigenetic or relapse-associated risk signatures [170,171]. CP466722 has also provided important insights into the non-canonical functions of ATM in chemotherapy resistance and tumor progression. In models of cisplatin-resistant lung cancer, *ATM* expression is markedly upregulated following chronic cisplatin exposure and is further inducible by cisplatin treatment in parental cells, coinciding with the acquisition of aggressive phenotypes characterized by EMT, enhanced migration, invasion, and metastatic capacity. Pharmacological inhibition of ATM using CP466722, or genetic depletion of *ATM*, effectively reverses EMT markers, restoring E-cadherin while suppressing N-cadherin, vimentin, and snail family transcriptional repressor 1 (SNAIL), and significantly attenuates invasive and metastatic behavior both *in vitro* and in orthotopic xenograft models. Mechanistically, ATM was shown to promote immune-evasive and pro-metastatic signaling by upregulating *PD-L1* expression through activation of the JAK1/2–STAT3 pathway, linking DNA damage signaling directly to immune checkpoint regulation. Inhibition of ATM with CP466722 suppresses JAK/STAT3 signaling, reduces *PD-L1* expression, and phenocopies the effects of PD-L1 neutralization or JAK/STAT inhibition in limiting EMT and invasion [66] (Table 2).

**Table 2:** Key preclinical insights derived from studies involving use of CP466722 as ATM inhibitor.

Model/System	Key Findings	PMID
Human cancer and normal cell lines (HeLa, MCF7, HFF, hTERT-immortalized HFF, A-T fibroblasts), mouse embryonic fibroblasts (ATM wild-type and ATM-deficient), and murine pre-B cells expressing BCR-ABL	CP466722 is a selective, reversible, and non-toxic ATM inhibitor that suppressed ATM signaling, induced checkpoint defects, and radiosensitized *ATM*-proficient cells without affecting *ATM*-deficient cells.	18794134
Human breast cancer cell lines: MCF-7 and SKBr-3	Combined ATM (CP466722) and PARP-1 (AZD2461) inhibition induced synthetic lethality in breast cancer cells, enhancing cytotoxicity, apoptosis, and growth suppression beyond either agent alone.	25337581
Ovarian cancer relapse model: TCGA and GEO patient cohorts; single-cell RNA-seq of primary vs. relapse OC; *in vitro* validation in IOSE-80 and SK-OV-3 cells	Relapse OC showed altered cell composition and a 5-gene RiskScore (LDHA, NOP58, NMU, KRT19, RPS23) predicting poor prognosis and broad chemoresistance (including CP466722), while *KRT19* knockdown reduced invasion and migration *in vitro*.	40821986
Cisplatin-resistant NSCLC models: A549CisR and H157CisR cells derived from parental A549P and H157P cells; orthotopic xenograft mouse model	Cisplatin-resistant lung cancer cells exhibited EMT, increased ATM expression, and enhanced invasiveness, while ATM inhibition (CP466722 or knockdown) reversed EMT, reduced metastasis, and lowered *PD-L1* expression *in vitro* and *in vivo*.	30961670

#### 3.1.6 WSD0628

WSD0628 is a next-generation, small-molecule inhibitor of ATM kinase that was rationally developed to overcome the limited CNS exposure of earlier ATM inhibitors and thereby enabling effective radiosensitization of primary and metastatic brain tumors. Preclinical PK studies in mice demonstrated that WSD0628 is orally bioavailable and achieves greater-than-dose-proportional systemic and brain exposure, consistent with saturation of clearance mechanisms, while exhibiting favorable CNS penetration with unbound brain-to-plasma partition coefficients ranging from 0.15 to 0.3 and uniform distribution across brain regions. Importantly, CNS exposure was not restricted by the efflux transporters P-glycoprotein (P-GP) or breast cancer resistance protein (BCRP), establishing WSD0628 as a bona fide brain-penetrant ATM inhibitor suitable for systemic administration [172].

In line with its intended mechanism, WSD0628 displays high kinase selectivity, minimal aldehyde oxidase liability, and low predicted human ether-a-go-go-related gene (hERG) risk, and effectively blocks radiation-induced ATM signaling suppressing γH2AX foci formation [100]. Functionally, WSD0628 potently radiosensitized glioma cells and patient-derived xenograft models of GBM and brain metastases, resulting in prolongation of animal survival in orthotopic models when combined with RT, with median survival increases exceeding an order of magnitude in several models compared with radiation alone [100].

However, consistent with the central role of ATM in normal tissue DDR, WSD0628 also profoundly enhanced radiation-induced acute oral and esophageal toxicities *in vivo*, with sensitizer enhancement ratios increasing with both drug dose and radiation fractionation, effectively abrogating the normal tissue sparing typically conferred by fractionated RT [173].

Collectively, these findings establish WSD0628 as a clinically promising, brain-penetrant ATM inhibitor with exceptional radiosensitizing efficacy in CNS tumors, while simultaneously underscoring the need for carefully optimized radiation strategies to safely translate ATM inhibition into the clinical management of GBM and brain metastases [100,172,173] (Table 3).

**Table 3:** Key preclinical insights derived from studies involving use of WSD0628 as ATM inhibitor.

Model/System	Key Findings	PMID
Preclinical pharmacokinetic and CNS distribution model: Mouse systemic PK studies with oral and intravenous dosing; CNS, plasma, and tissue distribution analysis; P-gp/Bcrp knockout mice; intracranial tumor–bearing mice	WSD0628 is an orally bioavailable ATM inhibitor with consistent CNS penetration, saturable clearance, and predicted clinically achievable brain exposure sufficient for sustained radiosensitization without efflux transporter limitation.	38858089
Preclinical *in vitro* and *in vivo* radiosensitization model: GBM cell lines (U251), brain metastasis and GBM patient-derived xenografts (GBM12, GBM43, GBM120, M12), including orthotopic intracranial PDX models	WSD0628 is a selective, brain-penetrant ATM inhibitor that suppresses radiation-induced ATM signaling, enhances GBM radiosensitivity, and prolongs survival in PDX models when combined with radiation, with confirmed tumor target inhibition.	40230030
Preclinical normal tissue toxicity model: Non–tumor-bearing FVB mice receiving single or fractionated radiation to the oral cavity or esophagus in combination with the ATM inhibitor WSD0628	WSD0628 markedly increased radiation-induced normal tissue toxicity in a dose- and fraction-dependent manner, reducing the sparing effect of fractionation and necessitating modified BED-based radiotherapy scheduling.	39559836

#### 3.1.7 AZD0156

AZD0156 is a potent, selective, and orally bioavailable ATM inhibitor. Extensive preclinical evidence demonstrates that pharmacologic ATM inhibition by AZD0156 effectively abrogates irradiation-induced ATM signaling at low nanomolar concentrations, suppressing phosphorylation of canonical ATM substrates and impairing DSB repair, thereby conferring robust radiosensitization across multiple tumor types while relatively sparing normal tissues [174,175,176,177,178]. AZD0156 has been shown to synergize strongly with IR *in vitro* and *in vivo*, enhancing clonogenic cell death, inducing G2/M checkpoint failure, promoting senescence, and increasing residual DNA damage following fractionated and single-dose RT (effects that are often accentuated in *TP53*-deficient or HR–proficient tumors) [176,177,179]. Mechanistically, ATM inhibition rewires DNA repair pathway choice by suppressing end resection and HR, thereby forcing reliance on error-prone NHEJ, particularly in the context of high linear energy transfer radiation such as proton Bragg peak irradiation, where AZD0156 uniquely enhances relative biological effectiveness compared with ATR inhibition [180].

Beyond RT, AZD0156 exploits synthetic lethal interactions with diverse DDR deficiencies and cytotoxic therapies. ATM inhibition markedly potentiates the activity of PARP inhibitors such as olaparib by preventing repair of replication-associated DNA damage, leading to accumulation of DSBs, sustained checkpoint activation via ATR-CHK1-WEE1 signaling, and apoptotic cell death across lung, gastric, breast, pancreatic, and colorectal cancer models [162,174].

Similarly, AZD0156 demonstrates selective efficacy in *ATM*-deficient or DDR-altered contexts in pancreatic ductal adenocarcinoma (PDAC), biliary tract cancer, lung cancer with transmembrane protein 176A (*TMEM176A*) methylation, esophageal squamous cell carcinoma with silenced schlafen family member 11 (*SLFN11*) silencing, alpha thalassemia/mental retardation syndrome X-linked (*ATRX*)-deficient gliomas, mature T-cell lymphomas, uterine leiomyosarcoma with HR defects, and polo-like kinase 4 (PLK4)-driven chromosomal instability, highlighting ATM dependency as a predictive biomarker of response [181,182,183,184,185].

Importantly, emerging data indicate that AZD0156 exerts profound immunomodulatory effects when combined with DNA-damaging therapies. In pancreatic cancer and head and neck squamous cell carcinoma, ATM inhibition amplifies radiation-induced type I interferon (IFN) signaling through STING-dependent and RNA Polymerase III (POLIII)/retinoic acid-inducible gene I (RIG-I)/mitochondrial antiviral signaling protein (MAVS) pathways, leading to increased expression of IFN-β, major histocompatibility complex class I (MHC-I), enhanced CD8^+^ T-cell infiltration, immunogenic memory formation, and sensitization to immune checkpoint blockade [186,187,188]. However, context-dependent immune effects have also been observed, including immune-suppressive transcriptomic changes and altered cytokine secretion profiles following ATM inhibition in certain settings, underscoring the importance of rational combination strategies and dose optimization [98,189] (Table 4).

**Table 4:** Key preclinical insights derived from studies involving use of AZD0156 as ATM inhibitor.

Model/System	Key Findings	PMID
Preclinical *in vitro* and *in vivo* models: ATM-isogenic FaDu cells; lung, gastric, and breast cancer cell lines; lung xenograft model; two patient-derived triple-negative breast cancer (TNBC) xenograft models	AZD0156 inhibited ATM signaling and acted as a potent radiosensitizer, enhancing radiation and olaparib-induced DNA damage, apoptosis, and tumor growth inhibition across multiple cancer models including TNBC PDX.	31534013
Preclinical *in vitro*: Six human HNSCC cell lines (HPV+ UM-SCC-47, UD-SCC-2; HPV–Cal33, HSC4) and two healthy human fibroblast cultures (SBLF7, SBLF9)	Combined IR with DDR inhibitors AZD0156 (ATM) and VE-822 (ATR) induced pronounced senescence and necrosis in selected cancer cell lines (e.g., Cal33 and UM-SCC-47), whereas CC-115 (DNA-PKcs/mTOR inhibitor) did not. Induction of senescence and necrosis varied across cell lines, independent of HPV status or HR proficiency. DDR inhibitor treatment sometimes reduced senescence in normal fibroblasts or specific tumor lines, highlighting molecular heterogeneity. Combined DDR inhibition and IR effects were cell-line-specific and sometimes associated with abrogation of G2 arrest	32883016
Preclinical *in vitro*: Six lung cancer cell lines with diverse DDR deficiencies (including TP53 and ATM mutations), including A549 TP53 knockout	Radiopotentiation profiling showed that AZD0156 (ATM inhibitor) and KU-60648 (DNA-PKcs inhibitor) were strong radiosensitizers. AZD0156 preferentially sensitized *TP53*-deficient cells, whereas KU-60648 caused steep dose-dependent radiosensitization across all cell lines. Olaparib, ceralasertib (ATR inhibitor), and adavosertib (WEE1 inhibitor) were milder radiosensitizers with moderate dose-dependent effects. Effective radiosensitization occurred at lower concentrations than required for single-agent efficacy, highlighting the importance of genetic background (e.g., *TP53* status) and drug concentration.	34158341
Preclinical *in vitro*: Five melanoma cell lines of primary and metastatic origin; two healthy skin fibroblast controls	AZD0156 (ATM inhibitor) synergistically radiosensitized melanoma cells in colony formation assays, while sparing healthy fibroblasts. VE-822 (ATR inhibitor) led to additive or short-term toxicity but less synergistic radiosensitization. AZD0156 combined with IR increased G2/M cell cycle arrest in melanoma cells. Melanoma lines with defective HR were more sensitive to ATM inhibition, while ATR inhibition effects were more variable. Healthy fibroblasts with intact HR were minimally affected.	36229655
Preclinical *in vitro*: MCF-7 breast cancer cells (ER-positive) and WI-38 healthy lung fibroblasts	AZD0156 + irradiation did not significantly increase apoptosis in MCF-7 cells but induced G0/G1 cell cycle arrest and significantly reduced clonogenic survival, especially at low radiation doses (2–4 Gy). In WI-38 fibroblasts, apoptosis slightly increased, but cell cycle and clonogenic survival were minimally affected. High-dose IR (≥6 Gy) diminished AZD0156 benefit in tumor cells.	37006058
Preclinical *in vitro*: MDA-MB-231 (triple-negative) and MCF-7 (ER-positive) breast cancer cells	ATM inhibitor AZD0156 was the strongest radiosensitizer, enhancing both single-dose and fractionated IR. ATR inhibitor AZD6738 and PARP inhibitor olaparib also radiosensitized cells, but less potently. Radiosensitization was associated with more residual DSBs 24 h post-IR and disruption of sublethal damage repair, while cell cycle distribution was not significantly altered. Fractionated IR showed greater enhancement than single-dose IR	35954456
Preclinical *in vitro* and xenograft: BT549, U2OS, MDA-MB-436 (BRCA1−/− and BRCA1+/+), V79, VC8	Proton Bragg peak irradiation induced more complex DNA DSBs preferentially repaired via HR. ATM inhibition (AZD0156) blocked resection, shunting DSBs to toxic NHEJ, enhancing cell killing effects. HR-deficient tumors were intrinsically more sensitive to Bragg peak irradiation; HR-proficient tumors become sensitized when co-treated with AZD0156. ATR inhibition did not enhance Bragg peak RBE. NHEJ ligation proteins XRCC4/Lig4 were key determinants of response.	33597272
Preclinical *in vitro* and PDX models: 12 CRC cell lines (HCT8, RKO, LOVO, HT29, LS180, LS513, GPD2, LS1034, HCT116, DLD1, SW48, Colo678) and 4 PDX models (CRC026, CRC102, others)	AZD0156 alone showed limited activity. Combination with irinotecan (SN38) enhanced anti-proliferative effects, increased G2/M arrest, and elevated DNA damage markers (γH2AX, phosphorylated CHK2). Clonogenic regrowth was reduced in selected cell lines. Triplet combination with 5FU did not add significant benefit over doublet AZD0156 + irinotecan. Variable responses observed across models, likely due to differing DDR mutation profiles	31291716
Preclinical *in vitro* and *in vivo* PDAC models: Human PDAC lines (MIA PaCa-2, PANC-1, HPAF-II, AsPC-1), murine KPC-derived lines (K8484, DT8082), ATM knockout/WT variants, NSG mouse xenografts	*ATM*-null cells were hypersensitive to ATR inhibitor AZD6738 + gemcitabine (ATRi/gem), showing increased replication catastrophe, pan-nuclear DNA damage, and persistent phospho-CHK2 and phospho-KAP1 formation via DNA-PKcs activity. ATM-low (partial) cells were not hypersensitive to therapy. ATRi/gem induced tumor growth delay in ATM-*WT* xenografts and regression in ATM-*KO* xenografts. Low-dose gemcitabine effectively sensitized tumors to AZD6738 *in vivo* without excessive toxicity.	32741974
Human biliary tract cancer (BTC) cell lines (RBE, HuCCT1, SSP-25, TGBC-24TKB, TFK-1, SNU-308, SNU-1196), including gemcitabine-resistant sublines	*POLQ* is downregulated in gemcitabine-resistant BTC. ATM inhibition (AZD0156) selectively killed gemcitabine-resistant and *POLQ*-deficient cells. *POLQ* knockdown alone did not affect proliferation, but combined with AZD0156 induced synthetic lethality, increasing DNA damage accumulation, micronucleus formation, apoptosis, and suppressed colony formation. DNA damage from photons, ROS, or chemotherapy enhanced the synthetic lethal effect.	33182492
Preclinical lung cancer models: Nine lung cancer cell lines (H157, H1563, H727, H358, H446, H460, H23, H1299, A549); 123 primary lung cancer tissue samples; H1299 xenografts in mice	*TMEM176A* is methylated in ~54% of primary lung cancers. Methylation reduces *TMEM176A* expression, which normally suppresses proliferation, invasion, migration, colony formation, and induces G2/M arrest and apoptosis. *TMEM176A* methylation activates ERK signaling. Methylated *TMEM176A* sensitizes H1299 and H23 cells to ATM inhibition (AZD0156), but not ATR inhibition (AZD6738). Restoration of *TMEM176A* decreases ATM inhibitor sensitivity.	34558311
Preclinical glioma models: Human pediatric and adult glioblastoma (KNS42, SJ-GBM2, U251, SF188, UW479) with isogenic ATRX knockout or wild-type; Mouse glioblastoma neurospheres (shATRX/shp53/NRAS models) and orthotopic xenografts;	ATRX binds promoters/enhancers of cell cycle genes (notably CHK1), and *ATRX* loss reduces *CHK1* expression, causing early G2/M entry and cell cycle dysregulation. *ATRX*-deficient cells showed elevated ATM activity, which protected against mitotic catastrophe. ATM inhibition in *ATRX*-deficient cells abolished this protection, sensitizing cells to apoptosis and irradiation. *ATRX*-deficient xenografts treated with AZD0156 showed doubled median survival, whereas *ATRX*-wild-type tumors were unaffected. Mechanism involved epigenetic dysregulation via altered H3.3 deposition at cell cycle genes.	35021084
Preclinical T-cell lymphoma models: Malignant T-cell lines (KARPAS-299, SUP-T1, HH, HD-MAR-2, JURKAT, OCI-Ly12); healthy T lymphocytes as controls	Intrinsic DNA damage and basal DDR activation are present in T-cell lymphoma models. ATM inhibition with AZD0156 disrupted DDR signaling (e.g., γH2AX phosphorylation) without impairing 53BP1 foci formation, preventing efficient repair and promoting apoptosis when combined with CHOEP. Response was heterogeneous across cell lines. ATM inhibition alone had minimal cytotoxicity, but synergized with chemotherapy. Mild G2/M arrest was observed only in combination treatment.	35409194
Esophageal squamous cell carcinoma (ESCC) models: 8 ESCC cell lines (KYSE30, KYSE140, KYSE150, KYSE180, KYSE450, KYSE510, KYSE520, colo680n), xenografts in mice; 142 esophageal dysplasia and 1007 primary ESCC patient samples	*SLFN11* is frequently methylated (25.6% in ESCC) and silenced by promoter methylation. SLFN11 normally activates ATR and NHEJ pathways while inhibiting ATM/CHK2 signaling. Epigenetic silencing of *SLFN11* switched DDR signaling, making cells more sensitive to ATM inhibition (AZD0156). *SLFN11*-methylated cells exhibited synthetic lethality with AZD0156 *in vitro* and *in vivo*. *SLFN11* methylation correlated with tumor differentiation and size.	38764821
Uterine leiomyosarcoma (LMS) patient samples: Genomic profiling of clinical samples	Uterine LMS exhibited high HR deficiency scores, overexpressed *PLK4*, and showed frequent mutations in DNA repair genes (*BRCA2* 48%, *RAD51B* 48%, *ATM* 28%, *CHK2* 24%). ATM/phosphorylated-ATM protein expression was reduced or lost in most tumors.	38848043
Gynecologic sarcoma cell lines: SK-UT-1, SKN, SK-LMS-1, BRCA2-KO SK-UT-1	PLK4 inhibition via CFI-400945 induced chromosome missegregation, DSBs, apoptosis, and HR repair activation. Combining CFI-400945 with ATM inhibitor AZD0156 increased DNA damage via suppression of HR and promotion of error-prone NHEJ. BRCA2-*KO* cells were hypersensitive to PLK4 inhibition due to defective HR/NHEJ repair.	
*In vivo* mouse xenografts (SK-UT-1 in Balb/c nude mice)	Oral CFI-400945 reduced tumor growth at low and high doses. Combination with AZD0156 further enhanced efficacy without observed toxicity. Reduced proliferation was confirmed by PHH3 staining.	
Preclinical *in vitro* and *in vivo* models: Murine syngeneic tumor models (MOC2 HNSCC, B78 melanoma), IFNAR1-KO mice, STING-deficient tumor cells, CD8^+^ T cell depletion, and bilateral tumor models	RT combined with low-dose AZD0156 significantly reduced tumor growth compared with either treatment alone. The combination synergistically increased tumor-derived *IFN-β*, *MHC-I*, and *PD-L1* expression, enhanced CD8^+^ T cell infiltration and activation, and required both tumor-intrinsic STING and host IFNAR1 signaling. Anti-PD-L1 therapy further amplified antitumor response. Low-dose AZD0156 alone had minimal effect, but effectively potentiated RT-induced immunogenicity.	37730275
Preclinical *in vitro* and *in vivo* models: Human pancreatic cancer cell line Panc1, murine pancreatic cancer cell lines mT4 and KPC2, syngeneic immunocompetent mouse models, cGAS/STING/TBK1 knockouts,	ATM inhibitors (AZD1390 and AZD0156) enhanced radiation-induced *T1IFN* expression via the POLIII/RIG-I/MAVS pathway, independent of cGAS/STING. Combination therapy increased intratumoral CD8^+^ T cell frequency, activation, and effector function, leading to systemic tumor control, immunogenic memory, and sensitization to anti-PD-L1 therapy. Therapeutic efficacy was CD8^+^ T cell–dependent. ATM inhibition also up-regulated *PD-L1* expression following radiation and potentiated adaptive antitumor immunity.	38376927
Preclinical *in vitro* models: HPV-negative (HSC4, Cal-33) and HPV-positive (UM-SCC-47, UD-SCC-2) HNSCC cell lines	Both ATMi and ATRi alone had minimal cytotoxicity, but combination with RT enhanced cell death. RT + ATRi predominantly induced necrosis and increased immunostimulatory immune checkpoint markers (ICOS-L, CD137-L), while RT + ATMi was associated with apoptosis and a more immunosuppressive phenotype, including downregulation of *RIG-1* and upregulation of *EDIL3*. Cytokine profiling showed RT + ATRi increased production of pro-inflammatory mediators (IL-6, IL-8, TNF-α, IL-12p70, IL-13), whereas RT + ATMi decreased several cytokines (IL-6, IL-8, IL-10, IL-13, IL-1β), highlighting differential immune modulation. Effects were cell line–specific rather than strictly HPV-status dependent.	39411143
Preclinical *in vitro*: HPV-negative (HSC4, Cal33) and HPV-positive (UM-SCC-47, UD-SCC-2) HNSCC cell lines; co-culture with NK cells	DDR inhibitors alone had minimal impact on clonogenic survival. Combination with RT significantly reduced cell survival. ATMi + RT induced cellular senescence (morphology changes, β-galactosidase activity, SASP cytokine release IL-6, IL-8), particularly in HPV-negative lines. Senescent tumor cells stimulated NK cell activation markers (NKG2D, NKP46, NKP44, NKP30) in co-culture, suggesting enhanced immune surveillance. ATRi + RT also reduced clonogenicity but induced less senescence and NK activation. IL-8 secretion was reduced by ATMi + RT, potentially relieving NK suppression. Senescence induction varied by cell line (e.g., UD-SCC-2 less senescent due to G2 accumulation and cell death).	40883442

#### 3.1.8 AZD1390

AZD1390 is a next-generation, highly potent and selective ATM kinase inhibitor developed specifically to overcome one of the major limitations in CNS oncology: inadequate blood–brain barrier (BBB) penetration of DDR–targeted therapies. AZD1390 exhibits sub-nanomolar cellular potency (IC_50_ ≈ 0.78 nM), exceptional selectivity (>10,000-fold over other PIKK family members), and favorable oral bioavailability, with BBB penetration validated by positron emission tomography imaging using ^11^C-labeled AZD1390 in non-human primates [190].

Pharmacodynamically, AZD1390 potently suppresses ATM-dependent signaling following IR, inhibiting phosphorylation of canonical substrates such as ATM-Ser1981 and RAD50, abrogating G2/M checkpoint arrest, promoting micronuclei formation, and inducing apoptosis. These effects translate into robust radiosensitization across glioma, lung cancer, and metastatic brain tumor models, with particularly strong responses observed in *TP53*-mutant tumors, where elevated basal ATM signaling and HR dependency create a heightened vulnerability to ATM inhibition [190,191].

Preclinical efficacy of AZD1390 has been demonstrated in multiple orthotopic and patient-derived xenograft models, including GBM, pediatric high-grade glioma, breast cancer brain metastases, lung cancer brain metastases, and small cell lung cancer, where combination with fractionated whole-brain or stereotactic RT significantly prolonged survival compared with radiation alone [96,190,192].

While AZD1390 is a substrate for P-GP–mediated efflux, PK studies show that therapeutically relevant free drug concentrations are achieved within tumor core and infiltrative tumor regions, exceeding radiosensitizing thresholds even in normal brain tissue [193]. Importantly, resistance mechanisms to AZD1390-mediated radiosensitization have been identified, including attenuation of ATM pathway responsiveness and compensatory ATR dependence, suggesting rational combination strategies with ATR inhibitors to overcome adaptive resistance [93].

Beyond radiosensitization, AZD1390 engages synthetic lethal interactions across multiple genetic contexts. In *BRCA1*-deficient breast cancer, combined inhibition of ATM and the epigenetic regulator enhancer of zeste homolog 2 (EZH2) induces profound genotoxic stress and apoptosis, producing durable tumor control *in vivo* [194]. In hematologic malignancies such as acute myeloid leukemia, AZD1390 synergizes with DNA (cytosine-5)-methyltransferase 1 (DNMT1) and ATR inhibitors to suppress proliferation and induce apoptosis [195]. AZD1390 also enhances the efficacy of DNA-damaging chemotherapies, including cisplatin, by increasing reactive oxygen species, mitochondrial dysfunction, G2/M arrest, and apoptotic cell death in breast cancer models [89].

Emerging evidence further implicates ATM inhibition as a modulator of anti-tumor immunity. AZD1390 activates innate immune signaling through cGAS–STING–IFNβ pathways, leading to upregulation of galectin-9 and adaptive immune resistance. Therapeutic neutralization of galectin-9 in combination with AZD1390 markedly enhances T-cell infiltration and tumor control in immunotherapy-resistant syngeneic models [196].

From a translational perspective, despite being metabolized by aldehyde oxidase and exhibiting high hepatic clearance, AZD1390 demonstrates acceptable clinical PK, confirmed brain penetrance in healthy volunteers, and sustained progression into GBM clinical trials [197].

Collectively, these studies position AZD1390 as a clinically advanced, BBB-penetrant ATM inhibitor with broad utility as a radiosensitizer, synthetic lethal partner, and immunomodulatory agent across genetically defined, DDR-dependent malignancies [93,191,192,193] (Table 5).

**Table 5:** Key preclinical insights derived from studies involving use of AZD1390 as ATM inhibitor.

Model/System	Key Findings	PMID
Preclinical *in vitro* and *in vivo* models: glioma and lung cancer cell lines; syngeneic GL261 glioma model; patient-derived glioblastoma (GBM) xenografts (TMZ-sensitive and -resistant); orthotopic lung-to-brain metastasis models	AZD1390 is a potent, selective, brain-penetrant ATM inhibitor that suppresses DDR signaling, enhances radiosensitivity (especially in *TP53*-mutant tumors), and significantly improves tumor control and survival when combined with radiotherapy in brain and lung cancer models.	29938225
Preclinical *in vitro* and *in vivo* models: IDH1–wild-type glioblastoma (GBM) cell lines; U251 cells; 10 orthotopic GBM patient-derived xenograft (PDX) models stratified by TP53 status; plasmid-based DNA repair reporter assays; TCGA transcriptomic analysis	AZD1390 suppresses ATM signaling and selectively radiosensitizes *TP53*-mutant GBM by disrupting G1 arrest and HR, leading to enhanced apoptosis and improved survival outcomes in mutant PDX models.	38354228
Preclinical *in vitro* and *in vivo* models: early-passage pediatric high-grade glioma (pHGG) cell lines across molecular subgroups; TP53 wild-type and TP53-mutant isogenic pHGG models; orthotopic xenografts including SJ-DIPG7 and SJ-DIPG37 patient-derived models	An unbiased DDR inhibitor screen identified the ATM inhibitor AZD1390 as a potent radiosensitizer in pHGG. AZD1390 significantly enhanced the efficacy of radiation across molecular subgroups by increasing genomic instability and mutagenic DNA repair. Contrary to prior reports, radiosensitization by ATM inhibition was observed in both *TP53* wild-type and *TP53*-mutant pHGG cell lines and orthotopic xenograft models. *In vivo*, AZD1390 combined with fractionated whole-brain radiation significantly delayed tumor progression and improved survival in multiple orthotopic models. A resistance phenotype to AZD1390 plus radiation was identified, characterized by attenuated ATM pathway activation and associated synthetic lethality with ATR inhibition	36971093
Preclinical *in vivo* patient-derived xenograft (PDX) models of breast cancer CNS metastases: three DDR-mutant breast cancer CM PDXs (CM07 ER+/HER2+, CM14 HER2+, CM16 TNBC) implanted subcutaneously in the flank; orthotopic intracranial implantation of CM14	Pretreatment with AZD1390 followed by radiation significantly inhibited tumor growth in all three flank-implanted CM PDX models, independent of hormone receptor or *HER2* status. In the orthotopic intracranial CM14 model, AZD1390 combined with radiation significantly improved survival (mean survival 222 days vs. 123 days in controls). Post-radiation adjuvant AZD1390 treatment for 21 days did not provide additional benefit. While sustained tumor control was observed in most animals, a subset developed late tumor regrowth 50–100 days after treatment. Gene expression analysis of progressing tumors showed upregulation of oncogenic pathways compared with complete responders.	37585496
Preclinical *in vitro* and *in vivo* SCLC models: CRISPR-Cas9 radiosensitization screen in SBC5 SCLC cells; multiple human SCLC cell lines (SBC5, SHP77, H446, KP1, H1048); *in vivo* murine syngeneic KP1 model and patient-derived xenograft (PDX) models JHU-LX108 and JHU-LX33 treated with radiation ± ATM inhibito	CRISPR and pharmacologic inhibition identified ATM as a key mediator of SCLC radioresistance. AZD1390 suppressed ATM signaling and synergistically enhanced radiation-induced DNA damage and tumor growth delay *in vitro* and *in vivo*.	38104868
Preclinical pharmacokinetic and tumor distribution studies in mice: wild-type, P-gp knockout, Bcrp knockout, and dual knockout mouse models; intracranial GBM patient-derived xenograft (PDX G12) model evaluating CNS and tumor distribution of the ATM inhibitor	AZD1390 CNS exposure in mice was limited by active efflux mediated predominantly by P-GP, with low unbound fractions in brain and spinal cord. Pharmacological inhibition of efflux increased CNS exposure. No regional differences in AZD1390 distribution were observed across anatomic regions of the CNS. In an intracranial GBM PDX model, AZD1390 accumulated to higher levels in the tumor core and rim compared with surrounding normal brain, and concentrations in all brain regions exceeded *in vitro* radiosensitizing thresholds despite heterogeneous distribution.	36137710
Preclinical *in vitro* and *in vivo* models of BRCA1-deficient breast cancer: murine mammary tumor cell lines derived from BRCA1-deficient and -proficient mice; human BRCA1-mutant TNBC cell lines; BRCA1-deficient mammary tumor–bearing mouse models	Combined EZH2 (GSK126) and ATM (AZD1390) inhibition induced synthetic lethality in *BRCA1*-deficient breast cancer, increasing DNA damage and apoptosis and producing strong antitumor effects *in vitro* and *in vivo*.	35715861
Preclinical AML models: human AML cell lines (MOLM-16, NB-4, HEL 92.1.7, HEL, EOL-1); bioinformatic analyses of AML patient datasets (TCGA, CCLE)	*DNMT1*-related gene network analysis identified ATM and ATR as dominant upstream kinases associated with DNMT1 signaling in AML. *DNMT1*, *ATM*, and *ATR* were significantly upregulated in AML patient samples and cell lines. Pharmacologic inhibition of DNMT1 (GSK-368), ATM (AZD-1390), or ATR (AZD-6738) demonstrated dose-dependent anti-leukemic activity as monotherapies. Combination treatment with DNMT1 and ATM or ATR inhibition, as well as dual ATM/ATR inhibition, produced marked synergistic growth suppression and apoptosis across all AML cell lines tested, exceeding the effects of single-agent therapy. These effects support functional interaction between epigenetic regulation and DDR in AML.	38164802
Preclinical *in vitro* breast cancer models: human breast cancer cell lines MCF-7 and MDA-MB-231	AZD1390 synergized with cisplatin to inhibit breast cancer cell growth by increasing DNA damage, ROS, G2/M arrest, and apoptosis, leading to reduced viability and clonogenic survival in both HR-positive and triple-negative cell lines	39681282
Preclinical *in vitro* and *in vivo* immunocompetent models: multiple human and murine tumor cell lines; syngeneic mouse models including CT26 colon cancer, MC38, B16-F10 melanoma, and poorly immunogenic LLC lung tumors	ATM inhibition (AZD1390 or genetic loss) activated cGAS–STING–IFNβ signaling but also induced Galectin-9–mediated immune suppression. Combining ATM inhibition with anti–Gal-9 therapy enhanced T-cell infiltration and antitumor efficacy, overcoming immune escape and improving survival in multiple tumor models.	36778120
Human *in vitro* metabolism systems (human hepatocytes and liver cytosol); physiologically based pharmacokinetic (PBPK) simulations; first-in-human clinical pharmacokinetic evaluation; healthy volunteer PET imaging; glioblastoma clinical trial setting	AZD1390 is a potent, selective ATM inhibitor with AO-mediated high clearance but adequate oral exposure and confirmed brain penetration. PK/PD and PBPK analyses supported sufficient systemic and CNS exposure for clinical use with radiotherapy.	40714318

#### 3.1.9 M3541

Preclinical studies have demonstrated that pharmacologic inhibition of ATM with M3541 suppresses DSB repair, abrogates ATM-dependent checkpoint signaling, and enhances clonogenic cell death following radiation or chemotherapy exposure. A genome-wide clustered regularly interspaced short palindromic repeats (CRISPR) screening approach identified defects in the Fanconi anemia (FA)/BRCA pathway as strong genetic vulnerabilities conferring hypersensitivity to M3541, revealing a synthetic lethal interaction between ATM inhibition and FA/BRCA deficiency. Mechanistically, concurrent loss of ATM and FA/BRCA function profoundly suppresses DNA end resection, shifts repair toward error-prone NHEJ, and generates toxic chromosomal aberrations that culminate in cell death, supporting the therapeutic potential of ATM inhibition in HR–deficient tumors [198]. *In vivo*, oral administration of M3541 markedly potentiated the antitumor effects of clinically relevant fractionated RT, leading to durable tumor regressions in xenograft models, with pharmacodynamic (PD) modulation of ATM signaling correlating with efficacy [85].

Beyond radiosensitization, M3541 demonstrated strong combinatorial activity with PARP inhibitors, TOP I inhibitors, and ATR inhibitors, highlighting its capacity to amplify replication stress and chromosomal instability when checkpoint safeguards are disabled [85,199]. At the cellular level, ATM inhibition by M3541 drives aberrant mitotic entry with unresolved DNA damage, resulting in micronuclei formation, activation of the cGAS–STING inflammatory pathway, and enhanced susceptibility to immune-mediated killing, while simultaneously inducing *PD-L1* expression [101].

Clinically, a first-in-human phase I study evaluated M3541 in combination with palliative RT in patients with advanced solid tumors and demonstrated acceptable tolerability up to 300 mg administered on radiation fraction days, with preliminary evidence of antitumor activity. However, the absence of dose-proportional PK and a clear exposure–response relationship ultimately limited further clinical development [200] (Table 6).

**Table 6:** Key preclinical insights derived from studies involving use of M3541 as ATM inhibitor.

Model/System	Key Findings	PMID
Genome-wide CRISPR-Cas9 screening in non–small cell lung cancer cell lines (A549, NCI-H460); validation in multiple human cancer cell lines with defined DNA repair defects	A genome-wide CRISPR screen identified loss of Fanconi anemia (FA)/BRCA pathway genes as a major genetic vulnerability that confers hypersensitivity to ATM inhibition. Knockout of FA/BRCA pathway genes markedly sensitized lung cancer cells to the ATM inhibitor M3541. Combined disruption of ATM and FA/BRCA pathway function led to impaired DSB end resection, increased reliance on NHEJ, reduced HR, and accumulation of toxic DNA repair intermediates, resulting in synthetic lethality. Additional genetic contexts associated with ATM inhibitor sensitivity included loss of *RAD18*, *PTEN*, or *STAG2*.	32075772
Preclinical *in vitro* cancer cell line panels; immunodeficient mouse xenograft models treated with clinically relevant fractionated radiotherapy; combination studies with PARP and topoisomerase inhibitors	M3541 and M4076 are potent, selective ATM inhibitors that impair DNA repair and checkpoint control. Compounds strongly radiosensitized tumors, and showed synergistic antitumor activity with radiotherapy, PARP, and TOP1 inhibitors *in vitro* and *in vivo*, with M4076 progressing to clinical development.	35405736
*In vitro* cancer cell lines (ATM- and p53-proficient and deficient)	ATR inhibition alone activated ATM–TP53–mediated G1 checkpoint, limiting cytotoxicity. Pharmacologic ATM inhibition (M3541, M4076) disabled both ATM-dependent DNA repair and checkpoint control, prolonged persistence of ATR inhibitor–induced DSBs, increased abundance of chromosomal aberrations, and markedly enhanced cancer cell death.	37079339
Isogenic A549 models (ATM-null, p53-null, wild-type)	Full suppression of ATM function (genetic or pharmacologic) was required for maximal ATR inhibitor efficacy. ATM inhibitors phenocopied *ATM*-null status, restoring ATR inhibitor sensitivity even in *ATM*- and *TP53*–wild-type cells.	
Broad cancer cell line panel (34 tumor-derived lines)	Synergistic cytotoxicity between ATR inhibitors (M6620, M4344) and ATM inhibitors across diverse tumor types and genetic backgrounds was observed, indicating compound-agnostic and tumor-agnostic synergy.	
Mouse xenograft models	ATM inhibition significantly enhanced ATR inhibitor efficacy *in vivo* at well-tolerated doses, without overt toxicity. Increased efficacy correlated with enhanced chromosomal damage and mitotic catastrophe.	
Triple-negative breast cancer (TNBC) PDX models (n = 26)	Combination of ATR inhibitor M4344 with ATM inhibitor M4076 achieved a 42% RECIST-relevant tumor control rate (SD + PR + CR), outperforming ATR inhibitor monotherapy and improving survival.	
Human cancer cell lines (A549, HeLa, H1299, HT1080, RKO, A375, others), metaphase chromosome spreads, micronucleated cancer cells, co-culture with NK cells, surviving irradiated cancer cells, and *in vivo* xenograft models	M3541/M4076 ATM inhibitors abrogated DDR checkpoints and DSB repair, causing mitotic catastrophe and micronuclei-driven cGAS–STING immune activation, enhancing tumor regression with radiotherapy and increasing susceptibility to NK cell killing with minimal toxicity *in vivo*.	36656721
Patients with advanced solid tumors receiving fractionated palliative radiotherapy (30 Gy in 10 fractions; N = 15), doses of oral ATM inhibitor M3541 50–300 mg/fraction day	M3541 was generally well tolerated with manageable toxicity and no clear dose-dependent adverse events; pharmacokinetics showed non-linear exposure, and limited clinical responses were observed, likely driven by radiotherapy rather than drug effect.	35150356

### 3.2 Planned or Ongoing Clinical Trials

#### 3.2.1 KU-60019

One registered clinical trial (NCT03571438; phase not clearly specified/early exploratory) aims to evaluate the therapeutic potential of KU-60019 in combination with a casein kinase 2 (CK2) inhibitor for metastatic clear cell renal cell carcinoma (ccRCC), a tumor type historically resistant to both chemotherapy and RT. The trial leverages organotypic cultures derived from patient tumors to assess drug efficacy in an *ex vivo* environment that more faithfully recapitulates the tumor microenvironment, including hypoxic niches and three-dimensional architecture. Preliminary preclinical studies indicate that the CK2–ATM inhibitor combination exerts synergistic cytotoxic effects, particularly in Von Hippel–Lindau (*VHL*)-deficient ccRCC cells under hypoxic conditions, and is more effective in 3D culture models than in conventional 2D cultures. This strategy exemplifies a rational polypharmacological approach aimed at overcoming adaptive resistance mechanisms, such as mechanistic target of rapamycin complex 2 (mTORC2)-mediated AKT activation, which limit the efficacy of current targeted therapies, including vascular endothelial growth factor (VEGFR) and mTOR inhibitors (sunitinib, pazopanib, and temsirolimus). By simultaneously inhibiting multiple kinases that converge on survival and DNA repair pathways, KU-60019 may potentiate tumor cell death while reducing the likelihood of therapeutic escape. While the clinical trial status remains unclear, the integration of KU-60019 into combination regimens for ccRCC highlights the growing interest in ATM inhibitors as adjuncts to targeted therapy, particularly in tumors with intrinsic resistance or deregulated signaling networks.

#### 3.2.2 WSD0628

WSD0628 is under clinical investigation as a radiosensitizer for recurrent high-grade gliomas (HGGs), a patient population with a notoriously poor prognosis despite aggressive multimodal therapy. ATM plays a central role in the DDR, orchestrating repair of DSBs and regulating cell-cycle checkpoints, making it a compelling target to enhance the cytotoxic efficacy of IR. The ongoing phase 0/I clinical trial (NCT05917145, recruiting) is evaluating the safety, tolerability, PK, PD, and preliminary antitumor activity of WSD0628 in combination with radiation therapy in adult patients with recurrent HGG. The trial employs a Bayesian Optimal Interval (BOIN) dose-escalation design (Part A, ~24 patients) to establish the recommended phase 2 dose (RP2D), followed by a dose-expansion cohort (Part B, ~12 patients) to further assess safety and efficacy, and a phase 0 tumor-penetrance arm (~6 patients) to quantify drug delivery to brain tissue. Preclinical PK-PD-efficacy modeling guides dose selection by correlating plasma and tumor concentrations of WSD0628 with robust ATM inhibition and radiosensitization. Efficacy endpoints include progression-free survival, overall survival, and radiographic response per the Response Assessment in Neuro-Oncology (RANO) criteria, while safety endpoints encompass adverse events and laboratory analyses. By selectively inhibiting ATM, WSD0628 is expected to impair DSB repair, abrogate G1/S and G2/M checkpoint controls, and enhance radiation-induced mitotic catastrophe and apoptosis in tumor cells, potentially overcoming the intrinsic radioresistance of HGG.

#### 3.2.3 AZD0156

The completed phase I clinical trial (NCT02588105) evaluated the safety, tolerability, PK, and preliminary efficacy of AZD0156 alone or in combination with olaparib, irinotecan, or FOLFIRI in patients with advanced solid tumors. The study was organized into multiple modules, each exploring a specific combination, with dose-escalation conducted first for monotherapy to establish PK and safety profiles, followed by combination dose-escalation. Expansion cohorts further assessed safety, tolerability, biological activity, and preliminary antitumor efficacy, including in locally advanced/metastatic gastric adenocarcinoma (module 1) and colorectal cancer (module 2). AZD0156 is expected to sensitize tumor cells to DNA-damaging agents by inhibiting ATM-mediated repair of radiation- or chemotherapy-induced DSBs, abrogating G1/S and G2/M checkpoints, and promoting apoptosis or mitotic catastrophe, particularly in tumors with defects in HR. Nevertheless, result were not published so far.

#### 3.2.4 AZD1390

There are currently 7 clinical trials investigating AZD1390 in cancer treatment registered on clinicaltrials.gov.

AZD1390 is being evaluated in several early-phase clinical trials across adult and pediatric populations. In GBM and brain metastases, phase I studies (NCT03423628, NCT05182905) are assessing safety, tolerability, PK, and preliminary efficacy when AZD1390 is combined with fractionated RT, including intensity-modulated radiation therapy (IMRT) and whole-brain RT. Pediatric trials (NCT06894979) are exploring similar endpoints in high-grade gliomas, diffuse midline gliomas, and diffuse intrinsic pontine gliomas, with additional evaluation of neurocognitive outcomes, PD, and biomarkers such as HR deficiency and circulating free DNA (cfDNA). Beyond brain tumors, AZD1390 is being tested in soft tissue sarcoma (STS) in combination with RT, with or without the immune checkpoint inhibitor durvalumab (NCT05116254), aiming to improve R0 resection rates and stimulate tumor T-cell infiltration in immunologically “cold” tumors. Other trials, including NSCLC (NCT04550104) and metastatic solid tumors (NCT05678010), are assessing AZD1390 with curative-intent RT or stereotactic body radiation therapy (SBRT), with the goal of optimizing recommended phase II doses while monitoring long-term toxicity, efficacy, and integration with immunotherapy consolidation.

#### 3.2.5 M3541

Clinically, M3541 was evaluated in a phase I, open-label, dose-escalation trial (NCT03225105) in patients with solid tumors receiving fractionated palliative RT to thoracic, abdominal, head and neck, or extremity lesions. The study aimed to assess safety, tolerability, PK, PD, and preliminary antitumor activity. M3541 doses up to 300 mg per fraction day were generally well tolerated, with no dose-limiting toxicities leading to treatment discontinuation; however, PK analysis revealed limited dose-proportional exposure and a lack of a clear PD dose-response relationship. Despite early indications of safety, the trial was terminated prematurely by the sponsor due to suboptimal PK and PD properties that hindered further clinical development.

#### 3.2.6 SYH2051

Clinically, SYH2051 is being investigated across multiple phase I and phase I/II studies in advanced solid tumors, including breast, gastrointestinal, and head and neck cancers. For instance, in NCT06775236, a phase 1b/2 study, SYH2051 is combined with SYS6010 in *EGFR*-expressing, unresectable, locally advanced, or metastatic breast cancer to determine safety, tolerability, and the recommended phase 2 dose (RP2D), followed by efficacy assessment against standard-of-care chemotherapy. Dose-escalation cohorts use a traditional “3 + 3” design, exploring a range of SYH2051 doses (40–80 mg) in combination with SYS6010, with subsequent expansion cohorts focusing on specific molecular subtypes. Other studies, including NCT07104877, investigate SYH2051 with SYS6010 in advanced gastrointestinal tumors, while NCT06011291 explores SYH2051 monotherapy or in combination with RT for locally advanced head and neck cancer, assessing PK, safety, tolerability, and preliminary antitumor activity. Collectively, these studies underscore SYH2051’s versatility as a potential radiosensitizer and combination therapy agent. However, data remain limited, with most studies still in early-phase evaluation or with unknown status, highlighting the need for further preclinical and clinical investigations to clarify its optimal therapeutic combinations, dosing regimens, and biomarker-driven patient selection strategies.

#### 3.2.7 XRD-0394

XRD-0394 is an emerging, orally bioavailable, small-molecule dual inhibitor targeting ATM and DNA-PKcs. By concurrently inhibiting these kinases, XRD-0394 disrupts repair of DSBs induced by IR or chemotherapeutic agents, enhancing tumor cell killing while potentially sparing normal tissue due to differential DDR dependency. Preclinical studies have demonstrated that XRD-0394 not only potentiates RT-induced cytotoxicity *in vitro* and *in vivo*, but also synergizes with TOP I inhibitors and PARP inhibitors, particularly in *BRCA1/2*-deficient cancer models. The first-in-human phase I clinical trial (NCT05002140) assessed XRD-0394 in combination with palliative RT in patients with metastatic, locally advanced, or recurrent solid tumors. The study utilized a standard 3 + 3 single-dose escalation design (starting at 40 mg, with planned escalation to 80 mg and 160 mg) to characterize the PK, PD, safety, and tolerability of XRD-0394. Dose-limiting toxicities and treatment-emergent adverse events were carefully monitored, while PD cohorts evaluated inhibition of radiation-induced ATM activity in tumor biopsies. XRD-0394 demonstrated a favorable safety profile and achieved plasma concentrations consistent with preclinically predicted therapeutic windows, providing strong rationale for future combination strategies with RT, PARP inhibitors, and TOP-targeting agents. These findings position XRD-0394 as a promising candidate for radiosensitization and DDR-targeted combination therapy, highlighting the potential of dual ATM/DNA-PKcs inhibition to enhance therapeutic efficacy in tumors with impaired DNA repair capacity (Table 7).

**Table 7:** Summary of ongoing/planned clinical investigations involving ATM inhibitors.

Trial ID	Phase	Drug(s) Tested	Cancer Type(s)	Objectives/Endpoints	Scientific Rationale
**NCT03571438**	Not clearly specified (early exploratory)	KU-60019 + CK2 inhibitor	Metastatic clear cell renal cell carcinoma (ccRCC)	Evaluate therapeutic efficacy in patient-derived organotypic tumor cultures and assess cytotoxic synergy	Dual inhibition of ATM and CK2 targets convergent DNA repair and survival signaling pathways. The strategy exploits vulnerabilities in *VHL*-deficient, hypoxic ccRCC, overcomes adaptive resistance (e.g., mTORC2–AKT activation), and enhances efficacy beyond current VEGFR/mTOR inhibitors using a rational polypharmacology approach.
**NCT05917145**	Phase 0/I (recruiting)	WSD0628 + radiation therapy	Recurrent high-grade gliomas (HGG)	Safety, tolerability, PK/PD, tumor penetration, RP2D; secondary efficacy (PFS, OS, RANO response)	Selective ATM inhibition impairs DSB repair and checkpoint control, enhancing radiation-induced mitotic catastrophe and apoptosis. Designed to overcome intrinsic radioresistance of HGG using PK-PD-efficacy modeling and direct tumor exposure assessment.
**NCT02588105**	Phase I (completed/results not published so far)	AZD0156 ± olaparib, irinotecan, FOLFIRI	Advanced solid tumors; gastric adenocarcinoma; colorectal cancer	Safety, tolerability, PK; preliminary efficacy and biological activity	ATM inhibition sensitizes tumors to DNA-damaging chemotherapy and PARP inhibition by disrupting DSB repair and cell-cycle checkpoints, particularly in HR-deficient tumors.
**NCT03423628**	Phase I	AZD1390 + fractionated RT	Glioblastoma multiforme (GBM)	Safety, tolerability, PK, preliminary efficacy	Brain-penetrant ATM inhibition enhances RT efficacy by blocking DSB repair in radioresistant GBM cells.
**NCT05182905**	Phase I	AZD1390 + RT	Brain metastases	Safety, tolerability, PK, preliminary efficacy	ATM inhibition augments RT-induced cytotoxicity in intracranial tumors while leveraging optimized CNS penetration.
**NCT06894979**	Phase I (pediatric)	AZD1390 + RT	Pediatric high-grade glioma, DMG, DIPG	Safety, PK/PD, neurocognitive outcomes, biomarker analysis	ATM inhibition sensitizes pediatric brain tumors to RT while enabling biomarker-driven assessment of DDR dependency and treatment response.
**NCT05116254**	Phase I/II	AZD1390 + RT ± durvalumab	Soft tissue sarcoma (STS)	Improve R0 resection rate; immune and efficacy endpoints	ATM inhibition enhances RT-induced immunogenic cell death and may synergize with immune checkpoint blockade to convert immunologically “cold” tumors into responsive phenotypes.
**NCT04550104**	Phase I	AZD1390 + curative-intent RT	Non–small cell lung cancer (NSCLC)	Safety, RP2D, preliminary efficacy	Combines ATM inhibition with definitive RT to enhance tumor control while monitoring long-term toxicity.
**NCT05678010**	Phase I	AZD1390 + SBRT	Metastatic solid tumors	Safety, PK, efficacy	ATM inhibition potentiates high-dose focal radiation by preventing DSB repair in tumor cells.
**NCT03225105**	Phase I (terminated)	M3541 + fractionated RT	Advanced solid tumors	Safety, tolerability, PK/PD, preliminary efficacy	ATM inhibition intended to radiosensitize tumors; development halted due to suboptimal PK/PD despite acceptable safety.
**NCT06775236**	Phase 1b/2	SYH2051 + SYS6010	EGFR-expressing advanced breast cancer	Safety, RP2D, efficacy vs. SOC chemotherapy	ATM inhibition combined with targeted therapy aims to enhance tumor sensitivity by disrupting DDR and survival pathways.
**NCT07104877**	Phase I	SYH2051 + SYS6010	Advanced gastrointestinal cancers	Safety, PK, preliminary efficacy	Explores ATM inhibition as a combination strategy to sensitize GI tumors to targeted agents.
**NCT06011291**	Phase I	SYH2051 ± RT	Locally advanced head and neck cancer	Safety, PK, tolerability, preliminary antitumor activity	ATM inhibition enhances radiosensitivity by impairing DSB repair and checkpoint signaling.
**NCT05002140**	Phase I	XRD-0394 + palliative RT	Metastatic/recurrent solid tumors	Safety, tolerability, PK/PD, ATM inhibition in tumor biopsies	Dual ATM/DNA-PKcs inhibition blocks complementary DSB repair pathways, enhancing RT and supporting future combinations with PARP and TOP1 inhibitors, particularly in DDR-deficient tumors.

## 4 Limitations and Future Perspectives

### 4.1 Limitations

Despite these promising directions, PK and PD considerations, including tissue penetration (notably in the brain in case of radiosensitizing effects), retention of partial ATM activity, and off-target effects, require careful optimization to achieve maximal efficacy with minimal toxicity as reviewed by other authors [112,201,202]. For example, the development of next-generation ATM inhibitors such as WSD0628 has specifically addressed the challenge of brain delivery by incorporating medicinal chemistry strategies to minimize efflux via key transporters (e.g., breast cancer resistance protein BCRP and multi-drug resistance 1 (MDR1)), which commonly limit central nervous system exposure of small-molecule kinase inhibitors. In parallel, optimization efforts have included reducing liabilities related to cardiac toxicity (e.g., hERG interactions) and unfavorable metabolic pathways, underscoring the multifaceted nature of PK/PD refinement. From a safety perspective, available preclinical and clinical observations suggest that transient pharmacologic inhibition of ATM is generally tolerable, consistent with the relatively mild developmental phenotypes observed in *ATM*-deficient models. However, the principal dose-limiting toxicities are likely to arise from on-target radiosensitization of normal proliferative tissues rather than systemic ATM inhibition per se. Indeed, enhanced acute radiation toxicities, such as mucositis, have been consistently observed in preclinical models when ATM inhibitors are combined with radiotherapy. This highlights a critical translational challenge: while tumor radiosensitization is desirable, normal tissue radiosensitization must be carefully mitigated. Importantly, this risk may be partially alleviated in specific anatomical contexts, such as intracranial tumors, where advanced radiotherapy techniques (e.g., intensity-modulated radiotherapy) enable precise targeting and relative sparing of surrounding epithelial tissues. Furthermore, the cell cycle–dependent mechanism of ATM inhibition, primarily affecting proliferating cells in S/G_2_ phases, suggests a therapeutic window in which largely post-mitotic normal tissues, such as neurons, may be less susceptible to radiosensitization. Nevertheless, limitations of preclinical models in predicting late radiation-induced toxicities necessitate a cautious clinical approach. Accordingly, emerging clinical strategies emphasize conservative radiation planning, including stricter dose constraints to critical structures (e.g., optic apparatus, hippocampus, brainstem) and adjacent normal tissues. Such adaptive radiotherapy frameworks, combined with optimized drug exposure schedules, will be essential to balance efficacy and safety. Collectively, these considerations highlight that successful clinical translation of ATM inhibitors will depend not only on drug design but also on integrated PK/PD-guided treatment planning and precise radiotherapy delivery to minimize collateral toxicity while preserving potent anti-tumor effects [100].

Emerging evidence regarding resistance to ATM-targeted therapies remains limited, and, to date, directly validated genetic mechanisms of resistance specific to ATM inhibitors are scarce. As a result, many proposed resistance pathways are largely inferred from studies of other DDR inhibitors and homologous kinase-targeting strategies, rather than being firmly established in the context of ATM inhibition. For instance, mutations within kinase domains that reduce drug binding while preserving catalytic function have been well documented for other targeted therapies. However, comparable resistance-conferring mutations in the ATM kinase domain have not yet been robustly characterized in preclinical or clinical settings. Similarly, the concept of secondary “reversion mutations” restoring DNA repair capacity, well described in *BRCA1/2*-mutant tumors treated with PARP inhibitors, has not been conclusively demonstrated for ATMi, although it represents a plausible mechanism in tumors where ATM inhibition is synthetically lethal with HR deficiency. Additionally, adaptive rewiring of DDR signaling, such as activation of downstream effectors or compensatory upregulation of parallel pathways like ATR–CHK1, may bypass ATM inhibition. Alterations in cell cycle regulators further contribute by uncoupling checkpoint control from DNA damage signaling [203].

A critical limitation that must be explicitly acknowledged is the overreliance on genetically homogeneous preclinical models in the current ATM inhibitor literature. Most mechanistic and therapeutic evidence supporting ATM targeting has been derived from conventional immortalized cancer cell lines and standard subcutaneous xenograft models, which, despite their experimental utility, do not recapitulate the genomic heterogeneity, clonal evolution, or microenvironmental complexity of human tumors. In particular, these systems lack the dynamic interactions between tumor cells and the surrounding stromal, immune, and vascular compartments, all of which are increasingly recognized as critical modulators of DDR signaling and therapeutic resistance [204]. As a result, current preclinical datasets may overestimate the robustness and universality of ATM dependency, as they fail to capture context-dependent vulnerabilities shaped by intratumoral heterogeneity and niche-specific selective pressures. This limitation is especially relevant when considering combinatorial strategies involving ATM inhibitors and immunotherapies, as immune-mediated tumor clearance and DDR-induced immunogenic signaling cannot be adequately modeled in immunodeficient xenograft systems [205]. Therefore, there is a pressing need to validate ATM inhibitor efficacy in more physiologically relevant platforms, including PDXs, which better preserve patient-specific genomic architecture [206,207], as well as genetically engineered mouse models (GEMMs) that retain intact immune systems and tumor–stroma interactions. GEMMs, in particular, are essential for accurately evaluating the therapeutic synergy between ATM inhibition and immune checkpoint blockade, as they allow assessment of DDR-driven immune activation, antigen presentation, and tumor–immune co-evolution in an intact microenvironment [208,209,210]. Expanding the use of these advanced models will be critical to bridging the translational gap between preclinical ATM inhibitor studies and clinical efficacy, and to ensuring that proposed synthetic lethal strategies are robust across genetically diverse and immunologically competent tumor contexts.

### 4.2 Future Perspectives

Looking forward, several emerging directions could expand the clinical potential of ATM inhibition. First, integrating high-throughput genomic and functional screens to identify DDR vulnerabilities could refine patient selection for ATMi therapies, including the identification of biomarkers predictive of synthetic lethality beyond classical HR defects [203,211,212,213].

Second, combinatorial strategies with immune checkpoint inhibitors, cGAS-STING agonists, or targeted metabolic inhibitors may leverage ATM’s role in immune regulation and tumor metabolism to overcome resistance [214,215,216].

Third, development of brain-penetrant ATMi could improve outcomes in primary and metastatic central nervous system malignancies, where RT is standard but effective drug delivery is challenging [100,201]. Finally, rational sequencing or co-administration with DDR-targeted agents (e.g., PARP, ATR, DNA-PKcs inhibitors) could exploit compensatory DNA repair pathways and maximize therapeutic efficacy [217,218,219].

## 5 Conclusions

In summary, ATM represents a versatile and clinically actionable target in oncology. Its inhibition provides a multi-pronged strategy to exploit DNA repair deficiencies, enhance radiosensitivity, synergize with chemotherapeutics, and potentially remodel anti-tumor immunity. Careful molecular characterization, biomarker-guided patient selection, and strategic combination therapies will be key to translating ATM inhibition into durable clinical benefit, while addressing the challenges posed by its dual tumor-suppressive and tumor-promoting roles. The evolving landscape of DDR-targeted oncology positions ATM inhibitors as central agents in next-generation precision cancer therapy, particularly in contexts where conventional treatments fail or are limited by resistance.

## Data Availability

No new data were created or analyzed in this study. Data sharing is not applicable.

## Supplementary Materials

The supplementary material is available online at https://www.techscience.com/doi/10.32604/or.2026.082180/s1.

## Abbreviations

β-gal	Beta-galactosidase
4E-BP1	Eukaryotic translation initiation factor 4E-binding protein 1
53BP1	p53-binding protein 1
5FU	5-Fluorouracil
A-T	Ataxia-telangiectasia
ABCG2	ATP-binding cassette subfamily G member 2
AKT	Protein kinase B
ALK2	Activin receptor-like kinase 2
ALT	Alternative lengthening of telomeres
AML	Acute myeloid leukemia
AMPK	AMP-activated protein kinase
AO	Aldehyde oxidase
ATF4	Activating transcription factor 4
ATM	Ataxia-telangiectasia mutated
ATMi	ATM inhibitor
ATP	Adenosine triphosphate
ATR	Ataxia-telangiectasia and Rad3-related
ATRX	Alpha-thalassemia/mental retardation syndrome X-linked
AVO	Acidic vesicular organelle
BAX	Bcl-2-associated X protein
BBB	Blood–brain barrier
BCa	Bladder cancer
BCC	Basal cell carcinoma
BCR-ABL	Breakpoint cluster region–Abelson murine leukemia viral oncogene homolog fusion
BCRP	Breast cancer resistance protein
BED	Biologically effective dose
BOIN	Bayesian Optimal Interval
BRCA1	Breast cancer type 1 susceptibility protein
BRCA2	Breast cancer type 2 susceptibility protein
BRCT	BRCA1 C-terminal domain
BTC	Biliary tract cancer
CBP	CREB-binding protein
CCLE	Cancer Cell Line Encyclopedia
ccRCC	Clear cell renal cell carcinoma
CD137-L	CD137 ligand (4-1BBL)
CD155	Cluster of differentiation 155 (poliovirus receptor)
CD4^+^	Cluster of differentiation 4 positive (T cell)
CD8^+^	Cluster of differentiation 8 positive (cytotoxic T cell)
CDC25A/C	Dual-specificity phosphatases
CDK	Cyclin-dependent kinase
CDKN2A	Cyclin-dependent kinase inhibitor 2A
CED	Convection-enhanced delivery
cfDNA	Circulating free DNA
cFLIP	Cellular FLICE-like inhibitory protein
cGAS	Cyclic GMP-AMP synthase
CHK1	Checkpoint kinase 1
CHK2	Checkpoint kinase 2
CHOEP	Cyclophosphamide, doxorubicin, vincristine, etoposide, prednisone
CHOP	C/EBP homologous protein
CK2	Casein kinase 2
CM	Central nervous system metastases
CNS	Central nervous system
CPT	Camptothecin
CRC	Colorectal cancer
CRISPR-Cas9	Clustered regularly interspaced short palindromic repeats–CRISPR-associated protein 9
CSC	Cancer stem cell
CtBP	C-terminal binding protein
CtIP	C-terminal binding protein interacting protein
DAB2IP	Disabled homolog 2 interacting protein
DDR	DNA damage response
DGAT1	Diacylglycerol O-acyltransferase 1
DIPG	Diffuse intrinsic pontine glioma
DMAP1	DNA methyltransferase 1–associated protein 1
DMG	Diffuse midline glioma
DNA-PKcs	DNA-dependent protein kinase catalytic subunit
DNAM-1	DNAX accessory molecule 1
DNMT1	DNA (cytosine-5)-methyltransferase 1
dNTP	Deoxyribonucleoside triphosphate
DR5	Death receptor 5
DSB	DNA double-strand break
E2F1	E2F transcription factor 1
EC	Endometrial cancer
EDIL3	EGF-like repeats and discoidin I-like domains 3
EGFR	Epidermal growth factor receptor
EGFR-TKI	Epidermal growth factor receptor tyrosine kinase inhibitor
EMT	Epithelial–mesenchymal transition
ER	Estrogen receptor/Endoplasmic reticulum (context-dependent)
ERK	Extracellular signal-regulated kinase
ESCC	Esophageal squamous cell carcinoma
EZH2	Enhancer of zeste homolog 2
FA	Fanconi anemia
FANCC	Fanconi anemia complementation group C
FANCD2	Fanconi anemia complementation group D2
FANCG	Fanconi anemia complementation group G
FOLFIRI	Folinic acid, fluorouracil, irinotecan
FOXO1	Forkhead box protein O1
FOXO3a	forkhead box O3a protein
G2/M	Gap 2/mitosis phase
Gal-9	Galectin-9
GBM	Glioblastoma multiforme
GEO	Gene Expression Omnibus
GI	Gastrointestinal
GIC	Glioma-initiating cell
GLUT1	Glucose transporter 1
GEMMs	Genetically engineered mouse models
GSC	Glioma stem cell
GYN	Gynecologic
γH2AX	Phosphorylated histone H2AX
H_2_O_2_	Hydrogen peroxide
H3.3	Histone H3.3 variant
HAT	Histone acetyltransferase
HDAC	Histone deacetylase
HDAC1	Histone deacetylase 1
HER2	Human epidermal growth factor receptor 2
hERG	Human ether-à-go-go–related gene potassium channel
hESC	Human embryonic stem cell
HFF	Human foreskin fibroblast
HGG	High-grade glioma
HIF1α	Hypoxia-inducible factor 1-alpha
HMGN1	High mobility group nucleosome-binding protein 1
HNSCC	Head and neck squamous cell carcinoma
HPLC-MS	High-performance liquid chromatography–mass spectrometry
HPV	Human papillomavirus
HR	Homologous recombination
HRR	Homologous recombination repair
hTERT	Human telomerase reverse transcriptase
HU	Hydroxyurea
IC_50_	Half-maximal inhibitory concentration
ICL	Interstrand crosslink
ICOS-L	Inducible co-stimulator ligand
IDH	Isocitrate dehydrogenase
IFN	Interferon
IFN-β	Interferon beta
IFNAR1	Interferon alpha/beta receptor subunit 1
IGF-1	Insulin-like growth factor 1
IHC	Immunohistochemistry
IL	Interleukin
IMRT	Intensity-modulated radiation therapy
IR	Ionizing radiation
IV	Intravenous
JAK	Janus kinase
JNK	c-Jun N-terminal kinase
KAP1	KRAB-associated protein 1
Ki	Inhibition constant
KPC	Kras-mutant, Trp53-mutant pancreatic cancer mouse model
KRT19	Keratin 19
LC3B	Light chain 3B (microtubule-associated protein)
LDHA	Lactate dehydrogenase A
LDR	Low-dose radiation
LET	Linear energy transfer
Lig4	DNA ligase 4
LKB1	Liver kinase B1
LLC	Lewis lung carcinoma
LMS	Leiomyosarcoma
MAPK	Mitogen-activated protein kinase
MAVS	Mitochondrial antiviral signaling protein
MDC1	Mediator of DNA damage checkpoint protein 1
MDR1	Multi-drug resistance 1
MEK	Mitogen-activated protein kinase kinase
MGMT	O^6^-methylguanine-DNA methyltransferase
MGUS	Monoclonal gammopathy of undetermined significance
MHC	Major histocompatibility complex
MHC-I	Major histocompatibility complex class I
MICA	MHC class I polypeptide-related sequence A
MICB	MHC class I polypeptide-related sequence B
Mig-6	Mitogen-inducible gene 6
MM	Multiple myeloma
MMC	Mitomycin C
MMP-1	Matrix metalloproteinase-9
MRN	MRE11-RAD50-NBS1 complex
mRNA	Messenger ribonucleic acid
MTCL	Mature T-cell lymphoma
MTD	Maximum tolerated dose
mtDNA	Mitochondrial DNA
mTOR	Mechanistic target of rapamycin
mTORC2	Mechanistic target of rapamycin complex 2
mtTFA	Mitochondrial transcription factor A
NBS1	Nijmegen breakage syndrome 1 (Nibrin)
NF-κB	Nuclear factor kappa-light-chain-enhancer of activated B cells
NGS	Next-generation sequencing
NHEJ	Non-homologous end joining
NK	Natural killer cell
NKG2D	Natural killer group 2 member D
NKP30	Natural killer cell p30 receptor
NKP44	Natural killer cell p44 receptor
NKP46	Natural killer cell p46 receptor
NMU	Neuromedin U
NOD/SCID	Non-obese diabetic/severe combined immunodeficiency
NOP58	Nucleolar protein 58
NOXA	Phorbol-12-myristate-13-acetate-induced protein 1
NPC	Nasopharyngeal carcinoma
NRAS	Neuroblastoma RAS viral oncogene homolog
NSCLC	Non-small cell lung cancer
NSG	NOD scid gamma immunodeficient mouse
O/ICW	On/in cell quantitative western assay
OC	Ovarian cancer
OS	Overall survival
P-AscH^−^	Pharmacological ascorbate
P-gp	P-glycoprotein
P21CIP1/WAF1	Cyclin-dependent kinase inhibitor 1A
PAK	p21-activated kinase
PARP	Poly(ADP-ribose) polymerase
PBPK	Physiologically based pharmacokinetic
PCa	Prostate cancer
PD	Pharmacodynamic
PD-1	Programmed cell death protein 1
PD-L1	Programmed death-ligand 1
PDAC	Pancreatic ductal adenocarcinoma
PDK1	Phosphoinositide-dependent kinase 1
PDK2	Phosphoinositide-dependent kinase 2
PDX	Patient-derived xenograft
PET	Positron emission tomography
PFS	Progression-free survival
pHGG	Pediatric high-grade glioma
PHH3	Phospho-histone H3
PI3K	Phosphoinositide 3-kinase
PIKK	Phosphoinositide 3-kinase-related kinase
PINK-1	PTEN-induced putative kinase 1
PK	Pharmacokinetic
PKB	Protein kinase B
PLK4	Polo-like kinase 4
POLH/Polη	DNA polymerase eta
POLIII	RNA polymerase III
POLQ	DNA polymerase theta
pRB	Retinoblastoma protein (phosphorylated)
PTEN	Phosphatase and tensin homolog
PUMA	p53 upregulated modulator of apoptosis
PUVA	Psoralen plus ultraviolet A
PVR	Poliovirus receptor
RAD18	E3 ubiquitin-protein ligase RAD18
RAD50	RAD50 double-strand break repair protein
RAD51	DNA repair protein RAD51 homolog 1
RAD51B	RAD51 paralog B
RAF	Rapidly accelerated fibrosarcoma
RANO	Response Assessment in Neuro-Oncology
RB1	Retinoblastoma protein 1
RBE	Relative biological effectiveness
RECIST	Response Evaluation Criteria in Solid Tumors
RIG-I	Retinoic acid-inducible gene I
RNA-seq	RNA sequencing
RNF168	RING finger protein 168
RNF4	RING finger protein 4
RNF8	RING finger protein 8
RNR/RR	Ribonucleotide reductase
ROS	Reactive oxygen species
RP2D	Recommended phase 2 dose
RPA	Replication protein A
RPA2	Replication protein A2
RPS23	Ribosomal protein S23
RRM1	Ribonucleotide reductase regulatory subunit M1
RRM2	Ribonucleotide reductase regulatory subunit M2
RS	Replication stress
RT	Radiotherapy
RT-qPCR	Reverse transcription quantitative polymerase chain reaction
SASP	Senescence-associated secretory phenotype
SBRT	Stereotactic body radiation therapy
SCLC	Small cell lung cancer
SER	Sensitizer enhancement ratio
shRNA	Short hairpin RNA
siRNA	Small interfering RNA
SLFN11	Schlafen family member 11
SMC1	Structural maintenance of chromosomes protein 1
SN38	Active metabolite of irinotecan
SNAIL	Snail family transcriptional repressor 1
SOC	Standard of care
STAG2	Stromal antigen 2 (cohesin subunit)
STAT3	Signal transducer and activator of transcription 3
STING	Stimulator of interferon genes
STS	Soft tissue sarcoma
SWI/SNF	SWItch/Sucrose Non-Fermentable
T1IFN	Type I interferon
TBK1	TANK-binding kinase 1
TCGA	The Cancer Genome Atlas
TEAE	Treatment-emergent adverse event
TGF-β	Transforming growth factor beta
TIP60	Tat-interactive protein, 60 kDa
TLS	Translesion synthesis
TMEM176A	Transmembrane protein 176A
TMZ	Temozolomide
TNBC	Triple-negative breast cancer
TNF-α	Tumor necrosis factor alpha
TOP(1/II)	Topoisomerase (I/II)
TOP2β	DNA topoisomerase II beta
TP53	Tumor protein p53
TRAIL	Tumor necrosis factor-related apoptosis-inducing ligand
TRXR1	Thioredoxin reductase 1
ULBPs	UL16-binding proteins
ULK1	Unc-51-like autophagy activating kinase 1
VEGFR	Vascular endothelial growth factor receptor
VHL	Von Hippel–Lindau
VP-16	Etoposide
WBRT	Whole-brain radiotherapy
WEE1	WEE1 G2 checkpoint kinase
WHO	World Health Organization
WRN	Werner syndrome helicase
WT	Wild-type
XRCC4	X-ray repair cross-complementing protein 4

## References

[1] Lavin MF , Yeo AJ . Clinical potential of ATM inhibitors. Mutat Res Fundam Mol Mech Mutagen. 2020; 821: 111695. doi:10.1016/j.mrfmmm.2020.111695. 32304909

[2] Beamish H , Williams R , Chen P , Lavin MF . Defect in multiple cell cycle checkpoints in *Ataxia*-telangiectasia postirradiation. J Biol Chem. 1996; 271( 34): 20486– 93. doi:10.1074/jbc.271.34.20486. 8702789

[3] Xu Y , Baltimore D . Dual roles of ATM in the cellular response to radiation and in cell growth control. Genes Dev. 1996; 10( 19): 2401– 10. doi:10.1101/gad.10.19.2401. 8843193

[4] Jung M , Kondratyev A , Lee SA , Dimtchev A , Dritschilo A . ATM gene product phosphorylates I kappa B-alpha. Cancer Res. 1997; 57( 1): 24– 7. 8988033

[5] Rathmell WK , Kaufmann WK , Hurt JC , Byrd LL , Chu G . DNA-dependent protein kinase is not required for accumulation of p53 or cell cycle arrest after DNA damage. Cancer Res. 1997; 57( 1): 68– 74. 8988043

[6] Piret B , Schoonbroodt S , Piette J . The ATM protein is required for sustained activation of NF-kappaB following DNA damage. Oncogene. 1999; 18( 13): 2261– 71. doi:10.1038/sj.onc.1202541. 10327072

[7] Nakagawa K , Taya Y , Tamai K , Yamaizumi M . Requirement of ATM in phosphorylation of the human p53 protein at serine 15 following DNA double-strand breaks. Mol Cell Biol. 1999; 19( 4): 2828– 34. doi:10.1128/MCB.19.4.2828. PMC84075 10082548

[8] Westphal CH , Schmaltz C , Rowan S , Elson A , Fisher DE , Leder P . Genetic interactions between atm and p53 influence cellular proliferation and irradiation-induced cell cycle checkpoints. Cancer Res. 1997; 57( 9): 1664– 7. 9135004

[9] Chen J . *Ataxia* Telangiectasia-related protein is involved in the phosphorylation of BRCA1 following deoxyribonucleic acid damage. Cancer Res. 2000; 60( 18): 5037– 9. 11016625

[10] Tibbetts RS , Cortez D , Brumbaugh KM , Scully R , Livingston D , Elledge SJ , et al. Functional interactions between BRCA1 and the checkpoint kinase ATR during genotoxic stress. Genes Dev. 2000; 14( 23): 2989– 3002. doi:10.1101/gad.851000. PMC317107 11114888

[11] Zhang J , Willers H , Feng Z , Ghosh JC , Kim S , Weaver DT , et al. Chk2 phosphorylation of BRCA1 regulates DNA double-strand break repair. Mol Cell Biol. 2004; 24( 2): 708– 18. doi:10.1128/MCB.24.2.708-718.2004. PMC343805 14701743

[12] Walker M , Black EJ , Oehler V , Gillespie DA , Scott MT . Chk1 C-terminal regulatory phosphorylation mediates checkpoint activation by de-repression of Chk1 catalytic activity. Oncogene. 2009; 28( 24): 2314– 23. doi:10.1038/onc.2009.102. PMC2857325 19421147

[13] Wilsker D , Bunz F . Chk1 phosphorylation during mitosis: a new role for a master regulator. Cell Cycle. 2009; 8( 8): 1161– 3. doi:10.4161/cc.8.8.8148. PMC2774453 19282670

[14] Kulkarni A , Das KC . Differential roles of ATR and ATM in p53, Chk1, and histone H2AX phosphorylation in response to hyperoxia: ATR-dependent ATM activation. Am J Physiol Lung Cell Mol Physiol. 2008; 294( 5): L998– 1006. doi:10.1152/ajplung.00004.2008. PMC8786037 18344416

[15] Xu X , Tsvetkov LM , Stern DF . Chk2 activation and phosphorylation-dependent oligomerization. Mol Cell Biol. 2002; 22( 12): 4419– 32. doi:10.1128/MCB.22.12.4419-4432.2002. PMC133858 12024051

[16] Burma S , Chen BP , Murphy M , Kurimasa A , Chen DJ . ATM phosphorylates histone H2AX in response to DNA double-strand breaks. J Biol Chem. 2001; 276( 45): 42462– 7. doi:10.1074/jbc.C100466200. 11571274

[17] Kobayashi J , Tauchi H , Chen B , Bruma S , Tashiro S , Matsuura S , et al. Histone H2AX participates the DNA damage-induced ATM activation through interaction with NBS1. Biochem Biophys Res Commun. 2009; 380( 4): 752– 7. doi:10.1016/j.bbrc.2009.01.109. 19338747

[18] Stiff T , O’Driscoll M , Rief N , Iwabuchi K , Löbrich M , Jeggo PA . ATM and DNA-PK function redundantly to phosphorylate H2AX after exposure to ionizing radiation. Cancer Res. 2004; 64( 7): 2390– 6. doi:10.1158/0008-5472.CAN-03-3207. 15059890

[19] Tanaka T , Halicka HD , Huang X , Traganos F , Darzynkiewicz Z . Constitutive histone H2AX phosphorylation and ATM activation, the reporters of DNA damage by endogenous oxidants. Cell Cycle. 2006; 5( 17): 1940– 5. doi:10.4161/cc.5.17.3191. PMC3488278 16940754

[20] McManus KJ , Hendzel MJ . ATM-dependent DNA damage-independent mitotic phosphorylation of H2AX in normally growing mammalian cells. Mol Biol Cell. 2005; 16( 10): 5013– 25. doi:10.1091/mbc.e05-01-0065. PMC1237100 16030261

[21] Harding SM , Coackley C , Bristow RG . ATM-dependent phosphorylation of 53BP1 in response to genomic stress in oxic and hypoxic cells. Radiother Oncol. 2011; 99( 3): 307– 12. doi:10.1016/j.radonc.2011.05.039. 21680038

[22] Lee H , Kwak HJ , Cho IT , Park SH , Lee CH . S1219 residue of 53BP1 is phosphorylated by ATM kinase upon DNA damage and required for proper execution of DNA damage response. Biochem Biophys Res Commun. 2009; 378( 1): 32– 6. doi:10.1016/j.bbrc.2008.10.150. 18996087

[23] Jungmichel S , Clapperton JA , Lloyd J , Hari FJ , Spycher C , Pavic L , et al. The molecular basis of ATM-dependent dimerization of the Mdc1 DNA damage checkpoint mediator. Nucleic Acids Res. 2012; 40( 9): 3913– 28. doi:10.1093/nar/gkr1300. PMC3351161 22234878

[24] Mochan TA , Venere M , DiTullio RA Jr , Halazonetis TD . 53BP1 and NFBD1/MDC1-Nbs1 function in parallel interacting pathways activating *Ataxia*-telangiectasia mutated (ATM) in response to DNA damage. Cancer Res. 2003; 63( 24): 8586– 91. 14695167

[25] Spycher C , Miller ES , Townsend K , Pavic L , Morrice NA , Janscak P , et al. Constitutive phosphorylation of MDC1 physically links the MRE11-RAD50-NBS1 complex to damaged chromatin. J Cell Biol. 2008; 181( 2): 227– 40. doi:10.1083/jcb.200709008. PMC2315671 18411308

[26] Wang H , Guan J , Wang H , Perrault AR , Wang Y , Iliakis G . Replication protein A2 phosphorylation after DNA damage by the coordinated action of *Ataxia* Telangiectasia-mutated and DNA-dependent protein kinase. Cancer Res. 2001; 61( 23): 8554– 63. 11731442

[27] Plug AW , Peters AH , Xu Y , Keegan KS , Hoekstra MF , Baltimore D , et al. ATM and RPA in meiotic chromosome *Synapsis* and recombination. Nat Genet. 1997; 17( 4): 457– 61. doi:10.1038/ng1297-457. 9398850

[28] Wang H , Wang M , Wang H , Böcker W , Iliakis G . Complex H2AX phosphorylation patterns by multiple kinases including ATM and DNA-PK in human cells exposed to ionizing radiation and treated with kinase inhibitors. J Cell Physiol. 2005; 202( 2): 492– 502. doi:10.1002/jcp.20141. 15389585

[29] Shiotani B , Zou L . Single-stranded DNA orchestrates an ATM-to-ATR switch at DNA breaks. Mol Cell. 2009; 33( 5): 547– 58. doi:10.1016/j.molcel.2009.01.024. PMC2675165 19285939

[30] Chanoux RA , Yin B , Urtishak KA , Asare A , Bassing CH , Brown EJ . ATR and H2AX cooperate in maintaining genome stability under replication stress. J Biol Chem. 2009; 284( 9): 5994– 6003. doi:10.1074/jbc.M806739200. PMC2645842 19049966

[31] Kim GD , Choi YH , Dimtchev A , Jeong SJ , Dritschilo A , Jung M . Sensing of ionizing radiation-induced DNA damage by ATM through interaction with histone deacetylase. J Biol Chem. 1999; 274( 44): 31127– 30. doi:10.1074/jbc.274.44.31127. 10531300

[32] Penicud K , Behrens A . DMAP1 is an essential regulator of ATM activity and function. Oncogene. 2014; 33( 4): 525– 31. doi:10.1038/onc.2012.597. PMC4255061 23318425

[33] Kim YC , Gerlitz G , Furusawa T , Catez F , Nussenzweig A , Oh KS , et al. Activation of ATM depends on chromatin interactions occurring before induction of DNA damage. Nat Cell Biol. 2009; 11( 1): 92– 6. doi:10.1038/ncb1817. PMC2717731 19079244

[34] Smith RJ , Savoian MS , Weber LE , Park JH . *Ataxia* Telangiectasia mutated (ATM) interacts with p400 ATPase for an efficient DNA damage response. BMC Mol Biol. 2016; 17( 1): 22. doi:10.1186/s12867-016-0075-7. PMC5097431 27814680

[35] Tyteca S , Vandromme M , Legube G , Chevillard-Briet M , Trouche D . Tip60 and p400 are both required for UV-induced apoptosis but play antagonistic roles in cell cycle progression. EMBO J. 2006; 25( 8): 1680– 9. doi:10.1038/sj.emboj.7601066. PMC1440826 16601686

[36] Dutto I , Scalera C , Prosperi E . CREBBP and p300 lysine acetyl transferases in the DNA damage response. Cell Mol Life Sci. 2018; 75( 8): 1325– 38. doi:10.1007/s00018-017-2717-4. PMC11105205 29170789

[37] Squatrito M , Gorrini C , Amati B . Tip60 in DNA damage response and growth control: many tricks in one HAT. Trends Cell Biol. 2006; 16( 9): 433– 42. doi:10.1016/j.tcb.2006.07.007. 16904321

[38] Sun Y , Jiang X , Price BD . Tip60: connecting chromatin to DNA damage signaling. Cell Cycle. 2010; 9( 5): 930– 6. doi:10.4161/cc.9.5.10931. PMC2901859 20160506

[39] Sardar S , McNair CM , Ravindranath L , Chand SN , Yuan W , Bogdan D , et al. AR coactivators, CBP/p300, are critical mediators of DNA repair in prostate cancer. Oncogene. 2024; 43( 43): 3197– 213. doi:10.1038/s41388-024-03148-4. PMC11493679 39266679

[40] Ogiwara H , Ui A , Otsuka A , Satoh H , Yokomi I , Nakajima S , et al. Histone acetylation by CBP and p300 at double-strand break sites facilitates SWI/SNF chromatin remodeling and the recruitment of non-homologous end joining factors. Oncogene. 2011; 30( 18): 2135– 46. doi:10.1038/onc.2010.592. 21217779

[41] Harrod A , Lane KA , Downs JA . The role of the SWI/SNF chromatin remodelling complex in the response to DNA double strand breaks. DNA Repair. 2020; 93: 102919. doi:10.1016/j.dnarep.2020.102919. 33087260

[42] Sadek M , Sheth A , Zimmerman G , Hays E , Vélez-Cruz R . The role of SWI/SNF chromatin remodelers in the repair of DNA double strand breaks and cancer therapy. Front Cell Dev Biol. 2022; 10: 1071786. doi:10.3389/fcell.2022.1071786. PMC9810387 36605718

[43] Ribeiro-Silva C , Vermeulen W , Lans H . SWI/SNF: complex complexes in genome stability and cancer. DNA Repair. 2019; 77: 87– 95. doi:10.1016/j.dnarep.2019.03.007. 30897376

[44] Mohan C , Das C , Tyler J . Histone and chromatin dynamics facilitating DNA repair. DNA Repair. 2021; 107: 103183. doi:10.1016/j.dnarep.2021.103183. PMC9733910 34419698

[45] Dinant C , Houtsmuller AB , Vermeulen W . Chromatin structure and DNA damage repair. Epigenet Chromatin. 2008; 1( 1): 9. doi:10.1186/1756-8935-1-9. PMC2596136 19014481

[46] Downs JA , Gasser SM . Chromatin remodeling and spatial concerns in DNA double-strand break repair. Curr Opin Cell Biol. 2024; 90: 102405. doi:10.1016/j.ceb.2024.102405. 39083951

[47] Foray N , Marot D , Gabriel A , Randrianarison V , Carr AM , Perricaudet M , et al. A subset of ATM-and ATR-dependent phosphorylation events requires the BRCA1 protein. EMBO J. 2003; 22( 11): 2860– 71. doi:10.1093/emboj/cdg274. PMC156770 12773400

[48] Yarden RI , Papa MZ . BRCA1 at the crossroad of multiple cellular pathways: approaches for therapeutic interventions. Mol Cancer Ther. 2006; 5( 6): 1396– 404. doi:10.1158/1535-7163.MCT-05-0471. 16818497

[49] Kehn K , Berro R , Alhaj A , Bottazzi ME , Yeh WI , Klase Z , et al. Functional consequences of cyclin D1/BRCA1 interaction in breast cancer cells. Oncogene. 2007; 26( 35): 5060– 9. doi:10.1038/sj.onc.1210319. 17334399

[50] Kelliher J , Ghosal G , Leung JWC . New answers to the old RIDDLE: RNF168 and the DNA damage response pathway. FEBS J. 2022; 289( 9): 2467– 80. doi:10.1111/febs.15857. PMC8486888 33797206

[51] Luo K , Deng M , Li Y , Wu C , Xu Z , Yuan J , et al. CDK-mediated RNF4 phosphorylation regulates homologous recombination in S-phase. Nucleic Acids Res. 2015; 43( 11): 5465– 75. doi:10.1093/nar/gkv434. PMC4477664 25948581

[52] Zhao X , Wei C , Li J , Xing P , Li J , Zheng S , et al. Cell cycle-dependent control of homologous recombination. Acta Biochim Biophys Sin. 2017; 49( 8): 655– 68. doi:10.1093/abbs/gmx055. 28541389

[53] Kato TA , Maeda J , Watanabe H , Kawamura S , Wilson PF . Simultaneous inhibition of ATM, ATR, and DNA-PK causes synergistic lethality. Biochem Biophys Res Commun. 2024; 738: 150517. doi:10.1016/j.bbrc.2024.150517. 39146620

[54] Ali R , Alblihy A , Toss MS , Algethami M , Al Sunni R , Green AR , et al. XRCC1 deficient triple negative breast cancers are sensitive to ATR, ATM and Wee1 inhibitor either alone or in combination with olaparib. Ther Adv Med Oncol. 2020; 12: 1758835920974201. doi:10.1177/1758835920974201. PMC7758562 33425022

[55] Tozaki Y , Aoki H , Kato R , Toriuchi K , Arame S , Inoue Y , et al. The combination of ATM and Chk1 inhibitors induces synthetic lethality in colorectal cancer cells. Cancers. 2023; 15( 3): 735. doi:10.3390/cancers15030735. PMC9913148 36765693

[56] Li J , Zhang L , Shang Y , Liu J , Zhao H . Synthetic lethality in cancer therapy: mechanisms, models and clinical translation for overcoming therapeutic resistance. Clin Transl Med. 2026; 16( 1): e70586. doi:10.1002/ctm2.70586. PMC12805533 41537447

[57] Carrassa L , Damia G . DNA damage response inhibitors: mechanisms and potential applications in cancer therapy. Cancer Treat Rev. 2017; 60: 139– 51. doi:10.1016/j.ctrv.2017.08.013. 28961555

[58] Jackson SP , Bartek J . The DNA-damage response in human biology and disease. Nature. 2009; 461( 7267): 1071– 8. doi:10.1038/nature08467. PMC2906700 19847258

[59] Negrini S , Gorgoulis VG , Halazonetis TD . Genomic instability—an evolving hallmark of cancer. Nat Rev Mol Cell Biol. 2010; 11( 3): 220– 8. doi:10.1038/nrm2858. 20177397

[60] Zeman MK , Cimprich KA . Causes and consequences of replication stress. Nat Cell Biol. 2014; 16( 1): 2– 9. doi:10.1038/ncb2897. PMC4354890 24366029

[61] Ronco C , Martin AR , Demange L , Benhida R . ATM, ATR, CHK1, CHK2 and WEE1 inhibitors in cancer and cancer stem cells. Med Chem Commun. 2017; 8( 2): 295– 319. doi:10.1039/C6MD00439C. PMC6072143 30108746

[62] Sayed U , Abdulsahib WK , Jasim IK , Malathi H , Nayak PP , Alex Anand D , et al. Synthetic lethality and DNA damage response targeting in cancer stem cells: a comprehensive review. Discov Oncol. 2026; 17( 1): 449. doi:10.1007/s12672-026-04587-z. PMC13004795 41686300

[63] Ahmed KM , Li JJ . ATM-NF-kappaB connection as a target for tumor radiosensitization. Curr Cancer Drug Targets. 2007; 7( 4): 335– 42. doi:10.2174/156800907780809769. PMC4156318 17979628

[64] Jung M , Dritschilo A . NF-kappa B signaling pathway as a target for human tumor radiosensitization. Semin Radiat Oncol. 2001; 11( 4): 346– 51. doi:10.1053/srao.2001.26034. 11677659

[65] Panta GR , Kaur S , Cavin LG , Cortés ML , Mercurio F , Lothstein L , et al. ATM and the catalytic subunit of DNA-dependent protein kinase activate NF-kappaB through a common MEK/extracellular signal-regulated kinase/p90(rsk) signaling pathway in response to distinct forms of DNA damage. Mol Cell Biol. 2004; 24( 5): 1823– 35. doi:10.1128/MCB.24.5.1823-1835.2004. PMC350545 14966265

[66] Shen M , Xu Z , Xu W , Jiang K , Zhang F , Ding Q , et al. Inhibition of ATM reverses EMT and decreases metastatic potential of cisplatin-resistant lung cancer cells through JAK/STAT3/PD-L1 pathway. J Exp Clin Cancer Res. 2019; 38( 1): 149. doi:10.1186/s13046-019-1161-8. PMC6454747 30961670

[67] Bai M , Xu P , Cheng R , Li N , Cao S , Guo Q , et al. ROS-ATM-CHK2 axis stabilizes HIF-1α and promotes tumor angiogenesis in hypoxic microenvironment. Oncogene. 2025; 44( 21): 1609– 19. doi:10.1038/s41388-025-03336-w. PMC12095048 40057605

[68] Sun K , Tang S , Hou Y , Xi L , Chen Y , Yin J , et al. Oxidized ATM-mediated glycolysis enhancement in breast cancer-associated fibroblasts contributes to tumor invasion through lactate as metabolic coupling. EBioMedicine. 2019; 41: 370– 83. doi:10.1016/j.ebiom.2019.02.025. PMC6442874 30799198

[69] Yamamoto Y , Hosoda K , Imahori T , Tanaka J , Matsuo K , Nakai T , et al. Pentose phosphate pathway activation via HSP27 phosphorylation by ATM kinase: a putative endogenous antioxidant defense mechanism during cerebral ischemia-reperfusion. Brain Res. 2018; 1687: 82– 94. doi:10.1016/j.brainres.2018.03.001. 29510140

[70] Cosentino C , Grieco D , Costanzo V . ATM activates the pentose phosphate pathway promoting anti-oxidant defence and DNA repair. EMBO J. 2011; 30( 3): 546– 55. doi:10.1038/emboj.2010.330. PMC3034007 21157431

[71] Lee JH . ATM in immunobiology: from lymphocyte development to cancer immunotherapy. Transl Oncol. 2025; 52: 102268. doi:10.1016/j.tranon.2024.102268. PMC11754496 39752906

[72] Xu P , Sun Y , Song Y , Jiao J , Shen B , Li W , et al. ATM kinase regulates tumor immunoreactions in lymphocyte-predominant breast cancer through modulation of NKG2D ligand and TNF cytokines on tumor cells. Med Mol Morphol. 2020; 53( 4): 210– 20. doi:10.1007/s00795-020-00247-5. 32067111

[73] Jenni R , Chikhaoui A , Nabouli I , Zaouak A , Khanchel F , Hammami-Ghorbel H , et al. Differential expression of ATM, NF-KB, PINK1 and Foxo3a in radiation-induced basal cell carcinoma. Int J Mol Sci. 2023; 24( 8): 7181. doi:10.3390/ijms24087181. PMC10138907 37108343

[74] Kaelin WG Jr . The concept of synthetic lethality in the context of anticancer therapy. Nat Rev Cancer. 2005; 5( 9): 689– 98. doi:10.1038/nrc1691. 16110319

[75] Ashworth A , Lord CJ . Synthetic lethal therapies for cancer: what’s next after PARP inhibitors? Nat Rev Clin Oncol. 2018; 15( 9): 564– 76. doi:10.1038/s41571-018-0055-6. 29955114

[76] Kantidze OL , Velichko AK , Luzhin AV , Petrova NV , Razin SV . Synthetically lethal interactions of ATM, ATR, and DNA-PKcs. Trends Cancer. 2018; 4( 11): 755– 68. doi:10.1016/j.trecan.2018.09.007. 30352678

[77] Chen CC , Kass EM , Yen WF , Ludwig T , Moynahan ME , Chaudhuri J , et al. ATM loss leads to synthetic lethality in BRCA1 BRCT mutant mice associated with exacerbated defects in homology-directed repair. Proc Natl Acad Sci U S A. 2017; 114( 29): 7665– 70. doi:10.1073/pnas.1706392114. PMC5530697 28659469

[78] Muvaffak A , Coleman KG . PARP inhibitor synthetic lethality in ATM biallelic mutant cancer cell lines is associated with BRCA1/2 and RAD51 downregulation. Front Oncol. 2024; 14: 1380633. doi:10.3389/fonc.2024.1380633. PMC11131418 38807759

[79] Yang Y , Liu Q , Li X , Zhang H , Xu X , Ma Q , et al. Targeting the ATM-TRMT10A-BRCA1 axis confers synthetic lethality to PARP inhibition in metastatic castration-resistant prostate cancer. Sci Adv. 2025; 11( 41): eadw7989. doi:10.1126/sciadv.adw7989. PMC12513462 41071892

[80] Folk WP , Kumari A , Iwasaki T , Cassimere EK , Pyndiah S , Martin E , et al. New synthetic lethality re-sensitizing platinum-refractory cancer cells to cisplatin *in vitro*: the rationale to co-use PARP and ATM inhibitors. Int J Mol Sci. 2021; 22( 24): 13324. doi:10.3390/ijms222413324. PMC8704450 34948122

[81] Menezes DL , Holt J , Tang Y , Feng J , Barsanti P , Pan Y , et al. A synthetic lethal screen reveals enhanced sensitivity to ATR inhibitor treatment in mantle cell lymphoma with ATM loss-of-function. Mol Cancer Res. 2015; 13( 1): 120– 9. doi:10.1158/1541-7786.MCR-14-0240. 25232030

[82] Min A , Im SA , Jang H , Kim S , Lee M , Kim DK , et al. AZD6738, a novel oral inhibitor of ATR, induces synthetic lethality with ATM deficiency in gastric cancer cells. Mol Cancer Ther. 2017; 16( 4): 566– 77. doi:10.1158/1535-7163.MCT-16-0378. 28138034

[83] Lebeau A , Kakkos A , Leroi N , Bours V , Delbecque K , Goffin F , et al. Targeting synthetic lethality: an effective therapeutic approach in ovarian and endometrial cancers. Ther Adv Med Oncol. 2025; 17: 17588359251392106. doi:10.1177/17588359251392106. PMC12690063 41383404

[84] McCabe N , Walker SM , Kennedy RD . When the guardian becomes the enemy: targeting ATM in PTEN-deficient cancers. Mol Cell Oncol. 2015; 3( 1): e1053595. doi:10.1080/23723556.2015.1053595. PMC4845205 27308567

[85] Zimmermann A , Zenke FT , Chiu LY , Dahmen H , Pehl U , Fuchss T , et al. A new class of selective ATM inhibitors as combination partners of DNA double-strand break inducing cancer therapies. Mol Cancer Ther. 2022; 21( 6): 859– 70. doi:10.1158/1535-7163.MCT-21-0934. PMC9381122 35405736

[86] Rainey MD , Charlton ME , Stanton RV , Kastan MB . Transient inhibition of ATM kinase is sufficient to enhance cellular sensitivity to ionizing radiation. Cancer Res. 2008; 68( 18): 7466– 74. doi:10.1158/0008-5472.CAN-08-0763. PMC2559948 18794134

[87] Shu J , Wang X , Yang X , Zhao G . ATM inhibitor KU60019 synergistically sensitizes lung cancer cells to topoisomerase II poisons by multiple mechanisms. Sci Rep. 2023; 13: 882. doi:10.1038/s41598-023-28185-z. PMC9845372 36650267

[88] Li K , Yan H , Guo W , Tang M , Zhao X , Tong A , et al. ATM inhibition induces synthetic lethality and enhances sensitivity of PTEN-deficient breast cancer cells to cisplatin. Exp Cell Res. 2018; 366( 1): 24– 33. doi:10.1016/j.yexcr.2018.03.006. 29522753

[89] Özdemir D , Ali Ağca C . AZD1390, an *Ataxia* Telangiectasia mutated inhibitor, enhances cisplatin mediated apoptosis in breast cancer cells. Exp Cell Res. 2025; 444( 2): 114382. doi:10.1016/j.yexcr.2024.114382. 39681282

[90] Bakkenist CJ , Lee JJ , Schmitz JC . ATM is required for the repair of oxaliplatin-induced DNA damage in colorectal cancer. Clin Colorectal Cancer. 2018; 17( 4): 255– 7. doi:10.1016/j.clcc.2018.09.001. PMC6561466 30316683

[91] Choi S , Gamper AM , White JS , Bakkenist CJ . Inhibition of ATM kinase activity does not phenocopy ATM protein disruption: implications for the clinical utility of ATM kinase inhibitors. Cell Cycle. 2010; 9( 20): 4052– 7. doi:10.4161/cc.9.20.13471. PMC3230471 20953138

[92] Menolfi D , Zha S . ATM, ATR and DNA-PKcs kinases—the lessons from the mouse models: inhibition?≠?deletion. Cell Biosci. 2020; 10( 1): 8. doi:10.1186/s13578-020-0376-x. PMC6990542 32015826

[93] Xie J , Kuriakose T , Bianski B , Twarog N , Savage E , Xu K , et al. ATM inhibition enhances the efficacy of radiation across distinct molecular subgroups of pediatric high-grade glioma. Neuro Oncol. 2023; 25( 10): 1828– 41. doi:10.1093/neuonc/noad064. PMC10547515 36971093

[94] Sarkaria JN , Eshleman JS . ATM as a target for novel radiosensitizers. Semin Radiat Oncol. 2001; 11( 4): 316– 27. doi:10.1053/srao.2001.26030. 11677656

[95] Ho AY , Fan G , Atencio DP , Green S , Formenti SC , Haffty BG , et al. Possession of ATM sequence variants as predictor for late normal tissue responses in breast cancer patients treated with radiotherapy. Int J Radiat Oncol Biol Phys. 2007; 69( 3): 677– 84. doi:10.1016/j.ijrobp.2007.04.012. 17517479

[96] Tew BY , Kalfa AJ , Yang Z , Hurth KM , Simon T , Abnoosian E , et al. ATM-inhibitor AZD1390 is a radiosensitizer for breast cancer CNS metastasis. Clin Cancer Res. 2023; 29( 21): 4492– 503. doi:10.1158/1078-0432.CCR-23-0290. PMC10618650 37585496

[97] Torok JA , Oh P , Castle KD , Reinsvold M , Ma Y , Luo L , et al. Deletion of atm in tumor but not endothelial cells improves radiation response in a primary mouse model of lung adenocarcinoma. Cancer Res. 2019; 79( 4): 773– 82. doi:10.1158/0008-5472.CAN-17-3103. PMC6377832 30315114

[98] Meidenbauer J , Wachter M , Schulz SR , Mostafa N , Zülch L , Frey B , et al. Inhibition of ATM or ATR in combination with hypo-fractionated radiotherapy leads to a different immunophenotype on transcript and protein level in HNSCC. Front Oncol. 2024; 14: 1460150. doi:10.3389/fonc.2024.1460150. PMC11473424 39411143

[99] Fabbrizi MR , Doggett TJ , Hughes JR , Melia E , Dufficy ER , Hill RM , et al. Inhibition of key DNA double strand break repair protein kinases enhances radiosensitivity of head and neck cancer cells to X-ray and proton irradiation. Cell Death Discov. 2024; 10: 282. doi:10.1038/s41420-024-02059-3. PMC11169544 38866739

[100] Xue Z , Mladek AC , Rathi S , Jain S , Vaubel RA , Odukoya LA , et al. The novel brain penetrant *Ataxia*-telangiectasia mutated inhibitor WSD0628 provides robust radiosensitization of brain tumor patient-derived xenografts. Neuro Oncol. 2025; 27( 9): 2313– 25. doi:10.1093/neuonc/noaf102. PMC12526041 40230030

[101] Chiu LY , Sun Q , Zenke FT , Blaukat A , Vassilev LT . Selective ATM inhibition augments radiation-induced inflammatory signaling and cancer cell death. Aging. 2023; 15( 2): 492– 512. doi:10.18632/aging.204487. PMC9925676 36656721

[102] Fernandez A , Artola M , Leon S , Otegui N , Jimeno A , Serrano D , et al. Cancer vulnerabilities through targeting the ATR/Chk1 and ATM/Chk2 axes in the context of DNA damage. Cells. 2025; 14( 10): 748. doi:10.3390/cells14100748. PMC12110066 40422251

[103] Brunn GJ , Williams J , Sabers C , Wiederrecht G , Lawrence JC Jr , Abraham RT . Direct inhibition of the signaling functions of the mammalian target of rapamycin by the phosphoinositide 3-kinase inhibitors, wortmannin and LY294002. EMBO J. 1996; 15( 19): 5256– 67. doi:10.1002/j.1460-2075.1996.tb00911.x. PMC452270 8895571

[104] Ihara M , Shichijo K , Takeshita S , Kudo T . Wortmannin, a specific inhibitor of phosphatidylinositol-3-kinase, induces accumulation of DNA double-strand breaks. J Radiat Res. 2020; 61( 2): 171– 6. doi:10.1093/jrr/rrz102. PMC7246056 32052028

[105] Schultz RM , Merriman RL , Andis SL , Bonjouklian R , Grindey GB , Rutherford PG , et al. *In vitro* and *in vivo* antitumor activity of the phosphatidylinositol-3-kinase inhibitor, wortmannin. Anticancer Res. 1995; 15( 4): 1135– 9. 7653991

[106] Sarkaria JN , Busby EC , Tibbetts RS , Roos P , Taya Y , Karnitz LM , et al. Inhibition of ATM and ATR kinase activities by the radiosensitizing agent, caffeine. Cancer Res. 1999; 59( 17): 4375– 82. 10485486

[107] Blasina A , Price BD , Turenne GA , McGowan CH . Caffeine inhibits the checkpoint kinase ATM. Curr Biol. 1999; 9( 19): 1135– 8. doi:10.1016/S0960-9822(99)80486-2. 10531013

[108] Moliard L , Restier-Verlet J , Al-Choboq J , Granzotto A , Charlet L , Balosso J , et al. Caffeine may delay the radiation-induced nucleoshuttling of the ATM kinase and reduce the recognition of the DNA double-strand breaks in human cells. Biomolecules. 2025; 16( 1): 41. doi:10.3390/biom16010041. PMC12839020 41594582

[109] Alao JP , Sunnerhagen P . The ATM and ATR inhibitors CGK733 and caffeine suppress cyclin D1 levels and inhibit cell proliferation. Radiat Oncol. 2009; 4: 51. doi:10.1186/1748-717X-4-51. PMC2777912 19903334

[110] Vlahos CJ , Matter WF , Hui KY , Brown RF . A specific inhibitor of phosphatidylinositol 3-kinase, 2-(4-morpholinyl)-8-phenyl-4H-1-benzopyran-4-one (LY294002). J Biol Chem. 1994; 269( 7): 5241– 8. doi:10.1016/S0021-9258(17)37680-9. 8106507

[111] Hickson I , Zhao Y , Richardson CJ , Green SJ , Martin NMB , Orr AI , et al. Identification and characterization of a novel and specific inhibitor of the *Ataxia*-telangiectasia mutated kinase ATM. Cancer Res. 2004; 64( 24): 9152– 9. doi:10.1158/0008-5472.CAN-04-2727. 15604286

[112] Al-Zoubi RM , Garada K , Al Huneidi R , Baddar ZMH , Zarour AA , Elaarag M , et al. ATM inhibitors in cancer radiotherapy: mechanisms, clinical development, and future directions. Eur J Med Chem. 2025; 300: 118137. doi:10.1016/j.ejmech.2025.118137. 40945322

[113] Du S , Liang Q , Shi J . Progress of ATM inhibitors: opportunities and challenges. Eur J Med Chem. 2024; 277: 116781. doi:10.1016/j.ejmech.2024.116781. 39173286

[114] Ampolini EA , Jimenez-Sainz J , Long DT . The development of ATM inhibitors in cancer therapy. Target Oncol. 2025; 20( 2): 281– 97. doi:10.1007/s11523-025-01136-6. PMC11933189 40024979

[115] Dong C , Wang Y , Tu Z , Yan J , Jiang Y , Lu T , et al. Recent advances in ATM inhibitors as potential therapeutic agents. Future Med Chem. 2022; 14( 23): 1811– 30. doi:10.4155/fmc-2022-0252. 36484176

[116] Cowell IG , Durkacz BW , Tilby MJ . Sensitization of breast carcinoma cells to ionizing radiation by small molecule inhibitors of DNA-dependent protein kinase and *Ataxia* Telangiectsia mutated. Biochem Pharmacol. 2005; 71( 1–2): 13– 20. doi:10.1016/j.bcp.2005.09.029. 16293233

[117] Golding SE , Rosenberg E , Neill S , Dent P , Povirk LF , Valerie K . Extracellular signal-related kinase positively regulates *Ataxia* Telangiectasia mutated, homologous recombination repair, and the DNA damage response. Cancer Res. 2007; 67( 3): 1046– 53. doi:10.1158/0008-5472.CAN-06-2371. 17283137

[118] Kennedy RD , Chen CC , Stuckert P , Archila EM , De la Vega MA , Moreau LA , et al. Fanconi *Anemia* Pathway-deficient tumor cells are hypersensitive to inhibition of *Ataxia* Telangiectasia mutated. J Clin Investig. 2007; 117( 5): 1440– 9. doi:10.1172/JCI31245. PMC1847538 17431503

[119] Eaton JS , Lin ZP , Sartorelli AC , Bonawitz ND , Shadel GS . *Ataxia*-telangiectasia mutated kinase regulates ribonucleotide reductase and mitochondrial homeostasis. J Clin Investig. 2007; 117( 9): 2723– 34. doi:10.1172/JCI31604. PMC1952633 17786248

[120] Cruet-Hennequart S , Glynn MT , Murillo LS , Coyne S , Carty MP . Enhanced DNA-PK-mediated RPA2 hyperphosphorylation in DNA polymerase eta-deficient human cells treated with cisplatin and oxaliplatin. DNA Repair. 2008; 7( 4): 582– 96. doi:10.1016/j.dnarep.2007.12.012. 18289945

[121] Cheng WH , Muftic D , Muftuoglu M , Dawut L , Morris C , Helleday T , et al. WRN is required for ATM activation and the S-phase checkpoint in response to interstrand cross-link-induced DNA double-strand breaks. Mol Biol Cell. 2008; 19( 9): 3923– 33. doi:10.1091/mbc.e07-07-0698. PMC2526706 18596239

[122] Soriani A , Zingoni A , Cerboni C , Iannitto ML , Ricciardi MR , Di Gialleonardo V , et al. ATM-ATR–dependent up-regulation of DNAM-1 and NKG2D ligands on multiple myeloma cells by therapeutic agents results in enhanced NK-cell susceptibility and is associated with a senescent phenotype. Blood. 2009; 113( 15): 3503– 11. doi:10.1182/blood-2008-08-173914. 19098271

[123] Ahmed KM , Nantajit D , Fan M , Murley JS , Grdina DJ , Li JJ . Coactivation of ATM/ERK/NF-kappaB in the low-dose radiation-induced radioadaptive response in human skin keratinocytes. Free Radic Biol Med. 2009; 46( 11): 1543– 50. doi:10.1016/j.freeradbiomed.2009.03.012. PMC6759050 19324081

[124] Ivanov VN , Zhou H , Partridge MA , Hei TK . Inhibition of *Ataxia* Telangiectasia mutated kinase activity enhances TRAIL-mediated apoptosis in human melanoma cells. Cancer Res. 2009; 69( 8): 3510– 9. doi:10.1158/0008-5472.CAN-08-3883. PMC4070218 19351839

[125] Zhang YW , Jones TL , Martin SE , Caplen NJ , Pommier Y . Implication of checkpoint kinase-dependent up-regulation of ribonucleotide reductase R2 in DNA damage response. J Biol Chem. 2009; 284( 27): 18085– 95. doi:10.1074/jbc.M109.003020. PMC2709352 19416980

[126] Li Y , Yang DQ . The ATM inhibitor KU-55933 suppresses cell proliferation and induces apoptosis by blocking Akt in cancer cells with overactivated Akt. Mol Cancer Ther. 2010; 9( 1): 113– 25. doi:10.1158/1535-7163.MCT-08-1189. 20053781

[127] Pizarro JG , Folch J , de la Torre AV , Junyent F , Verdaguer E , Jordan J , et al. ATM is involved in cell-cycle control through the regulation of retinoblastoma protein phosphorylation. J Cell Biochem. 2010; 110( 1): 210– 8. doi:10.1002/jcb.22528. 20213763

[128] Xu N , Hegarat N , Black EJ , Scott MT , Hochegger H , Gillespie DA . Akt/PKB suppresses DNA damage processing and checkpoint activation in late G2. J Cell Biol. 2010; 190( 3): 297– 305. doi:10.1083/jcb.201003004. PMC2922641 20679434

[129] Qi Y , Schoene NW , Lartey FM , Cheng WH . Selenium compounds activate ATM-dependent DNA damage response via the mismatch repair protein hMLH1 in colorectal cancer cells. J Biol Chem. 2010; 285( 43): 33010– 7. doi:10.1074/jbc.M110.137406. PMC2963351 20709753

[130] Liontos M , Velimezi G , Pateras IS , Angelopoulou R , Papavassiliou AG , Bartek J , et al. The roles of p27(Kip1) and DNA damage signalling in the chemotherapy-induced delayed cell cycle checkpoint. J Cell Mol Med. 2010; 14( 9): 2264– 7. doi:10.1111/j.1582-4934.2010.01145.x. PMC3822566 20716117

[131] Pan Y , Yuan D , Zhang J , Shao C . Cadmium induced radioadaptive response via an ATM-independent H_2_S/cystathionine γ-lyase modulation. Mutat Res Fundam Mol Mech Mutagen. 2011; 707( 1–2): 67– 73. doi:10.1016/j.mrfmmm.2010.12.014. 21195095

[132] Lossaint G , Besnard E , Fisher D , Piette J , Dulić V . Chk1 is dispensable for G2 arrest in response to sustained DNA damage when the ATM/p53/p21 pathway is functional. Oncogene. 2011; 30( 41): 4261– 74. doi:10.1038/onc.2011.135. 21532626

[133] He L , Nan MH , Oh HC , Kim YH , Jang JH , Erikson RL , et al. Asperlin induces G_2_/M arrest through ROS generation and ATM pathway in human cervical carcinoma cells. Biochem Biophys Res Commun. 2011; 409( 3): 489– 93. doi:10.1016/j.bbrc.2011.05.032. 21600879

[134] Soleimani R , Heytens E , Darzynkiewicz Z , Oktay K . Mechanisms of chemotherapy-induced human ovarian aging: double strand DNA breaks and microvascular compromise. Aging. 2011; 3( 8): 782– 93. doi:10.18632/aging.100363. PMC3184979 21869459

[135] Raso A , Vecchio D , Cappelli E , Ropolo M , Poggi A , Nozza P , et al. Characterization of glioma stem cells through multiple stem cell markers and their specific sensitization to double-strand break-inducing agents by pharmacological inhibition of *Ataxia* Telangiectasia mutated protein. Brain Pathol. 2012; 22( 5): 677– 88. doi:10.1111/j.1750-3639.2012.00566.x. PMC8057648 22257080

[136] Moiola C , De Luca P , Cotignola J , Gardner K , Vazquez E , De Siervi A . Dynamic coregulatory complex containing BRCA1, E2F1 and CtIP controls ATM transcription. Cell Physiol Biochem. 2012; 30( 3): 596– 608. doi:10.1159/000341441. PMC7451964 22832221

[137] Nadkarni A , Shrivastav M , Mladek AC , Schwingler PM , Grogan PT , Chen J , et al. ATM inhibitor KU-55933 increases the TMZ responsiveness of only inherently TMZ sensitive GBM cells. J Neurooncol. 2012; 110( 3): 349– 57. doi:10.1007/s11060-012-0979-0. PMC3535329 23054561

[138] Sun H , Wang Y , Wang Z , Meng J , Qi Z , Yang G . Aurora-a controls cancer cell radio- and chemoresistance via ATM/Chk2-mediated DNA repair networks. Biochim Biophys Acta. 2014; 1843( 5): 934– 44. doi:10.1016/j.bbamcr.2014.01.019. 24480460

[139] Zou Y , Wang Q , Li B , Xie B , Wang W . Temozolomide induces autophagy via ATM-AMPK-ULK1 pathways in glioma. Mol Med Rep. 2014; 10( 1): 411– 6. doi:10.3892/mmr.2014.2151. 24737504

[140] Toulany M , Mihatsch J , Holler M , Chaachouay H , Rodemann HP . Cisplatin-mediated radiosensitization of non-small cell lung cancer cells is stimulated by ATM inhibition. Radiother Oncol. 2014; 111( 2): 228– 36. doi:10.1016/j.radonc.2014.04.001. 24857596

[141] Carruthers R , Ahmed SU , Strathdee K , Gomez-Roman N , Amoah-Buahin E , Watts C , et al. Abrogation of radioresistance in glioblastoma stem-like cells by inhibition of ATM kinase. Mol Oncol. 2015; 9( 1): 192– 203. doi:10.1016/j.molonc.2014.08.003. PMC5528679 25205037

[142] Teng PN , Bateman NW , Darcy KM , Hamilton CA , Maxwell GL , Bakkenist CJ , et al. Pharmacologic inhibition of ATR and ATM offers clinically important distinctions to enhancing platinum or radiation response in ovarian, endometrial, and cervical cancer cells. Gynecol Oncol. 2015; 136( 3): 554– 61. doi:10.1016/j.ygyno.2014.12.035. PMC4382918 25560806

[143] Zhang T , Shen Y , Chen Y , Hsieh JT , Kong Z . The *ATM* inhibitor KU55933 sensitizes radioresistant bladder cancer cells with DAB2IP gene defect. Int J Radiat Biol. 2015; 91( 4): 368– 78. doi:10.3109/09553002.2015.1001531. 25585815

[144] Spryszyńska S , Smok-Pieniążek A , Ferlińska M , Roszak J , Nocuń M , Stępnik M . The influence of ATM, ATR, DNA-PK inhibitors on the cytotoxic and genotoxic effects of dibenzo [def, p] chrysene on human hepatocellular cancer cell line HepG2. Mutat Res Genet Toxicol Environ Mutagen. 2015; 791: 12– 24. doi:10.1016/j.mrgentox.2015.07.008. 26338538

[145] Tian X , Lara H , Wagner KT , Saripalli S , Hyder SN , Foote M , et al. Improving DNA double-strand repair inhibitor KU55933 therapeutic index in cancer radiotherapy using nanoparticle drug delivery. Nanoscale. 2015; 7( 47): 20211– 9. doi:10.1039/C5NR05869D. PMC4664156 26575637

[146] Stagni V , Kaminari A , Contadini C , Barilà D , Sessa RL , Sideratou Z , et al. A triphenylphosphonium-functionalized delivery system for an ATM kinase inhibitor that ameliorates doxorubicin resistance in breast carcinoma mammospheres. Cancers. 2023; 15( 5): 1474. doi:10.3390/cancers15051474. PMC10000448 36900267

[147] Harris BRE , Zhang Y , Tao J , Shen R , Zhao X , Cleary MP , et al. ATM inhibitor KU-55933 induces apoptosis and inhibits motility by blocking GLUT1-mediated glucose uptake in aggressive cancer cells with sustained activation of Akt. FASEB J. 2021; 35( 4): e21264. doi:10.1096/fj.202001415RR. 33715230

[148] Wu T , Zhou S , Qin M , Tang J , Yan X , Huang L , et al. Phenformin and *Ataxia*-telangiectasia mutated inhibitors synergistically co-suppress liver cancer cell growth by damaging mitochondria. FEBS Open Bio. 2021; 11( 5): 1440– 51. doi:10.1002/2211-5463.13152. PMC8091576 33742560

[149] Liu K , Chen Y , Shi XB , Xing ZH , He ZJ , Wang ST , et al. Inhibiting the activity of ABCG2 by KU55933 in colorectal cancer. Recent Pat Anti Cancer Drug Discov. 2022; 17( 4): 387– 95. doi:10.2174/1574892817666220112100036. 35023460

[150] Zhang A , Zhang L , Xie X , Liu D . Inhibition of ATM with KU-55933 sensitizes endometrial cancer cell lines to olaparib. OncoTargets Ther. 2023; 16: 1061– 71. doi:10.2147/OTT.S426923. PMC10748556 38144904

[151] Misumi K , Sun J , Kinomura A , Miyata Y , Okada M , Tashiro S . Enhanced gefitinib-induced repression of the epidermal growth factor receptor pathway by *Ataxia* Telangiectasia-mutated kinase inhibition in non-small-cell lung cancer cells. Cancer Sci. 2016; 107( 4): 444– 51. doi:10.1111/cas.12899. PMC4832868 26825989

[152] Shen X , Xia Y , Lu H , Zheng P , Wang J , Chen Y , et al. Synergistic targeting of TrxR1 and ATM/AKT pathway in human colon cancer cells. Biomed Pharmacother. 2024; 174: 116507. doi:10.1016/j.biopha.2024.116507. 38565059

[153] Aiyappa-Maudsley R , Elsalem L , Ibrahim AIM , Pors K , Martin SG . *In vitro* radiosensitization of breast cancer with hypoxia-activated prodrugs. J Cell Mol Med. 2022; 26( 16): 4577– 90. doi:10.1111/jcmm.17486. PMC9357624 35841287

[154] Batey MA , Zhao Y , Kyle S , Richardson C , Slade A , Martin NMB , et al. Preclinical evaluation of a novel ATM inhibitor, KU59403, *in vitro* and *in vivo* in p53 functional and dysfunctional models of human cancer. Mol Cancer Ther. 2013; 12( 6): 959– 67. doi:10.1158/1535-7163.MCT-12-0707. PMC3736091 23512991

[155] Golding SE , Rosenberg E , Valerie N , Hussaini I , Frigerio M , Cockcroft XF , et al. Improved ATM kinase inhibitor KU-60019 radiosensitizes glioma cells, compromises insulin, AKT and ERK prosurvival signaling, and inhibits migration and invasion. Mol Cancer Ther. 2009; 8( 10): 2894– 902. doi:10.1158/1535-7163.MCT-09-0519. PMC2761990 19808981

[156] Golding SE , Rosenberg E , Adams BR , Wignarajah S , Beckta JM , O’Connor MJ , et al. Dynamic inhibition of ATM kinase provides a strategy for glioblastoma multiforme radiosensitization and growth control. Cell Cycle. 2012; 11( 6): 1167– 73. doi:10.4161/cc.11.6.19576. PMC3335919 22370485

[157] Biddlestone-Thorpe L , Sajjad M , Rosenberg E , Beckta JM , Valerie NCK , Tokarz M , et al. ATM kinase inhibition preferentially sensitizes p53-mutant glioma to ionizing radiation. Clin Cancer Res. 2013; 19( 12): 3189– 200. doi:10.1158/1078-0432.CCR-12-3408. PMC3687028 23620409

[158] Vecchio D , Daga A , Carra E , Marubbi D , Baio G , Neumaier CE , et al. Predictability, efficacy and safety of radiosensitization of glioblastoma-initiating cells by the ATM inhibitor KU-60019. Int J Cancer. 2014; 135( 2): 479– 91. doi:10.1002/ijc.28680. 24443327

[159] Vecchio D , Daga A , Carra E , Marubbi D , Raso A , Mascelli S , et al. Pharmacokinetics, pharmacodynamics and efficacy on pediatric tumors of the glioma radiosensitizer KU60019. Int J Cancer. 2015; 136( 6): 1445– 57. doi:10.1002/ijc.29121. 25091220

[160] McCabe N , Hanna C , Walker SM , Gonda D , Li J , Wikstrom K , et al. Mechanistic rationale to target PTEN-deficient tumor cells with inhibitors of the DNA damage response kinase ATM. Cancer Res. 2015; 75( 11): 2159– 65. doi:10.1158/0008-5472.CAN-14-3502. 25870146

[161] Fischer T , Hartmann O , Reissland M , Prieto-Garcia C , Klann K , Pahor N , et al. PTEN mutant non-small cell lung cancer require ATM to suppress pro-apoptotic signalling and evade radiotherapy. Cell Biosci. 2022; 12( 1): 50. doi:10.1186/s13578-022-00778-7. PMC9044846 35477555

[162] Mak JPY , Ma HT , Poon RYC . Synergism between ATM and PARP1 inhibition involves DNA damage and abrogating the G(2) DNA damage checkpoint. Mol Cancer Ther. 2020; 19( 1): 123– 34. doi:10.1158/1535-7163.MCT-19-0474. 31597711

[163] Hwang M , Jun DW , Song BR , Shim H , Lee CH , Kim S . *Ataxia*-telangiectasia mutated is involved in autolysosome formation. Biomol Ther. 2023; 31( 5): 559– 65. doi:10.4062/biomolther.2023.003. PMC10468418 36941082

[164] Xie Y , Ding J , Cui X , Wu M , Huang C , Zhang R , et al. Screening of kinase inhibitors downregulating PD-L1 expression via on/in cell quantitative immunoblots. Eur J Pharm Sci. 2020; 142: 105088. doi:10.1016/j.ejps.2019.105088. 31626963

[165] Callaghan CM , Abukhiran IM , Masaadeh A , Van Rheeden RV , Kalen AL , Rodman SN 3rd , et al. Manipulation of redox metabolism using pharmacologic ascorbate opens a therapeutic window for radio-sensitization by ATM inhibitors in colorectal cancer. Int J Radiat Oncol Biol Phys. 2023; 115( 4): 933– 44. doi:10.1016/j.ijrobp.2022.10.012. PMC9974877 36228747

[166] Ma Z , Dang R , Wu G . KU60019 inhibits ovarian cancer progression by targeting DGAT1/has-miR-1273g-3p axis. PLoS One. 2025; 20( 6): e0325213. doi:10.1371/journal.pone.0325213. PMC12186960 40554491

[167] Kuroda S , Urata Y , Fujiwara T . *Ataxia*-telangiectasia mutated and the Mre11-Rad50-NBS1 complex: promising targets for radiosensitization. Acta Med Okayama. 2012; 66( 2): 83– 92. doi:10.18926/AMO/48258. 22525466

[168] Węsierska-Gądek J , Heinzl S . Interactions between *Ataxia* Telangiectasia mutated kinase inhibition, poly(ADP-ribose) polymerase-1 inhibition and BRCA1 status in breast cancer cells. J Cancer Prev. 2014; 19( 2): 125– 36. doi:10.15430/JCP.2014.19.2.125. PMC4204161 25337581

[169] Reinecke M , Ruprecht B , Poser S , Wiechmann S , Wilhelm M , Heinzlmeir S , et al. Chemoproteomic selectivity profiling of PIKK and PI3K kinase inhibitors. ACS Chem Biol. 2019; 14( 4): 655– 64. doi:10.1021/acschembio.8b01020. 30901187

[170] Wang X , Hong Y , Meng S , Gong W , Ren T , Zhang T , et al. A novel immune-related epigenetic signature based on the transcriptome for predicting the prognosis and therapeutic response of patients with diffuse large B-cell lymphoma. Clin Immunol. 2022; 243: 109105. doi:10.1016/j.clim.2022.109105. 36055572

[171] Jin Z , Wang X , Li X , Yang S , Ding B , Fei J , et al. Development of a relapse-related RiskScore model to predict the drug sensitivity and prognosis for patients with ovarian cancer. PeerJ. 2025; 13: e19764. doi:10.7717/peerj.19764. PMC12352420 40821986

[172] Rathi S , Oh JH , Zhang W , Mladek AC , Garcia DA , Xue Z , et al. Preclinical systemic pharmacokinetics, dose proportionality, and central nervous system distribution of the ATM inhibitor WSD0628, a novel radiosensitizer for the treatment of brain tumors. J Pharmacol Exp Ther. 2024; 390( 2): 260– 75. doi:10.1124/jpet.123.001971. PMC11264258 38858089

[173] Garcia DA , Rathi S , Connors MA , Grams M , Vaubel RA , Bakken KK , et al. Modeling the acute mucosal toxicity of fractionated radiotherapy combined with the ATM inhibitor WSD0628. Mol Cancer Ther. 2025; 24( 2): 299– 309. doi:10.1158/1535-7163.MCT-24-0664. PMC11791477 39559836

[174] Riches LC , Trinidad AG , Hughes G , Jones GN , Hughes AM , Thomason AG , et al. Pharmacology of the ATM inhibitor AZD0156: potentiation of irradiation and olaparib responses preclinically. Mol Cancer Ther. 2020; 19( 1): 13– 25. doi:10.1158/1535-7163.MCT-18-1394. 31534013

[175] Dobler C , Jost T , Hecht M , Fietkau R , Distel L . Senescence induction by combined ionizing radiation and DNA damage response inhibitors in head and neck squamous cell carcinoma cells. Cells. 2020; 9( 9): 2012. doi:10.3390/cells9092012. PMC7563880 32883016

[176] Gill SJ , Wijnhoven PWG , Fok JHL , Lloyd RL , Cairns J , Armenia J , et al. Radiopotentiation profiling of multiple inhibitors of the DNA damage response for early clinical development. Mol Cancer Ther. 2021; 20( 9): 1614– 26. doi:10.1158/1535-7163.MCT-20-0502. PMC8650722 34158341

[177] Scheper J , Hildebrand LS , Faulhaber EM , Deloch L , Gaipl US , Symank J , et al. Tumor-specific radiosensitizing effect of the ATM inhibitor AZD0156 in melanoma cells with low toxicity to healthy fibroblasts. Strahlenther Onkol. 2023; 199( 12): 1128– 39. doi:10.1007/s00066-022-02009-x. PMC10673781 36229655

[178] Yilmaz U , Kamer D , Asik A , Kara HG , Gündüz C , Kamer S . The effect of the ATM inhibitor AZD0156 on the radiosensitivity of human breast cancer and lung fibroblast cells. J Cancer Res Ther. 2023; 19( 2): 203– 7. doi:10.4103/jcrt.JCRT_1540_20. 37006058

[179] Wong WK , Guerra Liberal FDC , McMahon SJ . DNA repair inhibitors potentiate fractionated radiotherapy more than single-dose radiotherapy in breast cancer cells. Cancers. 2022; 14( 15): 3794. doi:10.3390/cancers14153794. PMC9367425 35954456

[180] Zhou Q , Howard ME , Tu X , Zhu Q , Denbeigh JM , Remmes NB , et al. Inhibition of ATM induces hypersensitivity to proton irradiation by upregulating toxic end joining. Cancer Res. 2021; 81( 12): 3333– 46. doi:10.1158/0008-5472.CAN-20-2960. PMC8260463 33597272

[181] Dunlop CR , Wallez Y , Johnson TI , Bernaldo de Quirós Fernández S , Durant ST , Cadogan EB , et al. Complete loss of ATM function augments replication catastrophe induced by ATR inhibition and gemcitabine in pancreatic cancer models. Br J Cancer. 2020; 123( 9): 1424– 36. doi:10.1038/s41416-020-1016-2. PMC7591912 32741974

[182] Li H , Yang W , Zhang M , He T , Zhou F , G Herman J , et al. Methylation of TMEM176A, a key ERK signaling regulator, is a novel synthetic lethality marker of ATM inhibitors in human lung cancer. Epigenomics. 2021; 13( 17): 1403– 19. doi:10.2217/epi-2021-0217. 34558311

[183] Qin T , Mullan B , Ravindran R , Messinger D , Siada R , Cummings JR , et al. ATRX loss in glioma results in dysregulation of cell-cycle phase transition and ATM inhibitor radio-sensitization. Cell Rep. 2022; 38( 2): 110216. doi:10.1016/j.celrep.2021.110216. PMC8759735 35021084

[184] Zhou J , Zhang MY , Gao AA , Zhu C , He T , Herman JG , et al. Epigenetic silencing schlafen-11 sensitizes esophageal cancer to ATM inhibitor. World J Gastrointest Oncol. 2024; 16( 5): 2060– 73. doi:10.4251/wjgo.v16.i5.2060. PMC11099458 38764821

[185] Lee HHY , Chow KL , Wong HS , Chong TY , Wong AST , Cheng GHW , et al. Inhibition of aberrantly overexpressed polo-like kinase 4 is a potential effective treatment for DNA damage repair–deficient uterine leiomyosarcoma. Clin Cancer Res. 2024; 30( 17): 3904– 18. doi:10.1158/1078-0432.CCR-23-3720. PMC11369621 38848043

[186] Long X , Dai A , Huang T , Niu W , Liu L , Xu H , et al. Simultaneous delivery of dual inhibitors of DNA damage repair sensitizes pancreatic cancer response to irreversible electroporation. ACS Nano. 2023; 17( 13): 12915– 32. doi:10.1021/acsnano.3c05009. 37352467

[187] Jin WJ , Zangl LM , Hyun M , Massoud E , Schroeder K , Alexandridis RA , et al. ATM inhibition augments type I interferon response and antitumor T-cell immunity when combined with radiation therapy in murine tumor models. J Immunother Cancer. 2023; 11( 9): e007474. doi:10.1136/jitc-2023-007474. PMC10510866 37730275

[188] Zhang Q , Jiang L , Wang W , Huber AK , Valvo VM , Jungles KM , et al. Potentiating the radiation-induced type I interferon antitumoral immune response by ATM inhibition in pancreatic cancer. JCI Insight. 2024; 9( 6): e168824. doi:10.1172/jci.insight.168824. PMC11063931 38376927

[189] Jost T , Wachter M , Meidenbauer J , Fietkau R , Gaipl US . Inhibiting the DNA damage repair of HNSCC cells in combination with normo-fractionated radiotherapy influences clonogenicity, senescence and expression of NK cell activation markers. Sci Rep. 2025; 15: 31827. doi:10.1038/s41598-025-17858-6. PMC12397261 40883442

[190] Durant ST , Zheng L , Wang Y , Chen K , Zhang L , Zhang T , et al. The brain-penetrant clinical ATM inhibitor AZD1390 radiosensitizes and improves survival of preclinical brain tumor models. Sci Adv. 2018; 4( 6): eaat1719. doi:10.1126/sciadv.aat1719. PMC6010333 29938225

[191] Chen J , Laverty DJ , Talele S , Bale A , Carlson BL , Porath KA , et al. Aberrant ATM signaling and homology-directed DNA repair as a vulnerability of p53-mutant GBM to AZD1390-mediated radiosensitization. Sci Transl Med. 2024; 16( 734): eadj5962. doi:10.1126/scitranslmed.adj5962. PMC11064970 38354228

[192] Ran X , Wu BX , Shi M , Song L , Nixon K , Philip V , et al. CRISPR screen of druggable targets in small cell lung cancer identified ATM inhibitor (AZD1390) as a radiosensitizer. Int J Radiat Oncol. 2024; 118( 5): 1308– 14. doi:10.1016/j.ijrobp.2023.12.011. 38104868

[193] Talele S , Zhang W , Chen J , Gupta SK , Burgenske DM , Sarkaria JN , et al. Central nervous system distribution of the *Ataxia*-telangiectasia mutated kinase inhibitor AZD1390: implications for the treatment of brain tumors. J Pharmacol Exp Ther. 2022; 383( 1): 91– 102. doi:10.1124/jpet.122.001230. PMC9513858 36137710

[194] Ratz L , Brambillasca C , Bartke L , Huetzen MA , Goergens J , Leidecker O , et al. Combined inhibition of EZH2 and ATM is synthetic lethal in BRCA1-deficient breast cancer. Breast Cancer Res. 2022; 24( 1): 41. doi:10.1186/s13058-022-01534-y. PMC9206299 35715861

[195] Liu L , Hu X , Feng J , Lei A , Huang S , Liu X , et al. Suppression of DNMT1 combined with ATM or ATR inhibitor as a therapeutic combination of acute myeloid leukemia. Anticancer Drugs. 2024; 35( 3): 251– 62. doi:10.1097/CAD.0000000000001564. PMC10833198 38164802

[196] Zheng S , Song J , Linghu D , Yang R , Liu B , Xue Z , et al. Galectin-9 blockade synergizes with ATM inhibition to induce potent anti-tumor immunity. Int J Biol Sci. 2023; 19( 3): 981– 93. doi:10.7150/ijbs.79852. PMC9909994 36778120

[197] Colclough N , Orton AL , Martin S , Wild M , Reddy VP , Lenz E , et al. Achieving human brain exposure with the oral *Ataxia*-telangiectasia mutated kinase inhibitor AZD1390, a substrate of aldehyde oxidase. Drug Metab Dispos. 2025; 53( 8): 100107. doi:10.1016/j.dmd.2025.100107. 40714318

[198] Cai MY , Dunn CE , Chen W , Kochupurakkal BS , Nguyen H , Moreau LA , et al. Cooperation of the ATM and fanconi *Anemia*/BRCA pathways in double-strand break end resection. Cell Rep. 2020; 30( 7): 2402– 15.e5. doi:10.1016/j.celrep.2020.01.052. PMC8713357 32075772

[199] Turchick A , Zimmermann A , Chiu LY , Dahmen H , Elenbaas B , Zenke FT , et al. Selective inhibition of ATM-dependent double-strand break repair and checkpoint control synergistically enhances the efficacy of ATR inhibitors. Mol Cancer Ther. 2023; 22( 7): 859– 72. doi:10.1158/1535-7163.MCT-22-0685. PMC10320480 37079339

[200] Waqar SN , Robinson C , Olszanski AJ , Spira A , Hackmaster M , Lucas L , et al. Phase I trial of ATM inhibitor M3541 in combination with palliative radiotherapy in patients with solid tumors. Investig New Drugs. 2022; 40( 3): 596– 605. doi:10.1007/s10637-022-01216-8. PMC9098584 35150356

[201] Colclough N , Chen K , Johnström P , Fridén M , McGinnity DF . Building on the success of osimertinib: achieving CNS exposure in oncology drug discovery. Drug Discov Today. 2019; 24( 5): 1067– 73. doi:10.1016/j.drudis.2019.01.015. 30710641

[202] Qian C , Li X , Zhang J , Wang Y . Small molecular inhibitors that target ATM for drug discovery: current research and potential prospective. J Med Chem. 2024; 67( 17): 14742– 67. doi:10.1021/acs.jmedchem.4c01064. 39149790

[203] Brandsma I , Fleuren EDG , Williamson CT , Lord CJ . Directing the use of DDR kinase inhibitors in cancer treatment. Expert Opin Investig Drugs. 2017; 26( 12): 1341– 55. doi:10.1080/13543784.2017.1389895. PMC6157710 28984489

[204] Schmidt KM , Geissler EK , Lang SA . Subcutaneous murine xenograft models: a critical tool for studying human tumor growth and angiogenesis *in vivo*. Methods Mol Biol. 2016; 1464: 129– 37. doi:10.1007/978-1-4939-3999-2_12. 27858362

[205] Price JE . Xenograft models in immunodeficient animals: I. Nude mice: spontaneous and experimental metastasis models. Methods Mol Med. 2001; 58: 205– 13. doi:10.1385/1-59259-137-X:205. 21340860

[206] Abdolahi S , Ghazvinian Z , Muhammadnejad S , Saleh M , Asadzadeh Aghdaei H , Baghaei K . Patient-derived xenograft (PDX) models, applications and challenges in cancer research. J Transl Med. 2022; 20( 1): 206. doi:10.1186/s12967-022-03405-8. PMC9088152 35538576

[207] Liu Y , Wu W , Cai C , Zhang H , Shen H , Han Y . Patient-derived xenograft models in cancer therapy: technologies and applications. Signal Transduct Target Ther. 2023; 8( 1): 160. doi:10.1038/s41392-023-01419-2. PMC10097874 37045827

[208] Kersten K , de Visser KE , van Miltenburg MH , Jonkers J . Genetically engineered mouse models in oncology research and cancer medicine. EMBO Mol Med. 2017; 9( 2): 137– 53. doi:10.15252/emmm.201606857. PMC5286388 28028012

[209] Desjardins J , Cowan M , Yamanaka Y . Designing genetically engineered mouse models (GEMMs) using CRISPR mediated genome editing. Methods Mol Biol. 2022; 2429: 515– 31. doi:10.1007/978-1-0716-1979-7_36. 35507186

[210] Hill W , Caswell DR , Swanton C . Capturing cancer evolution using genetically engineered mouse models (GEMMs). Trends Cell Biol. 2021; 31( 12): 1007– 18. doi:10.1016/j.tcb.2021.07.003. 34400045

[211] Tsoli M , Wadham C , Pinese M , Failes T , Joshi S , Mould E , et al. Integration of genomics, high throughput drug screening, and personalized xenograft models as a novel precision medicine paradigm for high risk pediatric cancer. Cancer Biol Ther. 2018; 19( 12): 1078– 87. doi:10.1080/15384047.2018.1491498. PMC6301829 30299205

[212] Essers PBM , van der Heijden M , Verhagen CVM , Ploeg EM , de Roest RH , Leemans CR , et al. Drug sensitivity prediction models reveal a link between DNA repair defects and poor prognosis in HNSCC. Cancer Res. 2019; 79( 21): 5597– 611. doi:10.1158/0008-5472.CAN-18-3388. 31515237

[213] Whiteaker JR , Wang T , Zhao L , Schoenherr RM , Kennedy JJ , Voytovich U , et al. Targeted mass spectrometry enables quantification of novel pharmacodynamic biomarkers of ATM kinase inhibition. Cancers. 2021; 13( 15): 3843. doi:10.3390/cancers13153843. PMC8345163 34359745

[214] Cui S , Chen T , Zhao Y , Xiao Z , Liu M , Huang X , et al. Identification of ATM mutation as a potential prognostic biomarker for immune checkpoint inhibitors therapy. Curr Cancer Drug Targets. 2024; 24( 5): 501– 9. doi:10.2174/0115680096250376231025062652. 38804343

[215] Hu M , Zhou M , Bao X , Pan D , Jiao M , Liu X , et al. ATM inhibition enhances cancer immunotherapy by promoting mtDNA leakage and cGAS/STING activation. J Clin Investig. 2021; 131( 3): e139333. doi:10.1172/JCI139333. PMC7843232 33290271

[216] Li W , Li B , Cai C , Chen J , Li Y , Liang R , et al. Implications of DNA damage response and immunotherapy in tumor therapy. Cell Commun Signal. 2025; 23( 1): 518. doi:10.1186/s12964-025-02527-y. PMC12676849 41340123

[217] Ngoi NYL , Westin SN , Yap TA . Targeting the DNA damage response beyond poly(ADP-ribose) polymerase inhibitors: novel agents and rational combinations. Curr Opin Oncol. 2022; 34( 5): 559– 69. doi:10.1097/CCO.0000000000000867. PMC9371461 35787597

[218] Bhamidipati D , Haro-Silerio JI , Yap TA , Ngoi N . PARP inhibitors: enhancing efficacy through rational combinations. Br J Cancer. 2023; 129( 6): 904– 16. doi:10.1038/s41416-023-02326-7. PMC10491787 37430137

[219] Herbert KJ , Upstill-Goddard R , Dreyer SB , Rebus S , Pilarsky C , Debabrata M , et al. Sequential ATR and PARP inhibition overcomes acquired DNA damaging agent resistance in pancreatic ductal adenocarcinoma. Br J Cancer. 2025; 133( 3): 381– 93. doi:10.1038/s41416-025-03051-z. PMC12322129 40442403

