# Supplementary material for: Targeting ATM Kinase in Cancer—A Comprehensive Review
Source: Oncol Res. 2026 Oct 28;34(11):3. doi: 10.32604/or.2026.082180 (PMC13732375; doi:10.32604/or.2026.082180)
Supplement: Supplementary file 1 [file OncolRes-34-82180-s001.zip › TSP_OR_82180-s001.pdf]

Table S1. Key preclinical insights derived from studies involving use of KU-55933 as ATM inhibitor.

Model	Key Findings	PMID
Human tumor cell lines (U2OS, LoVo, SW620, HeLa B) the A-T fibroblast cell line (AT4), normal human fibroblasts (1BR), Chinese hamster cell lines V3 and V3YAC	KU-55933 potently and selectively inhibits ATM ($IC_{50} \approx 13$ nM), phenocopying genetic *ATM* loss; blocks IR-induced phosphorylation of TP53, γH2AX, NBS1, SMC1. KU-55933 suppresses IR-induced G1/S and G2/M checkpoints; sensitizes cells to IR and DSB-inducing agents; no effect in ATM-null cells	15604286
Breast cancer MCF7 cells	Pharmacological inhibition of ATM (KU-55933) or DNA-PKcs (NU7441) significantly sensitized breast cancer cells to IR. Radiosensitization correlated with a DNA repair defect, evidenced by persistent γH2AX foci following IR. ATM inhibition markedly reduced early γH2AX phosphorylation, whereas DNA-PKcs inhibition had a modest early effect. Combined inhibition strongly suppressed γH2AX formation, indicating partially redundant roles. DNA-PKcs inhibition produced greater radiosensitization than ATM inhibition alone, with no strong additive effect when combined, suggesting convergence on NHEJ-mediated repair. Residual late γH2AX phosphorylation suggests ATR compensation at later time points.	16293233
Human malignant glioma U87/DR-GFP cells	ATM kinase activity is essential for efficient HRR, with the ATM inhibitor KU-55933 reducing HRR by up to 90% in growth-arrested cells. ERK1/2 and JNK signaling positively regulate HRR, whereas P38 MAPK negatively regulates HRR. Oncogenic RAF-1 activation increases HRR, linking mitogenic signaling to DNA repair capacity. KU-55933 partially blocks radiation-induced ERK1/2 phosphorylation, indicating that ATM regulates ERK signaling. Inhibition of MEK/ERK signaling severely reduces ATM autophosphorylation (Ser1981) and ATM foci formation, without affecting γH2AX foci, suggesting selective impairment of ATM activation rather than DSB sensing. Findings support a bidirectional regulatory feedback loop between ATM and RAF/MEK/ERK signaling controlling HRR efficiency.	17283137
Fanconi anemia (FA) pathway–deficient human fibroblasts, pancreatic cancer cell lines (FANCG- and FANCC-mutant), murine embryonic fibroblasts, and *Fancg–/–Atm–/–* mice, analyzed using siRNA screening and the ATM inhibitor KU-55933	ATM is essential for the survival of FA pathway–deficient cells, identified through high-throughput siRNA screening. FA-deficient cells exhibit constitutive, low-level ATM activation and γH2AX formation, consistent with spontaneous S-phase DNA damage. Combined loss of ATM and FA pathway function is lethal, demonstrated by nonviability of *Fancg–/–Atm–/–* mice. Genetic or pharmacologic inhibition of ATM in FA-deficient cells induces DNA breakage, late S/G2 arrest, replication failure, chromosomal instability, and apoptosis. FA-deficient pancreatic tumor lines (HS766T [*FANCG*−] and PL11 [*FANCC*−]) are selectively hypersensitive to KU-55933 compared with isogenic corrected controls. ATM inhibition causes persistent unrepaired DSBs during replication specifically in FA-deficient cells, while FA-proficient cells tolerate ATM inhibition with minimal chromosomal damage. Data support a model in which ATM and the FA pathway function in parallel, compensatory DNA damage response pathways during replication stress.	17431503
Primary human fibroblasts from ataxia-telangiectasia (A-T) patients and wild-type controls, HeLa cells with RNR subunit	ATM regulates RNR, the rate-limiting enzyme for de novo dNTP synthesis, linking DNA damage signaling to nucleotide metabolism. Disruption of ATM signaling (genetic loss or KU-55933 inhibition) causes mtDNA depletion under normal growth conditions, phenocopying pharmacologic or RNAi-mediated RNR inhibition. *ATM* loss leads to global dysregulation of RNR subunits (R1, R2, and p53R2) and failure to upregulate mtDNA	17786248

knockdown, ATM inhibitor–treated cells (KU-55933), and *ATM*-null mouse tissues	following ionizing radiation. A-T fibroblasts exhibit elevated mtTFA/mtDNA ratios, indicating impaired coordination between mtDNA replication and transcription. *ATM*-deficient and RNR-impaired cells show increased resistance to inhibitors of mitochondrial respiration and translation, consistent with altered mitochondrial homeostasis. *ATM*-null mouse tissues display reduced RNR R1 expression and tissue-specific mtDNA copy number alterations, recapitulated in tissues from human A-T patients.	
Human DNA polymerase η (*POLH*/*POLη*)-deficient XP variant cells (XP30RO), isogenic *POLη*-complemented cells (TR30-9), normal human fibroblasts (GM00637)	Polη deficiency increases sensitivity to cisplatin and oxaliplatin, independent of drug uptake or total DNA platination, demonstrating a direct role for POLη in lesion tolerance rather than damage avoidance. Loss of *POLη* results in prolonged S-phase arrest following platinum treatment, consistent with defective TLS across platinum-induced DNA adducts. *POLη*-deficient cells exhibit enhanced PIKK signaling, including increased phosphorylation of CHK1, NBS1, and particularly RPA2. Platinum treatment induces ATR-dependent recruitment of RPA2 to chromatin, accompanied by phosphorylation on Ser33. Subsequent DNA-PKcs–dependent hyperphosphorylation of RPA2 on Ser4/Ser8 occurs after chromatin loading and is markedly elevated in *POLη*-deficient cells. ATM inhibition with KU-55933 does not block RPA2 Ser4/Ser8 phosphorylation and slightly increases overall RPA2 phosphorylation, consistent with increased persistence of replication-associated DNA breaks. Combined inhibition of ATM and ATR blocks both RPA recruitment and phosphorylation, indicating a sequential ATR to DNA-PKcs signaling axis at stalled or collapsed replication forks.	18289945
WRN short-hairpin RNA (shRNA), ATM shRNA and control cells using the U-2 OS osteosarcoma cell line	WRN is required for ATM pathway activation during replication-dependent DSBs generated by ICLs, but not for ATM activation by acute γ-irradiation. WRN depletion causes a defective intra–S-phase checkpoint following PUVA-induced replication fork collapse, characterized by continued DNA synthesis despite damage. Loss of *WRN* impairs ATM autophosphorylation at Ser1981 and reduces phosphorylation of downstream ATM substrates (SMC1, BRCA1, CHK1). Pharmacologic ATM inhibition with KU-55933 phenocopies *WRN* depletion, producing a similar S-phase checkpoint defect under replication stress. *WRN*-deficient cells display elevated and persistent γH2AX levels at later time points after PUVA treatment, indicating unresolved replication-associated DSBs and increased genomic instability. WRN and ATM colocalize at replication foci in unstressed cells, suggesting WRN acts upstream by facilitating ATM access or activation at collapsed replication forks. Data support a model in which WRN functions as a sensor or scaffold at replication intermediates, enabling ATM activation via replication-dependent DSBs, potentially in cooperation with the MRN complex.	18596239
Human multiple myeloma (MM) cell lines (ARK, LP-1, OPM-2, RPMI-8226, SKO007/J3); primary malignant plasma cells from MM patients (mainly smoldering MM); NK cells.	Low doses of clinically used MM drugs induce a DDR that leads to up-regulation of NK-cell–activating ligands, including both NKG2D ligands (MICA, MICB, ULBPs) and DNAM-1 ligands (PVR/CD155, Nectin-2). Drug-induced ligand up-regulation occurs at both mRNA and protein levels and is observed in MM cell lines and ex vivo primary plasma cells from patients. Increased ligand expression translates into enhanced NK-cell degranulation and cytotoxic activation, with NKG2D and DNAM-1 as the dominant triggering receptors. Ligand induction is abolished by ATM and ATR inhibition (caffeine, KU-55933), demonstrating dependence on ATM/ATR-mediated DDR signaling. Up-regulation of NKG2D and DNAM-1 ligands is preferentially associated with senescent MM cells, particularly those arrested in G2 phase, consistent with persistent DDR signaling. DNAM-1 ligand regulation (especially PVR) is identified for the first time as DDR-dependent, positioning DNAM-1 alongside NKG2D as a sensor of cellular stress. Bortezomib additionally reduces MHC class I	19098271

	expression, potentially relieving inhibitory NK signals, while doxorubicin and melphalan enhance NK sensitivity without altering MHC I.	
Human skin keratinocytes: HPV18-immortalized HK18 cells (p53-inactive); NF-κB–deficient keratinocytes (HK18/mIκB); ATM-deficient fibroblasts (GM05849);	Exposure to low-dose radiation (10 cGy) induces a radioadaptive survival response in human keratinocytes, conferring resistance to subsequent cytotoxic irradiation. LDR selectively enhances ATM phosphorylation and activates the MEK/ERK and NF-κB pathways, without activating JNK or P38 MAPK. NF-κB transcriptional activity is increased after LDR despite no increase in P65 or P50 protein levels, indicating rapid post-translational activation rather than transcriptional induction. ATM directly associates with NF-κB P65 in resting cells, and this interaction is markedly enhanced by LDR, identifying a noncanonical mechanism of NF-κB activation. Inhibition or knockdown of ATM (KU-55933, caffeine, siRNA) or MEK/ERK signaling abolishes LDR-induced NF-κB activation and eliminates the adaptive survival advantage. The radioadaptive response is absent in NF-κB–inhibited cells, establishing NF-κB as an essential effector of LDR-mediated protection. The ATM–MEK/ERK–NF-κB network operates independently of TP53, highlighting a distinct prosurvival DDR pathway in normal epithelial cells.	19324081
Human melanoma cell lines (LU1205/1205lu, WM9, WM35, HHMSX); normal fibroblasts (TIG3, MRC-5); ATM-deficient fibroblasts (GM02052)	ATM protein and basal ATM Ser1981 phosphorylation are elevated in several melanoma cell lines relative to normal cells, indicating constitutive ATM pathway activity. γ-irradiation further activates ATM, leading to downstream activation of TP53, NF-κB, and STAT3, pathways associated with survival and apoptosis resistance. Pharmacologic inhibition or genetic suppression of ATM (KU-55933 or ATM shRNA) suppresses TP53 and NF-κB signaling but paradoxically enhances surface expression of TRAIL receptor DR5. ATM inhibition down-regulates cFLIP, a key inhibitor of caspase-8 activation, thereby removing a major block to extrinsic apoptosis. Combined γ-irradiation plus ATM inhibition markedly sensitizes melanoma cells to exogenous TRAIL-induced apoptosis, including TRAIL-resistant HHMSX cells. ATM inhibition reduces STAT3 Tyr705 and Ser727 phosphorylation, diminishing STAT3 transcriptional activity and relieving STAT3-mediated repression of DR5 expression. Dominant-negative STAT3β recapitulates ATM inhibition effects by up-regulating DR5, down-regulating cFLIP, and enhancing apoptosis in vitro and in melanoma xenografts. These data define an ATM-dependent STAT3/NF-κB antiapoptotic axis that limits DR5 expression and enforces TRAIL resistance in melanoma.	19351839
Breast cancer cells (MDA-MB-231) and colorectal cancer cells (HCT-116)	siRNA silencing of RRM1 or RRM2 markedly sensitizes cancer cells to CPT, identifying RNR as a key modulator of Top1 inhibitor cytotoxicity. RRM2 knockdown increases DNA damage, evidenced by enhanced γH2AX formation, indicating defective repair of CPT-induced lesions. CPT transcriptionally up-regulates RRM1 and RRM2 and induces nuclear translocation of RRM2, suggesting an active role for RNR in the DNA damage response. CPT induces CHK1 activation, and CHK1 inhibition or CHK1 siRNA blocks RRM2 induction at both mRNA and protein levels. E2F1 is required for CPT-induced RRM2 up-regulation; CHK1 inhibition suppresses E2F1 induction, and E2F1 silencing prevents RRM2 induction. ATM and ATR act upstream of Chk1, as silencing of ATM or ATR or pharmacologic inhibition of ATM (KU-55933) blocks CHK1 activation and RRM2 up-regulation.RRM2 knockdown sensitizes cells to CPT despite continued DNA replication, allowing replication-dependent DNA damage to accumulate while impairing repair.	19416980
Breast cancer cells (MDA-MB-	ATM is required for full activation of AKT in response to insulin or IGF-I; KU-55933 blocks Akt phosphorylation	20053781

453) and prostate cancer cells (PC-3), A38 and A29 mouse embryonic fibroblasts	at Ser473 (PDK2 site) and Thr308 (PDK1 site, higher doses required). Cytoplasmic ATM–AKT signaling regulates cancer cell proliferation independently of nuclear DDR. KU-55933 induces G1 cell cycle arrest via downregulation of cyclin D1 translation, linked to inhibition of 4E-BP1 phosphorylation. KU-55933 triggers apoptosis under serum starvation, suggesting blockade of AKT-mediated survival pathways (potentially via FOXO1). KU-55933 completely prevents rapamycin-induced feedback activation of AKT, and combination treatment enhances apoptosis and proliferation inhibition relative to either drug alone. Higher basal AKT activity in cancer cells correlates with stronger antiproliferative effects of KU-55933.	
Dopaminergic rat neuroblastoma B65 cells	ATM inhibition (KU-55933) or partial ATM silencing (siRNA) did not alter overall cell cycle progression. Both treatments reduced cyclin A levels and pRB phosphorylation at Ser780. ATM inhibition decreased active TP53 (Ser15), BAX, and P21 expression. H_2O_2-induced pRB phosphorylation was prevented by KU-55933 or ATM siRNA, showing ATM-dependence.	20213763
Human lung cancer (A549, NCI-H23, and SK-MES-1), prostate cancer (PC3, 22RV1, and LNCap), and breast cancer (MCF-7) cells	IR activates AMPK robustly, independent of LKB1 status. ATM is an upstream activator: KU-55933 blocks IR-induced AMPK phosphorylation. AMPK activation occurs first in the nucleus, then extends to cytoplasm; nuclear–cytoplasmic shuttling is involved. IR-induced AMPK activation promotes TP53 and P21 induction. P21 induction occurs even in *TP53*-null cells. AMPK controls IR-induced G2/M arrest through P21; inhibition of AMPK (Compound C or AMPK α siRNA) abolishes this checkpoint. AMPK inhibition confers radioresistance, while AMPK activation (e.g., metformin) enhances radiosensitivity. Pharmacologic (metformin) AMPK activator that enhances IR-induced AMPK activation, reduces survival fraction, and may act as a radiosensitizer.	20615625
Glioma stem cells (GSCs) – BORRU (high stemness) and DR177 (low stemness)	ATM drives radioresistance in GSCs via constitutive activation of the DDR. ATM inhibition with KU-55933 or KU-60019 sensitizes high-stemness BORRU cells to IR and other DSB-inducing agents (e.g., CPT), but not nonstem or differentiated cells. Sensitization correlates with pushing cells into G2/M phase and reducing phosphorylation of downstream ATM targets: TP53, H2AX, and NBS1. KU-60019 is 10× more potent than KU-55933, with stronger radiosensitization at low doses and more specificity toward stem cells. Differentiated GSCs are resistant to ATM inhibitor–mediated radiosensitization, indicating stem-specific dependency on ATM. Inhibition of CHK1 sensitizes both BORRU and DR177, while CHK2 inhibition protects them, suggesting differential downstream signaling.	22257080
Breast cancer cells (MCF-10A, MCF-7) and immortalized human keratinocytes (HaCaT)	ATM kinase activity is autoregulated: inhibition with KU-55933 induces transcriptional upregulation and oscillation of ATM independent of cell cycle. KU-induced ATM induction is accompanied by transient increases in TP53, E2F1, and ATR, linking DDR signaling to ATM autoregulation. Mechanism involves negative feedback via TP53 (ATM suppressor) and positive feedback via E2F1 (ATM promoter activator). De novo ATM synthesis during KU treatment can escape inhibition due to reversible binding, restoring temporary DDR signaling. Reporter assays (ATM promoter constructs) confirmed KU-induced transcriptional activation across multiple cell lines. Nuclear phosphorylation of ATM, E2F1, and TP53 levels increase after DNA damage, but total ATM localization is largely unchanged.	22728709
Glioblastoma cell lines (U251, U87, GBM12) and TMZ-resistant	ATM inhibitors KU-55933 and CP-466722 selectively sensitize parental TMZ-sensitive GBM lines to TMZ but not TMZ-resistant lines. Sensitization is associated with increased G2/M cell cycle arrest in sensitive lines (e.g.,	23054561

derivatives (U251TMZ, U87TMZ, GBM12TMZ)	U251: 61.8% vs 35%) and increased residual γ-H2AX foci, indicating impaired repair of TMZ-induced DSBs. ATM inhibition did not block checkpoint activation (CHK1/CHK2 phosphorylation) or γ-H2AX foci formation, likely due to ATR compensation. TMZ resistance in U87TMZ/U251TMZ is not due to MGMT overexpression, whereas GBM12TMZ resistance is MGMT-mediated. Sensitization is *TP53*-status dependent, with stronger effects in *TP53*-deficient lines (U251) compared to *TP53*-wild-type lines (U87).	
Prostate cancer (PC-3) cells	ATM transcription is regulated by BRCA1, E2F1, and CtIP. BRCA1 binds *ATM* promoter and promotes transcription. CtIP acts as a coactivator, enhancing ATM transcription. E2F1 binds ATM promoter and, in the context of genotoxic stress, represses transcription. DNA damage by TOP inhibitors (doxorubicin, mitoxantrone) leads to repression of *ATM* transcription due to release of BRCA1 and CtIP while E2F1 remains bound. BRCA1 overexpression increases *ATM* transcription, which is reduced by BRCA1 depletion or BRCT domain loss. ATM kinase activity itself contributes to transcriptional regulation: KU-55933 decreases *ATM* promoter activity in *BRCA1*-overexpressing cells. Etoposide and methotrexate do not repress ATM transcription, highlighting agent-specific regulation.	22832221
Non-small-cell lung cancer (NSCLC) cell lines (A549, H460) & human fibroblasts (ATM-proficient 1BR3 / ATM-deficient AT5)	Cisplatin radiosensitization is cell-line dependent:<br>- H460: cisplatin enhances radiation sensitivity.<br>- A549: cisplatin alone does not radiosensitize due to cisplatin-mediated activation of ATM (Ser1981 phosphorylation) and AMPKα (Thr172) and induction of autophagy.<br>ATM inhibition (KU-55933) potentiates radiosensitization in both A549 and H460 cells. Autophagy inhibition (chloroquine/CQ) radiosensitizes both cell lines but does not affect cisplatin-mediated radiosensitivity pattern. AMPK inhibition (Compound C) radiosensitizes A549 cells but not H460, suggesting ATM/AMPK pathway mediates cytoprotective effects in A549. Combination of cisplatin + ATM inhibitor leads to synergistic radiosensitization in A549. Cisplatin-mediated radiosensitization is independent of apoptosis, *TP53* status, or direct impairment of DNA-DSB repair.	24857596
Glioma cell lines (U87MG, U251)	TMZ induces autophagy as a cytoprotective response. Autophagy induction is mediated by the ATM-AMPK-ULK1 signaling cascade. TMZ treatment increases AMPK phosphorylation and ULK1 activation, which are blocked by ATM inhibition (KU-55933). AMPK inhibition (compound C) reduces LC3B cleavage and AVO formation, indicating suppression of autophagy. Blocking autophagy via AMPK inhibition enhances TMZ cytotoxicity: ↓ cell viability, ↑ γH2AX-marked DSBs, ↑ apoptosis.	24737504
Patient-derived GBM CSCs vs differentiated tumour cells (R10, S2, E2, G7)	CSC-enriched cultures expressed higher levels of CD133, Nestin, SOX2, were more tumorigenic and invasive in vivo, and formed infiltrative tumours resembling human GBM. GBM CSCs were significantly more radioresistant than matched differentiated tumour cells. CSCs showed upregulated phosphorylated ATM and CHK22 at baseline and/or after irradiation. CSCs resolved γH2AX foci more efficiently, with fewer residual DSBs at 24 h post-irradiation. CSCs exhibited stronger and more sustained G2/M checkpoint arrest than differentiated cells. ATM inhibition potently radiosensitised CSCs and abolished their relative survival advantage. KU-55933 eliminated enhanced DSB repair proficiency in CSCs, increasing residual γH2AX foci at 24 h. G2/M checkpoint was almost completely abrogated in differentiated cells but only partially suppressed in CSCs. ATM inhibition equalised radiosensitivity between CSCs and differentiated tumour cells (notably in E2 cell line). ATM inhibition plus	25205037

	radiotherapy prolonged survival in orthotopic GBM models; normal brain tissue appeared less sensitized.	
Human gynecologic cancer cell lines (ovarian: A2780, A2780-CP20, OVCAR3; endometrial: KLE, HEC1B; cervical: HELA, SIHA; platinum-sensitive and -resistant; TP53 WT and mutant)	ATR inhibition, but not ATM inhibition, synergistically sensitized all GYN cancer cell lines to cisplatin and carboplatin, including platinum-resistant A2780-CP20 cells, via suppression of ATR-CHK1 signaling and increased apoptosis. ATM inhibition alone did not enhance platinum cytotoxicity, and co-inhibition of ATM+ATR did not exceed ATR inhibition alone. In contrast, inhibition of ATM or ATR each enhanced radiosensitivity, with maximal IR sensitization achieved by dual ATM+ATR inhibition, associated with loss of CHK1/CHK2 phosphorylation and impaired DNA damage checkpoint signaling.	25560806
Human bladder cancer cell lines (T24, 5637; DAB2IP knockdown via siRNA vs control)	Loss of *DAB2IP* confers radioresistance, marked by increased clonogenic survival after IR, elevated ATM expression and phosphorylation, expanded S-phase population, and accelerated DSB repair predominantly via HR (↑RAD51, ↓DNA-PKcs activity). Pharmacologic ATM inhibition with KU-55933 selectively radiosensitized *DAB2IP*-deficient cells by blocking ATM activation and downstream DDR signaling, slowing DSB repair, without significantly increasing apoptosis.	25585815
Breast cancer (MDA-MB-231) and prostate cancer (PC-3) celss	ATM functions as a positive regulator of insulin-stimulated glucose uptake in cancer cells by promoting GLUT1 translocation to the plasma membrane. ATM inhibition with KU-55933 blocks GLUT1 translocation, suppresses glucose uptake, aerobic glycolysis (Warburg effect) and ATP production, leading to apoptosis, reduced motility, and inhibition of EMT (↓vimentin). *ATM* knockdown phenocopies KU-55933 effects. In vivo, KU-55933 inhibits tumor growth and metastasis by suppressing GLUT1 trafficking and EMT markers.	33715230
Human liver cancer cell lines (Hep-G2, SMMC-7721)	Phenformin and KU-55933 synergistically suppress liver cancer cell proliferation and migration and induce apoptosis (synergy confirmed by CompuSyn). KU-55933 inhibits ATM phosphorylation and independently disrupts mitochondrial function (↓ATP, ↓mitochondrial membrane potential, abnormal morphology). Phenformin inhibits mitochondrial complex I, elevates AMP:ATP ratio, and activates AMPK. Combined treatment enhances AMPK activation while suppressing mTOR/p70S6K signaling, leading to profound mitochondrial dysfunction and energy collapse. KU-55933 counteracts phenformin-induced ATM activation, blocking DDR-mediated tumor cell survival and amplifying antitumor efficacy.	33742560
Human NSCLC cell lines with defined EGFR status: EGFR-mutant (PC-9, HCC827; exon 19 deletions) and EGFR wild-type (Calu-6, A549, H441, H596, etc.)	KU-55933 and gefitinib synergistically inhibit cell growth and induce apoptosis specifically in NSCLC cells harboring sensitive *EGFR* mutations, but not in *EGFR* wild-type cells. ATM inhibition enhances gefitinib-dependent repression of EGFR phosphorylation and downstream signaling (AKT, ERK). KU-55933 upregulates MIG-6, a negative regulator of EGFR signaling, in *EGFR*-mutant cells (e.g., PC-9), further dampening oncogenic EGFR signaling. ATM inhibition alone has limited effects but potentiates EGFR-TKI efficacy by suppressing EGFR pathway overactivation	26825989
Human endometrial cancer cell lines (Type I: Ishikawa, AN3CA, RL952, Hec-108; Type II: Hec-1B, KLE)	*ATM* alterations occur in ~20% of endometrial cancers and correlate with favorable prognosis. Low *ATM* expression confers intrinsic sensitivity to olaparib. Pharmacologic inhibition of ATM with KU-55933 significantly enhances olaparib-induced cytotoxicity, suppresses colony formation and migration, and increases apoptosis. Mechanistically, KU-55933 potentiates olaparib lethality by inhibiting ATM phosphorylation, thereby exacerbating HR deficiency and promoting synthetic lethality.	38144904
Human colon cancer cell lines	Inhibition of TRXR1 by auranofin induces ROS accumulation, which triggers compensatory activation of the	38565059

(HCT-116, HCT-15, DLD-1, LOVO)	ATM–AKT pathway marked by increased AKT phosphorylation. Dual inhibition of TRXR1 and AKT (auranofin + MK-2206) or TRXR1 and ATM (auranofin + KU-55933) produces strong synergistic cytotoxicity by further elevating ROS levels. ATM inhibition blocks auranofin-induced AKT phosphorylation, identifying ATM as an upstream regulator of AKT activation under oxidative stress. Combination treatments induce autophagy-dependent cell death, accompanied by ER stress (ATF4/CHOP) and JNK pathway activation.	

Abbreviations: ABCG2 – ATP-binding cassette subfamily G member 2; A-T – Ataxia-telangiectasia; AKT – Protein kinase B; AMPK – AMP-activated protein kinase; ATF4 – Activating transcription factor 4; ATM – Ataxia-telangiectasia mutated; ATP – Adenosine triphosphate; ATR – Ataxia-telangiectasia and Rad3-related; AVO – Acidic vesicular organelle; BRCA1 – Breast cancer gene 1; BRCT – BRCA1 C-terminal domain; cFLIP – Cellular FLICE-like inhibitory protein; CHK1/CHK2 – Checkpoint kinase 1/2; CHOP – C/EBP homologous protein; CPT – Camptothecin; CSC – Cancer stem cell; CtIP – C-terminal binding protein interacting protein; DAB2IP – Disabled homolog 2 interacting protein; DDR – DNA damage response; DNAM-1 – DNAX accessory molecule 1; DNA-PKcs – DNA-dependent protein kinase catalytic subunit; dNTP – Deoxyribonucleoside triphosphate; DR5 – Death receptor 5; DSB – DNA double-strand break; E2F1 – E2F transcription factor 1; EC – Endometrial cancer; EGFR – Epidermal growth factor receptor; EGFR-TKI – Epidermal growth factor receptor tyrosine kinase inhibitor; EMT – Epithelial–mesenchymal transition; ER – Endoplasmic reticulum; ERK1/2 – Extracellular signal-regulated kinase 1/2; FA – Fanconi anemia; FANCC/FANCG/FANCD2 – Fanconi anemia complementation group C/G/D2; FOXO1 – Forkhead box protein O1; GBM – Glioblastoma multiforme; GLUT1 – Glucose transporter 1; GSC – Glioma stem cell; GYN – Gynecologic; γH2AX – Phosphorylated histone H2AX; HR – Homologous recombination; HRR – Homologous recombination repair; HU – Hydroxyurea; IC_{50} – Half-maximal inhibitory concentration; IGF-I – Insulin-like growth factor I; IR – Ionizing radiation; JNK – c-Jun N-terminal kinase; LC3B – Light chain 3B (microtubule-associated protein); LDR – Low-dose radiation; LKB1 – Liver kinase B1; MAPK – Mitogen-activated protein kinase; MEK – Mitogen-activated protein kinase kinase; MGMT – O6-methylguanine-DNA methyltransferase; MGUS – Monoclonal gammopathy of undetermined significance; MHC – Major histocompatibility complex; MICA/MICB – MHC class I polypeptide-related sequence A/B; Mig-6 – Mitogen-inducible gene 6; MM – Multiple myeloma; MMC – Mitomycin C; MRN – MRE11-RAD50-NBS1 complex; mRNA – Messenger ribonucleic acid; mtDNA – Mitochondrial DNA; mtTFA – Mitochondrial transcription factor A; mTOR – Mechanistic target of rapamycin; NBS1 – Nijmegen breakage syndrome 1; NF-κB – Nuclear factor kappa-light-chain-enhancer of activated B cells; NHEJ – Non-homologous end joining; NK – Natural killer cell; NKG2D – Natural killer group 2 member D; NSCLC – Non-small cell lung cancer; PARP – Poly(ADP-ribose) polymerase; PCa – Prostate cancer; PDK1/PDK2 – Phosphoinositide-dependent kinase 1/2; PI3K – Phosphoinositide 3-kinase; PIKK – Phosphoinositide 3-kinase-related kinase; POLη (POLH) – DNA polymerase eta; pRB – Retinoblastoma protein (phosphorylated); PUVA – Psoralen plus ultraviolet A; PVR/CD155 – Poliovirus receptor; RNR/RR – Ribonucleotide reductase; ROS – Reactive oxygen species; RPA2 – Replication protein A2; RRM1/RRM2 – Ribonucleotide reductase subunit M1/M2; RT – Radiotherapy; SER – Sensitizer enhancement ratio; shRNA – Short hairpin RNA; siRNA – Small interfering RNA; SMC1 – Structural maintenance of chromosomes 1; STAT3 – Signal transducer and activator of transcription 3; TLS – Translesion synthesis; TMZ – Temozolomide; TOP1 – Topoisomerase I; TP53 – Tumor protein p53; TRAIL – Tumor necrosis factor-related apoptosis-inducing ligand; TRXR1 – Thioredoxin reductase 1; ULBPs – UL16 binding proteins; ULK1 – Unc-51-like autophagy activating kinase 1; WRN – Werner syndrome helicase; WT – Wild-type; 4E-BP1 – Eukaryotic translation initiation factor 4E-binding protein 1
